# An Effective and Automated Processing of Resonances in Vibrational Perturbation Theory Applied to Spectroscopy

**DOI:** 10.1021/acs.jpca.2c06460

**Published:** 2022-11-30

**Authors:** Qin Yang, Julien Bloino

**Affiliations:** † Faculty of Science, Scuola Normale Superiore, Piazza dei Cavalieri 7, I-56126Pisa, Italy; ‡ Institute of Organic Chemistry and Biochemistry, Czech Academy of Sciences, 16610Prague, Czech Republic

## Abstract

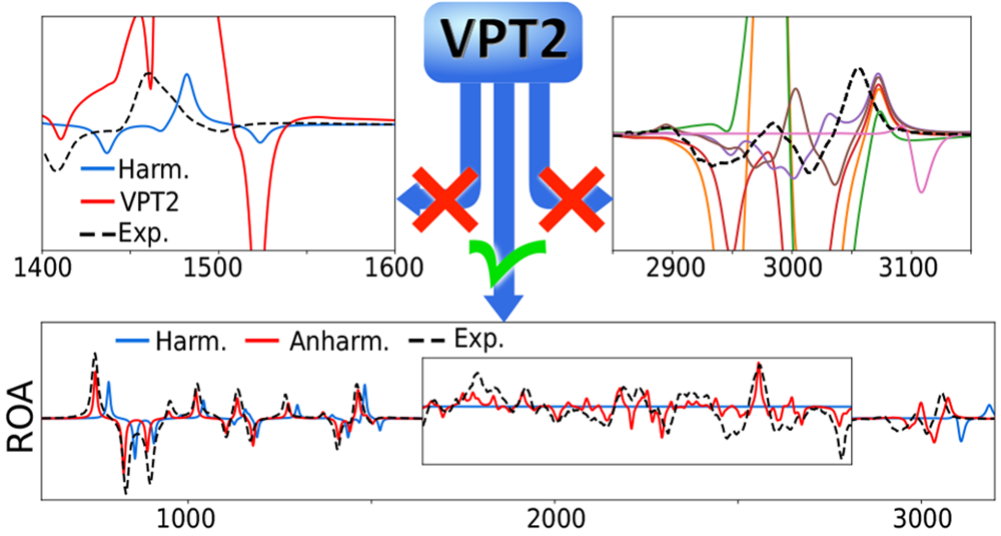

The broader availability of cost-effective methodologies like second-order vibrational perturbational theory (VPT2), also in general-purpose quantum chemical programs, has made the inclusion of anharmonic effects in vibrational calculations easier, paving the way to more accurate simulations. Combined with modern computing hardware, VPT2 can be used on relatively complex molecular systems with dozen of atoms. However, the problem of resonances and their corrections remains a critical pitfall of perturbative methods. Recent works have highlighted the sensitivity of band intensities to even subtle resonance effects, underlying the importance of a correct treatment to predict accurate spectral bandshapes. This aspect is even more critical with chiroptical spectroscopies whose signal is weak. This has motivated the present work in exploring robust methods and criteria to identify resonances not only in energy calculations but also on the transition moments. To study their performance, three molecules of representative sizes ranging from ten to several dozens of atoms were chosen. The impact of resonances, as well as the accuracy achievable once they are properly treated, is illustrated by the changes in spectral bandshapes, including chiroptical spectroscopies.

## Introduction

A detailed characterization of the structural parameters and physicochemical properties of molecules is often a preliminary step to understand their role and functions, either as a whole or as parts of more complex structures. This information can then help rationalize their activity in biological systems or their efficiency in technological applications, for instance.^1−5^ Vibrational spectroscopies are techniques of choice for such a task and can operate in a broad range of conditions to match closely those of the target compound, including its environment. Of course, to obtain a full and exploitable picture, a sufficient level of details in the recorded bandshape is necessary.

This has driven research toward the improvement of existing experimental setups, in terms of sampling conditions, for instance, to register the spectra of isolated molecules without the need for them to be in gas phase,^6,7^ as well as the refinement of instruments and measurement protocols.^8−10^ In turn, the gain in details of the experimental spectra, coupled to the use of more complex spectroscopic techniques whose signals result from the combination of multiple molecular properties like in chiroptical spectroscopies, has encouraged the recourse to numerical simulations to assist the interpretation of the outcome.^10−12^ This higher resolution can also underline limitations in the chosen theoretical models and the necessity for more sophisticated alternatives.^6,13,14^ The latter can then help highlight uncertainties in experimental data and potential sources of errors in the protocols, such as unsuspected environmental effects or actual constraints to the molecular structure.^15−20^ One consequence of this back-and-forth was highlighting the limit of the harmonic–oscillator approximation to represent vibrational motions. On the other hand, a proper treatment of anharmonicity comes with a significant price hike in terms of computational cost, which represents a barrier to the usage of such theoretical models.

Scaling factors offer a cheap way to account for anharmonicity and correct the band positions. However, their empirical basis makes them poorly suited to tackle complex molecular structures as well as predict the position of nonfundamental bands. Moreover, they are limited to vibrational energies and thus cannot compensate the errors and shortcomings from the harmonic approximation on the intensities. On the other end of the spectrum, variational approaches can predict very precisely the energy levels and intensities, on the condition that an extensive sampling of the potential energy surface (PES) is available.^21−31^ Even for limited accuracy requirements, within a few wavenumbers with respect to state-of-the-art experimental data, the number of points needed to build the PES remains high, and the variational calculation itself can be long. Mitigation techniques have been proposed, for instance, to reduce the number of points necessary on the PES^1^ or by truncating the variational problem,^32^ but the methods are generally expensive and mostly confined to small systems. Over the past decades, perturbative approaches based on the harmonic solution to the vibrational or rovibrational Schrödinger equation have been shown to be interesting alternatives, being able to capture the leading effects of the anharmonicity at a fraction of the cost.^20,33−39^ Among those approaches, the second-order vibrational perturbation theory (VPT2) is particularly effective,^40^ offering good performance while requiring limited knowledge of the PES around the equilibrium geometry.

Despite its successes, VPT2 suffers from two well-documented limitations, (i) a direct dependence on the harmonic approximation, which makes this methodology more suitable to treat rigid and semirigid molecular systems, and (ii) the problem of resonances, which can lead to the prediction of unrealistic energies and intensities. It should be noted that both problems are intrinsically related to the quality of the underlying electronic structure calculation method. The issue of the molecular flexibility and the presence of so-called large amplitude motions (LAMs) cannot be overcome purely within VPT2, and a proper solution requires alternative approaches, for instance, based on hybrid schemes capable of effectively separating vibrations based on their nature and affinity with flexible and rigid parts of the systems, each type being treated with suitable models.^41,42^ This aspect will not be discussed in this manuscript, as we will focus on sufficiently rigid molecules, which can be properly described with VPT2, of medium-to-large dimensions comprising even several dozens of atoms. The size poses obvious challenges regarding the problem of resonances and their correct identification and treatment. Indeed, as the number of vibrational modes grows, the possible combinations of resonant states explode. This is further complicated by the fact that those states can be involved in multiple resonances, creating a structure of interconnected states that need to be considered as a whole. As a result, simple approaches based on the manual identification of resonances, conceivable for small molecules, becomes quickly unfeasible for larger systems. Indeed, the existence of resonance clusters means that resonances would have to be identified iteratively to ensure that they are all identified and not hidden because of error compensations or unbalanced corrections. Besides the time consumption of such a procedure, this requires an extensive knowledge of the theoretical background and internal mechanisms of VPT2, restricting its application to a narrow audience. In order to tackle larger systems with more complex topologies but also in order to reliably and accurately predict different kinds of spectroscopies, robust methodologies to automatically identify the resonances are necessary.

The need for simple schemes, preferably based on few criteria, has led to the proposal of several methods in the literature,^39,43−48^ which can be implemented efficiently in computational chemistry software. It should be noted that most strategies focus on the problem of energies, obviously a primordial step to the simulation of vibrational spectra, while less effort has been devoted on the intensity. However, the correct prediction of band intensities has proven to be very challenging, especially when considering weak chiroptical spectral data, the result of the interaction of multiple properties.^6,18,49,50^ In this work, with particular attention to the problem of the reliable calculation of vibrational transition moments at the VPT2 level, we propose a new automated procedure to identify and correct resonances.

After a brief summary of VPT2 and the theoretical framework used by most implementations, the types of resonances and their effect on energy and intensity are described. This provides a basis to discuss different strategies applicable to identify the resonances and the origin of the new scheme proposed in this work. After some practical considerations on the implementation of our automated system to recognize, remove, and correct resonances, the new code is tested against a series of high-resolution experimental spectra of medium-large molecular systems. The impact of the threshold is also illustrated, and optimal criteria are proposed. In the concluding remarks, the reliability and robustness of such procedures, as well as the perspectives in terms of use of VPT2 and its variants by a broader scientific community, are discussed.

Among the molecular systems chosen here, methyloxirane will play a central role. Indeed, thanks to its relatively small size, it has been extensively studied, both experimentally and theoretically. Recently, high-resolution spectra have been recorded in Raman and infrared,^18,51^ providing high-quality reference data for an extensive numerical and visual analysis of the impact of resonances and their treatment. This will pave the way to the direct application on larger systems, pinene and artemisinin, where the best criteria found to automatically identify resonances will be further validated. Artemisinin, a well-known antimalaria medicine, has 7 chiral centers and a nontrivial structure that poses severe challenges to VPT2. It had been recently reinvestigated with chiroptical spectroscopies.^11^

## Theory

As usual, our starting point will be the Born–Oppenheimer approximation, and Eckart–Sayvetz conditions are assumed to be valid. The latter imposes a specific orientation of the molecular system under study. The vibrational and rotational wave functions will be considered separated with transitions of interest occurring here between purely vibrational levels. Our reference vibrational Hamiltonian is derived from the more general rovibrational expression proposed by Watson^52^ and has the form (in wavenumbers),
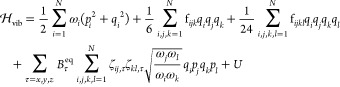
1where ***q*** is the vector of dimensionless normal coordinates, ***p*** is that of their conjugate momenta, and **ω** gathers the harmonic wavenumbers. ***B***^eq^ is the vector of equilibrium molecular rotational constants, and **ζ** is the matrix of Coriolis couplings. *U* is a mass-dependent contribution,^53^ which vanishes in the calculation of the transition energy and thus will be ignored in the following. Finally,  and  are the third and fourth derivatives of the potential energy, also known as the cubic and quartic force constants, respectively,
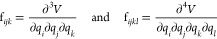


### A Brief Overview of VPT2

Complete derivations of full VPT2 equations can be found elsewhere,^40,54,55^ so we will focus here on the main aspects of interest for this work. Considering a generic quantity , its expectation value (*I* = *F*) or associated transition moment (*F* ≠ *I*) can be obtained from the resolution of the integral,
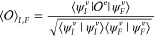
2where the subscript “e” denotes that  is actually an integral between electronic states. Since the focus here is on vibrational spectroscopies, this corresponds to the expectation value in a given electronic state, typically the ground state, so the superscript will be dropped, together with the “*v*”, in the following for the sake of readability. The vibrational wave functions, ψ_*I*_ and ψ_*F*_, are not necessarily normalized, as happens in the perturbed case.

Starting from the harmonic approximation, identified by a superscript “(0)”, the components in the right-hand side of eq 2 are expanded as perturbation terms,
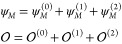


For the vibrational Hamiltonian, the perturbation terms are obtained by analogy with eq 1, giving
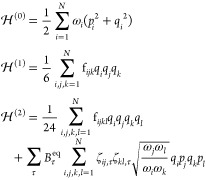


Two equivalent paths can be followed to obtain the vibrational energies at the VPT2 level, by means of the contact-transformation (contact-transformed Van Vleck perturbation theory, CVPT^56^) or the Rayleigh–Schrödinger perturbation theory (RSPT). The former proposes an elegant solution by transforming the Hamiltonian so that the resulting operator (noted ), applied to the harmonic wave functions, leads to the same formal expression of the eigenvalues as the original one with the perturbed wave functions. At the cost of some additional algebraic manipulations, this approach maintains a direct connection with the harmonic description of the vibrational states. The contact-transformed Hamiltonian also greatly simplifies the extension of VPT2 with tailored variational treatments, either to include corrections at higher order, for instance, through the inclusion of Darling–Dennison (DD) coupling terms,^57^ also known as Darling–Dennison resonances (DDRs), or to compensate the terms removed because they are found to be resonant.

While it is possible to compute the anharmonic corrections to the energy by replacing the terms in eq 1 with their numerical values and then carry out the transformation,^38^ the customary and computationally faster way is to derive an analytic formula applicable to any vibrational level. One convenient form is
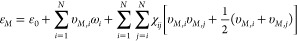
3where *v*_*M*,*i*_ represents the number of quanta associated with mode *i*. In the following, we will also adopt the Dirac notation to describe the vibrational states in the harmonic basis as vectors of quanta . ε_0_ is the anharmonic zero-point vibrational energy, which can be discarded by recasting the vibrational energies with respect to the ground state’s,
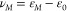


The anharmonic correction is governed by the **χ** matrix, whose elements are defined as
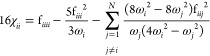
4
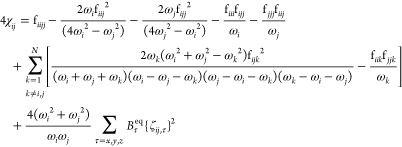
5

A similar protocol can be followed for the transition moments. In this case, each property of interest, represented as **P**, is expanded in the same way as before
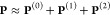


Likewise, by analogy with the harmonic approximation, **P**^(0)^ includes the equilibrium value and the first derivatives of the property, while **P**^(1)^ includes the second derivatives and **P**^(2)^, the third. Because properties can be of different shapes and defined with respect to the normal coordinates or their conjugate momenta, it is convenient to adopt a particular form of the Taylor series, valid for any property of interest,^45^
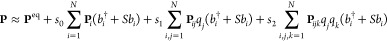
6with
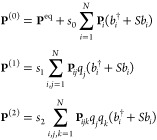
where  and *b*_*i*_ are the bosonic creation and annihilation operators. *S* holds the sign information and is equal to +1 for property functions of the normal coordinates and is otherwise −1. The other quantities are property dependent. *s*_0_, *s*_1_, *s*_2_ are constant factors, while the forms of the derivatives **P**_*i*_, **P**_*ij*_, and **P**_*ijk*_ depend on the properties. It is worth mentioning that the indexes may not fully permute (*S* = −1). Equivalency tables with actual quantities can be found in refs (45 and 58).

The derivation of analytic formulas can be done through the contact-transformation method in the same way as the energy.^38,59^ In this case, the transformation can be applied to the property or the wave function. Comparatively, RSPT offers a relatively straightforward way to build the closed-form formulas.^39,45,60,61^ A perturbation parameter (λ) is introduced and associated with each perturbative term, raised to a power matching the order of perturbation,
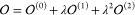


When **P** is introduced in place of  and the elements in the right-hand side of eq 2 are expanded, the terms are then gathered by the power of λ up to the second power, leading to the form
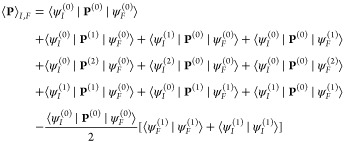
7

The first term corresponds to the harmonic level; those in the second line correspond to the first-order anharmonic correction and the rest, to the second order. The last term arises from the Taylor expansion with respect to λ up to the second order of the denominator.

The final step is the development of the perturbed wave functions in the harmonic basis,
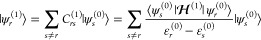
8
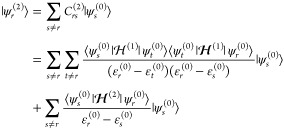
9

When eqs 8 and 9 are inserted into 7, a complete form to derive the equation for the transition moment of any property between arbitrary initial and final states is reached, explicitly written in eq S1.^45,60−62^ While it is not possible to derive a unique formula valid for any type of transition, relatively simple equations can be generated for groups of transitions, depending on the total number of quanta differing between the initial and final states. Considering only transitions from the ground state, the anharmonic transition moment for fundamental bands is given by
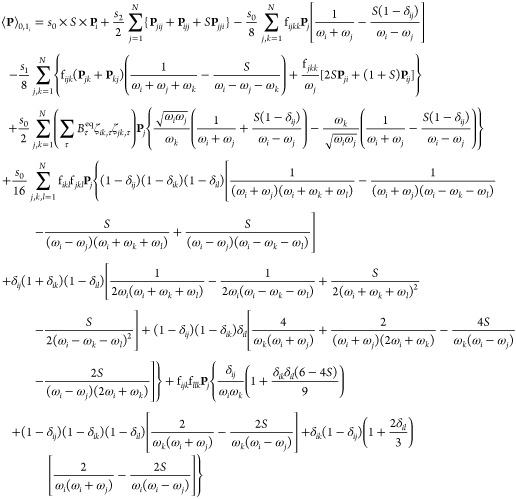
10with δ being the Kronecker symbol.

Similarly, the transition moment for first overtones and “1 + 1” combination bands is
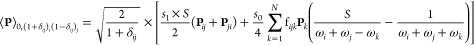
11

Transition moments for second overtones and combinations totaling up to 3 quanta are given in eq S6. To keep the discussion focused, the latter will not be explicitly addressed, and only the key aspects will be reported. It should be noted that, by construction, the equations for 3-quanta transition moments are similar to those of the fundamentals, so technical aspects regarding the latter can be quite straightfowardly extended to the former.

### Resonances in VPT2 Energies

From an investigation of eqs 4, 5, 10 and 11, conditions exist where the denominators can tend to 0, resulting in excessively large and unphysical contributions.

Let us first focus on the energy, where those so-called resonances have been extensively studied.^39,43,44,47,48,63−65^ The conditions can be sorted in two types, ω_*i*_ ≈ 2ω_*j*_ and ω_*i*_ ≈ ω_*j*_ + ω_*k*_. The first one is known as type I Fermi resonance (FR) and the second, as type II.^66^ Without a clear boundary between resonances and nonresonances, the definition of formal rules, necessary to build robust methodologies and ensure the consistency of VPT2 results over a broad range of systems, remains an open challenge. However, this is a prerequisite to design automated or semiautomated procedures capable of searching systematically resonances in larger molecular systems where the sheer number of combinations make any manual work impractical. Common strategies use two steps, first selecting relatively close states based on a relation of the form
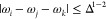
12where *j* and *k* can be equal and Δ^1–2^ can be chosen to be rather large, commonly a few hundred wavenumbers. This step has two benefits. It is a fast test, which can be used to quickly shorten the list of potential resonances among all possible combinations. It also reduces the risk of false positives “far from resonance” (ω_*i*_ ≫ ω_*j*_ + ω_*k*_). The second test regards the identification of the resonances themselves. Multiple schemes have been proposed. A selection of interesting criteria will be presented here.

A popular test is the one proposed by Martin et al.,^43^ which analyses the deviation of each potentially resonant term from a variational solution. Briefly, the basic principle is to consider each term individually. When it is excluded from the VPT2 treatment, a “deperturbed” energy is obtained. The full energy can then be obtained by building a 2 × 2 matrix with the deperturbed energies of the states involved in the potential resonance on the diagonal and the off-diagonal term corresponding to the integral of the contact-transformed Hamiltonian between those states of the form . The resulting eigenvalues can then be expanded as a Taylor series. When the VPT2 and “variational” energies are compared with respect to the deperturbed ones, a qualitative measure of the magnitude of the Fermi resonances is obtained, which can be written in a general form
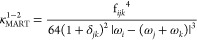
13Non-null values of κ indicate a potential impact of Fermi resonances. A typical threshold is 1 cm^–1^, but values as low as 0.1 have also been proposed.^39^ In the following, this scheme will be labeled “**R12MART**”.

Another parameter proposed by Krasnoshchekov et al. was derived from their numerical-analytic formulation of the canonical Van Vleck perturbation theory. Instead of using fully derived forms of the energies like in eq 3, their approach implements the elements of the contact transformation in terms of creation and annihilation operators (CAOs). The components of the vibrational Hamiltonian are then replaced by their numerical values for each application, and the full energy built progressively. By monitoring the evolution of the numerical contact-transformed Hamiltonian, they established empirical conditions of divergence related to Fermi resonances^67^
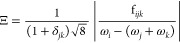
14

The original proposed threshold was 0.2 with acceptable values between 0.05 and 0.5. By comparing the evolution of this quantity with the perturbation parameter (λ) expressed in a complex form, they recently proposed a threshold of 0.0756.^47^ This scheme will be labeled “**R12CVPT**” here. For consistency with the other schemes,  will be used instead of Ξ to refer to the quantity used as the resonance criterion. In their original paper, they also compared this threshold with RSPT-derived terms discussed below.

In order to provide more accurate vibrational energies, most implementations also include the so-called Darling–Dennison couplings or resonances (DDRs).^57^ These do no directly impact the VPT2 energies in the manner of Fermi resonances but instead enable accounting for couplings normally ignored within VPT2. From a theoretical perspective, they can be described as interaction terms between states involving the second-order contact-transformed Hamiltonian. While they were originally referring to couplings between first overtones in the water molecules (simply noted here 2–2 with reference to the number of quanta differing for each mode between the bra and ket), they can be generalized to more types of interactions, between fundamentals (1–1 resonances), first overtones and binary 1 + 1 combinations (2–11) or between combinations (11–11), and finally between fundamentals and 3-quanta states (1–3, 1–21, 1–111). The derivation of the relative equations is rather cumbersome, and complete formulas were proposed by Rosnik and Polik^68^ for most common transitions considered in VPT2 calculations involving up to four quanta of difference, later reviewed by Krasnoshchekov et al.^67^ and Douberly and co-workers.^39^ A generalization to non-Abelian symmetry groups was recently achieved for transitions up to two quanta.^69^ Darling–Dennison terms are normally added after the actual VPT2 calculations through a tailored variational step described later. Because they are not critical to VPT2 like Fermi resonances, less work has been devoted to their identification. A simple strategy, commonly adopted and labeled in the form “**R*****AB*****HRS**”, is to select the closest states in energy, in a way similar to what is done for Fermi resonances, and then compute the actual coupling term, written in the contact-transformed formalism as
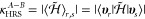
15with  being the contact transformed Hamiltonian.

Coupling terms  with a magnitude larger than a given threshold, noted *K*^*A*–*B*^ with “A–B” representing the type of DD resonance (1–1, 2–2, ...), are then added to the variational correction. A potential issue in the definition of reliable values for *K*^*A*–*B*^, discussed by Krasnoshchekov et al.,^67^ regards the case of 1–1 resonances between hotbands, where the coupled states share modes in common. Indeed, the analytic formula for  (***v*** ≠ **0**) is composed of three terms, weighed by the number of quanta associated with *i*, *j*, and all other modes in ***v***. To circumvent this limitation, they proposed an alternative criterion, derived from their CAO-based methodologies.^67^ When hot bands are ignored, which will be the case here, the resonance criterion is the same as in **R11HRS** discussed above.

As a final remark regarding Darling–Dennison couplings, a further complication to their calculation lies in the existence of conditions similar to Fermi resonances. The method chosen here, and recalled in the Supporting Information (“Resonances in Darling–Dennison terms”), is derived from the so-called hybrid degeneracy-corrected VPT2 (HDCPT2),^65^ so all possible resonant terms can be rewritten in a nonresonant form.^46^

### Resonances in Transition Intensities

While resonances in energy have been extensively studied, this has not been the case for intensities. Like energies, the transition moments, as evident from eqs 10 and 11 (and eq S6), can be affected by Fermi resonances. However, and contrary to the former, Darling–Dennison resonances have a direct impact and, if not treated, can lead to an incorrect evaluation of the anharmonic correction to the intensities. More specifically, fundamentals depend on 1–1 DDR, while 3-quanta transitions can be impacted by 1–3 DDR, where 1–3 is used to generically refer to 1–3, 1–21, and 1–111 cases. As a consequence, their correct identification is more critical than with energies.

Let us first start with Fermi resonances. While the same selection criteria as those used for the energy can be employed, they may not be able to identify cases of resonances specific to intensities. For instance, Martin’s test is rooted on the vibrational energy and emphasizes the strength of the coupling between the resonant states. Because of the different nature of the terms in the analytic formulas for the energy and intensity, common thresholds for the test (*K*^1–2^ ≥ 1) may fail to capture important resonances. Such shortcomings have already been observed in the literature and generally fixed manually or through *ad hoc* schemes.^49−51^ Alternatively, lower thresholds could be used^39^ with the risk of overcorrecting by defining an excessively large number of resonances, especially with larger systems. Since a common pattern in resonances ignored with higher thresholds is a low coupling (small ) between states very close in energy (|ω_*i*_ – (ω_*j*_ + ω_*k*_)| ≃ 1), a possible solution would be to add another selection criterion, emphasizing the proximity in energy, for instance, by weighing the test in eq 13 with the energy difference, . While such a test, labeled “**R12WFRQ**” in the following, could compensate the shortcomings of the original model, it remains associated with the energy and ignores the characteristics of the VPT2 analytic equations of the transition moments.

Let us consider the formula for the transition moment of fundamentals, given in eq 10, and more specifically the term
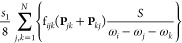
16From eqs 7–9, combined as eq S1, this term, which involves the second derivatives of the property (**P**^(1)^) and the cubic force constants, is related to . Similarly, for 2-quanta transitions (eq 11), the term
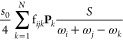
17involves the first derivative of **P** (**P**^(1)^) and one set of cubic force constants as well, so it is related to . In both cases, the first-order perturbed wave function  is involved. Within the summations in eqs 16 and 17, the same kind of coefficient is found, of the form
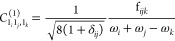
18Hence, since the resonance is directly connected to the mechanical anharmonicity, a good measure of the impact of the Fermi resonance would be to evaluate the coefficients of the first-order wave function and check that their value is not too large. Incidentally, this test is very close to “**R12CVPT**”, and this similarity had already been noted by the original authors, together with the factor difference of  for the overtones.^67^ While the difference is expected to be minimal, the present criterion has the advantage of fully accounting for the nature and form of the transition moments at the VPT2 level. The test, labeled “**R12COEF**”, can thus be summarized as
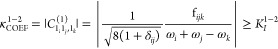


As already mentioned, Darling–Dennison resonances need to be separated at this level in two groups, those with no impact on the transition moments, between overtones or “1 + 1” combinations (2–2, 2–11, 11–11), which can be ignored here, and those with direct influence. For the latter, the same issue as with Fermi resonances occurs with the different forms of the potentially resonant terms between energy and intensity limiting the efficiency of tests tailored for the former. A mitigation strategy, named here **R*AB*WFRQ**, proposed in ref (58), was to weigh the Darling–Dennison term by dividing it by the square energy difference between the potentially resonant states, leading to the index
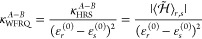
A lower threshold is used compared to **R*AB*HRS**, generally 1 order of magnitude lower. This test puts an emphasis on very close states, which are not strongly coupled (small ). It is especially effective for nearly degenerate modes for which it was primarily devised. Typical examples are CH symmetric and antisymmetric stretchings in methyl groups not strongly affected by the rest of the molecules. The two vibrations can have frequencies within a few wavenumbers and need to be properly treated to avoid the prediction of excessively intense bands in the CH fundamental regions.

Like Fermi resonances, such a test, while trying to cope with the shortcomings of a scheme designed for the calculation of vibrational energies, still loosely relates to the transition moments. Considering the formula for fundamentals, in eq 10, terms with frequency differences between harmonic fundamental states at the denominator involve the first derivatives of the property (**P**^(0)^) and elements of the second-order Hamiltonian. On the basis of eq 7, the origin in the perturbative expansion is . From the definition of , the coefficient of interest here is thus
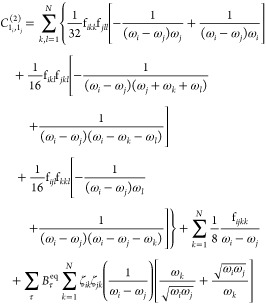
19A better identification of resonant terms in the transition moments to fundamentals would be achieved by assessing the magnitude of the second-order coefficient. The new test, **R11COEF**, thus relies on the absolute value of the term
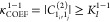
A similar test, **R13COEF**, can be derived for 3-quanta transitions. A generic formula for the coefficients is provided in eq S7. Like Darling–Dennison terms, the coefficients can themselves be impacted by resonances. In the same way, the HDCPT2 scheme was applied to prevent this condition.

It is worth mentioning that the perturbation should be small compared to the harmonic reference; the coefficients,  and  should be smaller than 1, providing an indicative upper bound for the thresholds  in **R*AB*COEF**.

As a final comment, slightly different formulas need to be used for the transition moments in the presence of a Darling–Dennison resonance. Indeed, when constructing the final analytic forms, some terms cancel each other, a situation not found if one of the terms in eq 7 is discarded. Resonant and nonresonant versions for the transition moments have been given in refs (48 and 69) and included in eqs S8 and S9.

### Implementation of the Tests and the GVPT2 Scheme

After this short overview of possible strategies and criteria to identify *a priori* resonances, let us look at the implementation part. The computation of anharmonic energies and intensities within the deperturbed VPT2 (DVPT2) scheme can be roughly divided in four main steps: (i) data extraction and preparation, (ii) *a priori* resonance analysis, (iii) energy calculation, and (iv) calculation of the transition moments. The exclusion of resonant terms leads to a truncated result, which can be improved by adding a variational step to correct the energies and successively the intensities. In this generalized form (GVPT2), the third step is extended with the construction of the variational correction. The resulting eigenvectors are then used to correct the deperturbed transition moments by projecting them onto the final states in the last step.

In our implementation, harmonic and anharmonic data can be provided through binary files generated by a quantum chemical program, here Gaussian, or as data sets in formatted text provided in the input by users or scripts. Different sources can be provided for harmonic and anharmonic data. This is usually done to balance accuracy and computational cost when the level of electronic structure calculation chosen for the harmonic description is too high to build the anharmonic constants, generally done by numerical differentiation. In these conditions, a trade-off can be considered by using a lower level to generate the anharmonic data. If the equilibrium geometries and normal modes are provided for each level, a consistency check can be performed using the transformation proposed by Duschinsky.^70^ Small structural differences and a very high overlap between the modes should be obtained to proceed with the computations. Besides levels of theory, data sets can be combined, for instance, to complement missing data from others. This can be used in bottom-up models in reduced-dimensionality schemes, where a subset of modes is first chosen to build the anharmonic force field and property surface through numerical differentiations. These modes, which are called active, can be related to a region of the molecule or an energy range. By then investigating the couplings of these modes with others, new elements may be added to the set of modes to treat anharmonically until no meaningful contribution remains between the selected modes and those ignored.^46,71^ To avoid redundancy, previously used anharmonic constants are not recomputed at each iteration, and new sets are built and then automatically combined within the program.

To facilitate the use of VPT2 on large systems, the identification of resonances is done automatically by default, starting from Fermi resonances, and then Darling–Dennison resonances. For each one, the procedure is relatively similar and centers on the construction of two data structures, “ResonanceDB” and “PolyadDB”. The first one lists all resonances by mode and type of resonance. It is then used during VPT2 calculations to flag resonances. The second is optional and activated only if a variational correction has been requested (GVPT2). This database contains all the information on the interconnection between resonant states and the variational term.

The selection process is the same for each resonance (Fermi, 1–1 DDR, 2–2 DDR, and optionally 1–3 DDR) and made of a few steps. First, a generator builds the possible combinations of resonances and checks the relative energy difference. If it is below a set threshold, Δ^*A*–*B*^, the combination is selected for further testing. Depending on the protocol chosen, one or two tests are used, either purely based on the energy or complemented with a scheme more suitable for intensity. If any test is passed, the resonance is inserted into “ResonanceDB”. For GVPT2, if all modes are included in the anharmonic treatment (active modes), information on the states in resonance and the associated variational correction are stored in “PolyadDB”. The automatic procedure can be deactivated or the generated list of resonances can be fully replaced or amended by the users, who can selectively remove or add resonances.

The elements of the anharmonic matrix **χ** are then calculated, removing all Fermi resonances from the summations, and deperturbed (DVPT2) energies are generated. For GVPT2, this is followed by the variational correction. In its simplest form, a null square matrix is built, with the length of each side equal to the number of states considered in the VPT2 treatment (of dimension *N* + *N*(*N* + 1)/2 or *N* + *N*(*N* + 1)/2 + *N*(*N* + 1)(*N* + 2)/6 for up to 3 quanta). The diagonal is then populated with the DVPT2 energy of each state, and the terms corresponding to Fermi resonances or Darling–Dennison couplings added off-diagonal. The matrix is then diagonalized with the eigenvalues forming the GVPT2 energies and the eigenvectors providing the transformation from DVPT2 to GVPT2 states. Because the matrix is normally very sparse, this approach is not efficient in terms of memory, as it scales with the number of modes to the power of 4 or 6. Moreover, it can introduce some spurious numerical errors in the diagonalization process. A more efficient way is to build small, independent matrices based on the polyads stored in “PolyadDB”. The relevant DVPT2 energies are added to each matrix, which is then diagonalized. In addition to a significant reduction of the memory requirements, the construction of the polyads can be easily done in parallel.

For each property of interest, the transition moments to all relevant states are then built, taking into account the list of resonances in “ResonanceDB” to choose the correct version of the VPT2 formulas. The resulting deperturbed integrals are then projected onto the final GVPT2 states by default.

Let us conclude this part on the implementation with a few practical comments. By default, with anharmonic constants built through two-step numerical differentiations, only diagonal second-order derivatives can be computed. This means that only the semidiagonal quartic force field  and third derivatives of the properties (**P**_*ijj*_) are available. This means that 11–11 Darling–Dennison resonances cannot be calculated properly, since they involve the off-diagonal term . To prevent an incorrect account of the correction, those resonances are ignored by default in the automatic treatment. The second issue, related to the property, only impacts 3-quanta transitions. For property function of the normal coordinates, like the electric dipole, this only affects “1 + 1 + 1” combination states . For those related to their conjugate momenta, like the atomic axial tensor (vibrational circular dichroism), the problem already appears for “2 + 1” combination states because of the noncommutation of some of the indexes, leading to some elements being unavailable. As a result, the contribution of the electrical or property-related anharmonicity may be missing or incomplete. The impact on the overall anharmonic correction and the reliability of the results should be carefully assessed.

Another comment regards the implementation of the **R*AB*COEF** tests. To provide a more intuitive understanding of the states involved in the transitions at the origin of interesting features in the spectral bandshape, it is convenient to build a pseudoeigenvector (**L**) of the perturbed wave function using the coefficients given in eqs 8 and 9. For fundamentals, they can be defined as
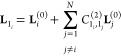
20the first-order coefficient giving no contribution by definition.  is a column from the matrix of eigenvectors of the harmonic Hessian matrix and describes the mass-weighted normal coordinates *Q*_*i*_ with respect to the Cartesian coordinates.

Similarly, 2-quanta states are defined as
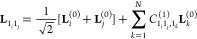
21

Hence, the perturbative coefficients can be calculated once, while constructing the list of resonances, and stored for later use, limiting the burden in terms of computational cost from the intensity-centric test.

## Computational Details

For all anharmonic calculations, except where specified otherwise, the B3PW91 exchange-correlation functional^72^ was employed, augmented by Grimme’s empirical dispersion scheme^73^ together with Becke–Johnson damping,^74^ B3PW91-D3(BJ). It has been paired with the “seasonal” basis set, jun-cc-pVTZ,^75^ which offers a similar performance to aug-cc-pVTZ^76^ for a notably lower cost. Where possible, the overall accuracy can be improved by replacing the harmonic force field or even the harmonic property surfaces with data from a higher level of theory. Here, this was done by employing the double hybrid functional, revDSD-PBEP86-D3(BJ).^77^ It should be noted that the use of such a hybrid scheme poses the problem of data consistency between different levels of electronic structure calculations. Indeed, in the strategy followed in this work, the calculations at different levels of theory are done separately and independently of one another, so that the lower-level anharmonic field is built by numerical differentiation using only reference data obtained at this level. To ensure that the calculations are valid, a simple check based on the Duschinsky transformation^70^ is performed, where the lower-level normal coordinates are expressed on the basis of the higher-level ones, used as reference^46,48^
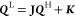
where ***Q***^L^ and ***Q***^H^ are the mass-weighted normal coordinates obtained from the diagonalization of the harmonic force constants at the lower and higher levels of theory, respectively. The elements of the shift vector ***K*** should be negligible, which means that there is no noticeable differences between the equilibrium structures. In practice, any geometrical change that is not a rotation or translation (nullified in Eckart orientation) would affect the normal coordinates due to their sensitivity, so ***K*** is not directly used. With the Duschinsky matrix **J** being orthogonal, the sum of square elements along each row and column is unity. The normal modes are considered equivalent if there is exactly 1 element per column/row with a squared value greater than 0.9 .

The semidiagonal quartic force field and semidiagonal third derivatives of the properties are built by numerical differentiations of analytic Hessians and first derivatives of the properties computed at displaced geometries from the equilibrium geometry. Reaching the true minimum of the potential energy surface (PES) is thus essential to avoid or minimize the errors in the derivatives. To meet this condition, very tight convergence criteria were used, which means in practice that the maximum forces and displacements with respect to the previous step were smaller than 5 × 10^–6^ Hartrees/Bohr and 2 × 10^–5^ Å, respectively. To build the anharmonic constants, fixed steps of  along the mass-weighted normal coordinates^44^ were used. In practice, this means that “2*N* + 1” harmonic force constants and property calculations are necessary. It is noteworthy that some energy and property derivatives are computed numerically multiple times. For instance, if modes *i*, *j*, and *k* are different, then  is obtained by displacement along *Q*_*i*_, *Q*_*j*_, and *Q*_*k*_. This property is used internally by the code to check the consistency of the numerical derivatives. Non-negligible fluctuations may reflect the failure to reach the true minimum, in particular for flat PESs, instabilities in the electronic structure calculation, or in some cases, problems related to the construction of the cavity for the polarizable continuum model (PCM) around the solute’s displaced geometry. For this work, variations of the anharmonic force constants below 1 cm^–1^ were considered acceptable and anharmonic calculations only proceeded if this was the case. Once all anharmonic constants were built, vibrational energies and intensities were computed within the so-called generalized VPT2 scheme (GVPT2), following the protocol described in the previous section.

Solvent effects have been taken account through the polarizable continuum model within its integral equation formalism (IEF-PCM)^78,79^ with default parameters as implemented in Gaussian.^80^ Geometry optimizations, harmonic frequency calculations, and the generation of anharmonic constants were done with Gaussian 16.^80^ A locally modified version of Gaussian was used to test the different resonance identification schemes and compute the resulting GVPT2 frequencies and energies. The data processing and generation of figures was done through an in-house tool, ESTAMPES,^81^ written in Python.

The schemes used in this work are summarized in Table 1.

**Table 1:** Parameters Used for the Automatic Identification of Resonances

scheme	test quantity	threshold
Fermi		
all	|ω_*i*_ – (ω_*j*_ + ω_*k*_)|	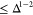
**R12MART**	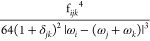	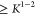
**R12CVPT**	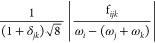	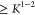
**R12WFRQ**	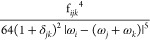	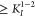
**R12COEF**	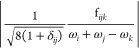	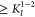
1–1 Darling–Dennison		
all	|ω_*i*_ – ω_*j*_|	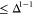
**R11HRS**	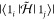	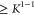
**R11WFRQ**	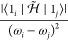	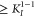
**R11COEF**	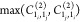	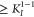
2–2 Darling–Dennison		
**R22HRS**^a^	|(ω_*i*_ + ω_*j*_) – (ω_*k*_ + ω_*l*_)|	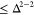
	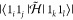	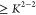

a Only 2–2 (*i* = *j*, *k* = *l*) and 2–11 (*i* = *j*, *k* ≠ *l*) resonances were considered. See text for details.

## Results and Discussion

### Methyloxirane

**Figure 1:**
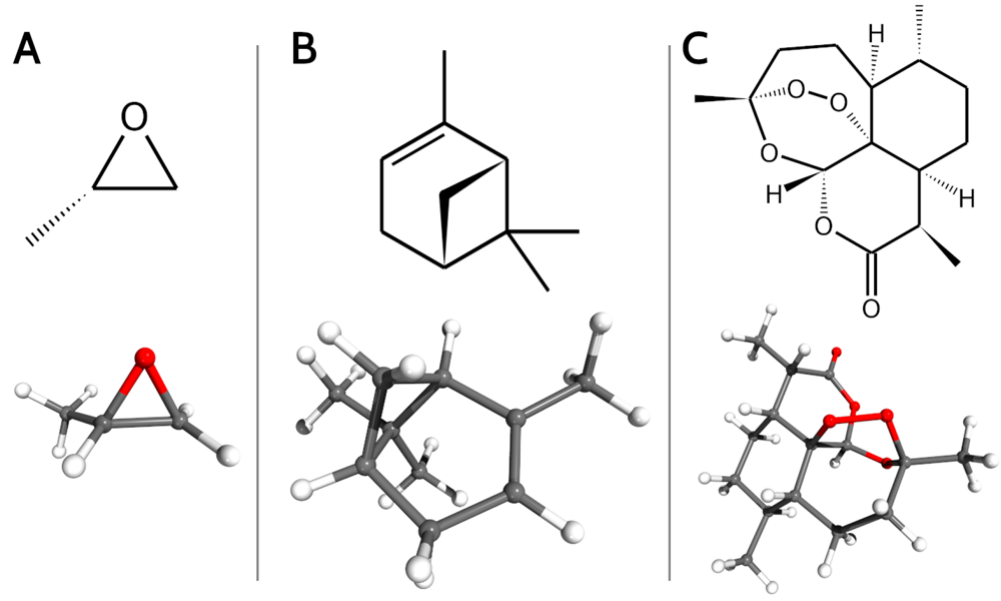
Chemical and 3D structures of (*S*)-2-methyloxirane (A), (1*R*,5*R*)-α-pinene (B), and (1*R*,4*S*,5*R*,8*S*,9*R*,12*S*,13*R*)-1,5,9-trimethyl-11,14,15,16-tetraoxatetracyclo[10.3.1.0^4,13^.0^8,13^]hexadecan-10-one, known as artemisinin (C).

In order to build a robust protocol and assess the performance of criteria to identify the resonances, a bottom-up approach was followed, starting from the vibrational energies of methyloxirane (panel A in Figure 1). As mentioned before, the incidence of resonances on energies is well documented and has been extensively studied, providing a solid ground to devise a systematic protocol to test schemes connected to Fermi resonances. Indeed, since Darling–Dennison resonances do not directly impact resonances, a basic scheme can be chosen. Methyloxirane is sufficiently large (24 modes) and presents interesting cases of resonances. As a standard prototypical chiral molecule, it has been extensively studied, including with state-of-the-art techniques, providing a large database of high-resolution data.^6,18,51,58,82−89^

Because of the lower resolution of the spectrum recorded in the CH-stretching region, roughly between 2900 and 3200 cm^–1^, and thus the higher uncertainty in establishing the correct position of the fundamental bands, this region will be initially excluded. Two sets of experimental data, reported in the literature, will be used as reference. The first one regards data in the gas phase and a low-temperature matrix, collected in ref (88), while the other was extracted from high-resolution Raman scattering spectra (RS) in neat liquid.^51^ To match them, calculations were done in both vacuum and solute with PCM. In the absence of dedicated parameters for methyloxirane, tetrahydrofuran was chosen, a close alternative suggested in refs (51 and 86). In both cases, the harmonic force field was computed at the revDSD-PBEP86-D3(BJ)/jun-cc-pVTZ level (shortened to RDSD) and properties and anharmonic constants, with B3PW91-D3(BJ)/jun-cc-pVTZ (B3PW).

As mentioned above, the initial focus of the study will be the impact of Fermi resonances on the fundamental energies in the fingerprint region. Due to the relatively low density of peaks and the absence of fundamentals close in energy, Darling–Dennison resonances are expected to have little influence. Thus, **R11HRS** and **R22HRS** have been chosen here, using thresholds proposed in the literature.^46,48^ The following parameters were set
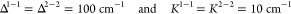


In the gas phase, no DDRs are found in the region of interest. In neat liquid, 1 2–2 DDR involving the overtone of mode 4 and the combination of mode 2 and mode 11 (shortened as “2 + 11”) at about 1540 cm^–1^ and 3 1–1 DDRs involving the fundamentals of modes 2 and 3, 6 and 7, and 8 and 9 are identified. Δ^1–2^ is chosen to be sufficiently large to cover all relevant resonances, so it was kept constant for all schemes, at 200 cm^–1^. Using these parameters as a base, different tests were run to identify Fermi resonances by varying the value of *K*^1–2^ from 0.1 to 1.0 for each scheme. For the sake of comparison, **R12WFRQ** and **R12COEF**, originally designed to identify resonances affecting more specifically the transition moments and thus expected as complements to other criteria centered on energy, were run independently with  set to the same value as *K*^1–2^. To make the analysis meaningful between different thresholds, the GVPT2 energies were systematically used. For each combination of a scheme and a threshold, the fundamental energies of the first 18 modes were compared to the reference values.

**Figure 2:**
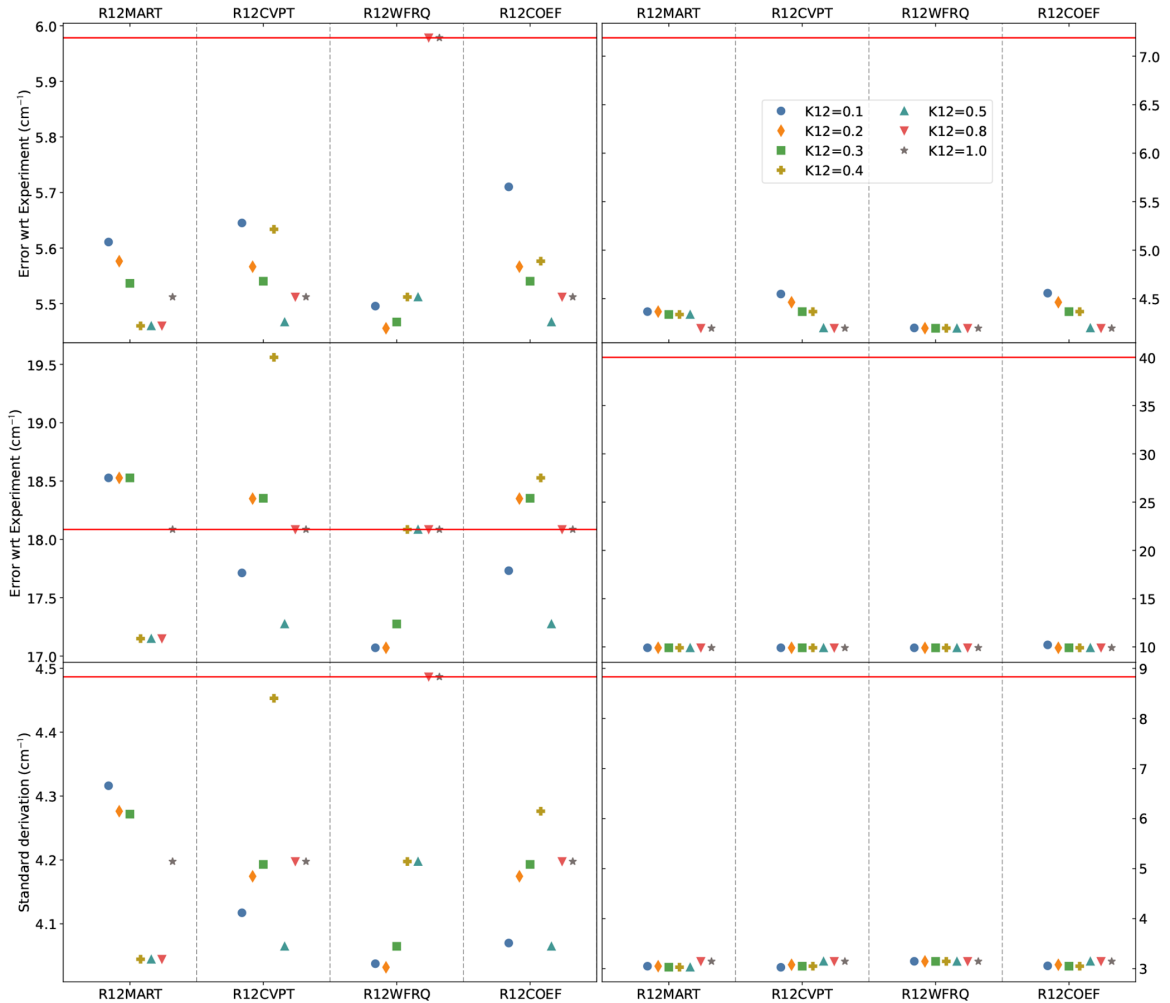
Mean absolute error (MAE, upper panel), maximum unsigned error (|MAX|, center panel), and standard deviation (lower panel) for the fundamental energies below 2800 cm^–1^ for methyloxirane in the gas phase (left panels) and neat liquid (right panels) (18 modes out of 24). Experimental data were taken from refs (51 and 88). To facilitate comparisons, the GVPT2 energies were used, selecting the variational overlap with the highest overlap over the fundamental DVPT2 states. **R12COEF** and **R12WFRQ** were applied as unique tests without any energy-relative test. The red horizontal lines represent the error if no Fermi resonances are treated.

The mean and maximum absolute errors (MAE and |MAX|) as well as the standard deviation are reported in Figure 2, in the upper, central and lower panels, respectively. The gas phase is shown on the left and the neat liquid, on the right. Let us start with the former. The distribution of MAEs is relatively narrow in all cases. **R12CVPT** and **R12COEF** behave in a similar way, which is expected since they rely on similar criteria. The only noticeable difference is for *K*^1–2^ = 0.1 and 0.4. In the first case, the MAE of **R12CVPT** is slightly better than that for **R12COEF**, the latter identifying a resonance between  and , causing the energy of the fundamental to increase by 1 cm^–1^, further from the reference value of 409 cm^–1^ recorded in the gas phase. A similar behavior is observed for *K*^1–2^ = 0.4. This time, **R12COEF** identifies 3 FRs involving . This leads to a correction of the underestimated energy of . Conversely, **R12CVPT** with *K*^1–2^ = 0.4 has the largest error with respect to the experiment among all tested combinations. It is noteworthy that |MAX| is systematically associated with mode 18. Depending on the schemes and thresholds,  can be involved from 0 up to 5 Fermi resonances, causing fluctuations of its energy about the value obtained if they are all ignored. Discarding this mode and considering only the 17 lowest modes (Figure S1) lead to an improvement in the maximum error by about 5.4 cm^–1^, MAE by 0.7, and the standard deviation by 1. Overall, relatively similar patterns are observed across the schemes. Low values for *K*^1–2^ give higher errors, in particular for *K*^1–2^ = 0.1. The only notable exception is for **R12WFRQ**, which produces a low average error and standard deviation at this level. This is due to the fact that the scheme identifies few resonances, even with this low threshold (2 found in the region of interest, compared to 22 for **R12CVPT**, for instance). This low sensitivity has a negative consequence with higher values of *K*^1–2^, the criterion identifying 0 FR for *K*^1–2^ ≥ 0.8. **R12MART** appears very stable in the 0.4–0.8 range, producing among the smallest error with respect to reference data. Similar agreements are reached by **R12CVPT** and **R12COEF** with *K*^1–2^ = 0.5 and **R12WFRQ** with *K*^1–2^ = 0.2. Interestingly, the only difference between **R12MART** and **R12CVPT**/**R12COEF** in these conditions lies in the identification of the Fermi resonances involving , the former predicting a coupling with  and the latter, with . Ignoring this mode in the analysis (Figure S1), the results are the same with these parameters. With *K*^1–2^ = 1.0, none of the schemes predicts a resonance involving the fundamental of mode 18, resulting in a slight worsening of the agreement with the experiment. There again, ignoring mode 18 leads to all schemes except **R12WFRQ** to perform equally for *K*^1–2^ ≥ 0.5.

Let us now consider methyloxirane in neat liquid (right panel of Figure 2). As a first observation, the error in energy caused by ignoring Fermi resonances, relatively mild in the gas phase, becomes large and cannot be disregarded. The maximum error is again related to  and reaches 40 cm^–1^. This difference is to be almost exclusively ascribed to the closer harmonic energies of  and  from 17.5 to 2.6 cm^–1^. This reduction by a factor of nearly 7, coupled to a slight increase of the coupling (the corresponding  constant rising from 33 to 38 cm^–1^), leads for instance to an increase by more than 500 times of the index used in Martin’s test, , from 0.8 to 428 cm^–1^. Because of the intensity of the Fermi resonance, all schemes are able to correctly identify it with the thresholds considered in this study, leading to a significant improvement over the pure VPT2 value. If mode 18 is excluded from the list of fundamentals considered in the calculation of the deviation from reference data (Figure S1), ignoring all resonances still leads to a worse agreement, a result in contrast with the gas phase. This disparity is related to mode 17, whose fundamental is very close in energy to the combination  at the harmonic level, the difference decreasing from 6.1 in the gas phase to 0.4 cm^–1^ in neat liquid, resulting in an intense resonance here too, correctly treated as well. For all schemes, the inclusion or not of mode 18 does not lead to significant changes, the largest error being related to mode 3 (|MAX| ≈ 10 cm^–1^), while  is predicted with an energy very close to the reference data, within 3 cm^–1^. **R12MART** still yields a very good agreement for larger values of *K*^1–2^. In neat liquid, **R12CVPT** and **R12COEF** behave again very similarly with the main difference this time being that **R12COEF** shows a slightly higher error whenever the two diverge, but the difference remains very small, within the error margins from experiment and electronic structure calculations. Nevertheless, all schemes give very good results with a small distribution of errors, which can be explained by the strength of the main Fermi resonances, making their identification easy.

From this first analysis, a few preliminary conclusions can be drawn. The systematic, rather large error on the fundamental energy of mode 18 in gas phase, independently of the chosen scheme, raises some questions about the reliability of the reference value. A possible explanation could be environmental effects caused by the matrix, as already evidenced in the IR and vibrational circular dichroism (VCD) of methyloxirane in the fingerprint region.^18^ The small size of the set of modes considered in this study, together with the relatively weak coupling observed in general, except for a few cases noted in neat liquid, led to narrow variations in the average and absolute errors, preventing the demarcation of specific parameters and criteria to identify Fermi resonances in terms of accuracy. On the basis of their performance, it would seem that low thresholds, as sometimes advocated in the literature,^39,47,67^ may be ill-suited and could lead to some overcorrection.

**Figure 3:**
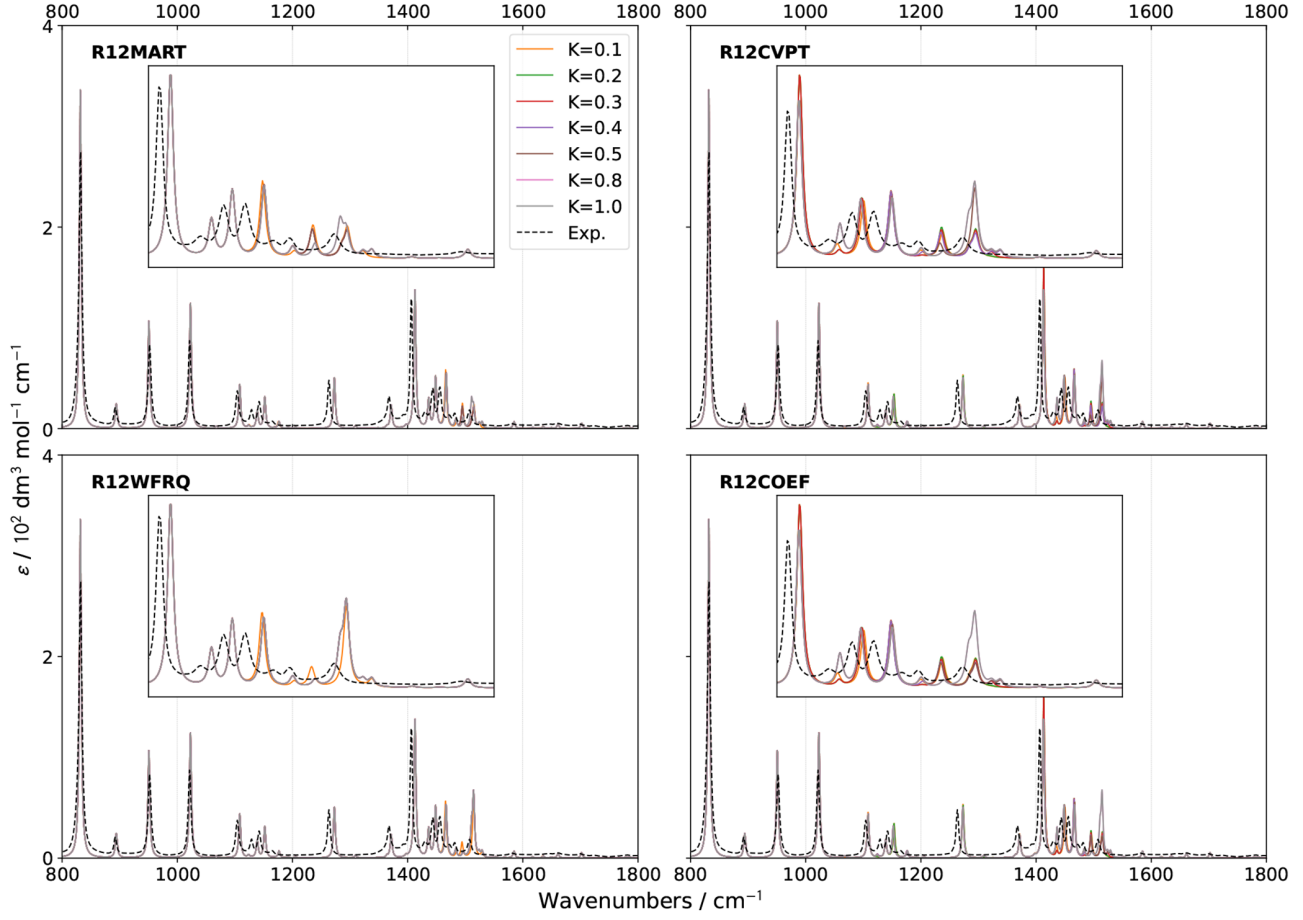
Comparison of theoretical GVPT2 IR spectra of (*S*)-2-methyloxirane in liquid xenon within the fingerprint region using different schemes and thresholds (*K* = *K*^1–2^) for the identification of Fermi resonances. Experimental data (black dashed lines) was taken from ref (18). Lorentzian broadening functions with half-width at half-maximum of 2 cm^–1^ were used to match experiment. The inset shows a zoom of the 1400–1600 cm^–1^ region.

**Figure 4:**
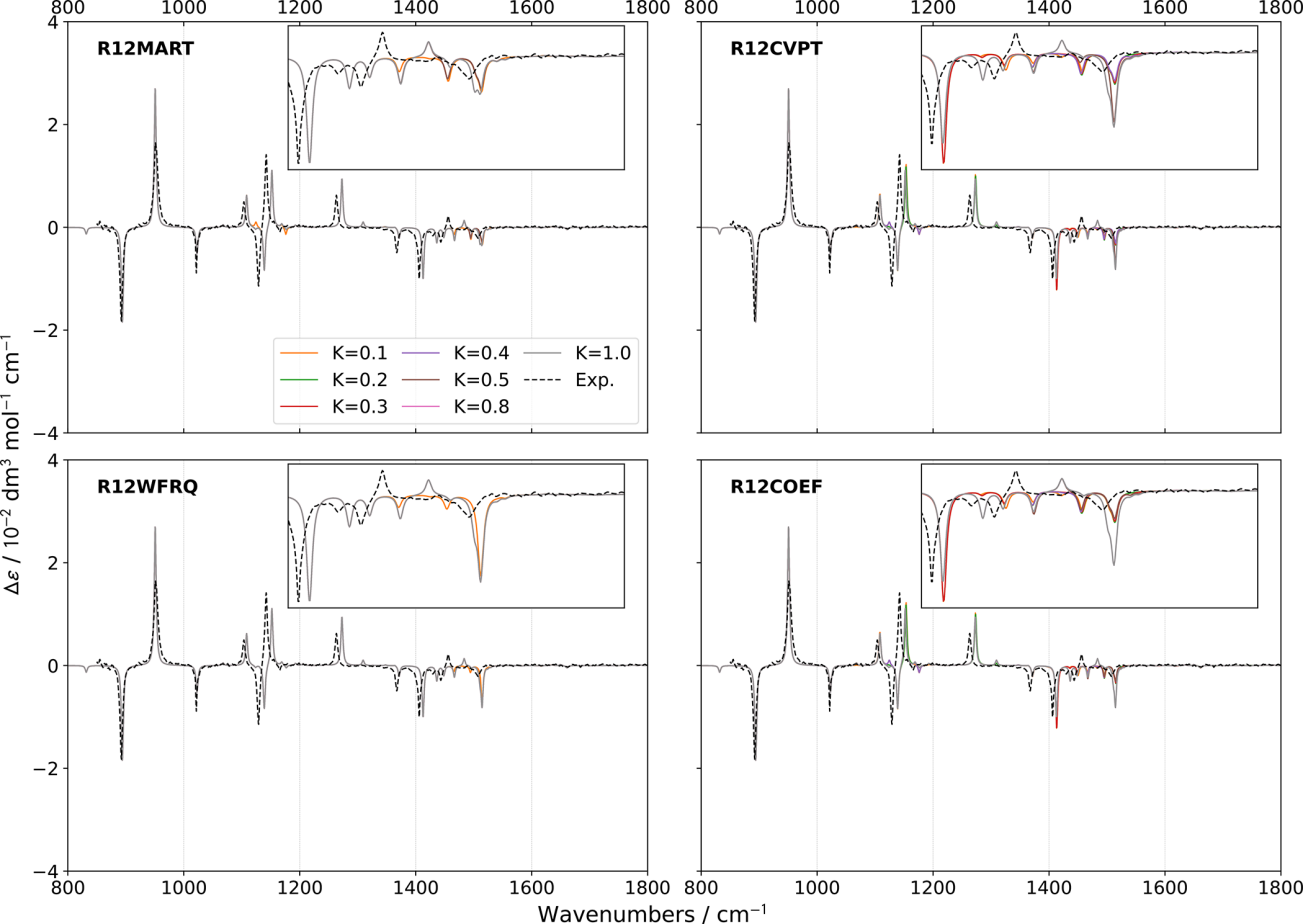
Comparison of theoretical GVPT2 VCD spectra of (*S*)-2-methyloxirane in liquid xenon within the fingerprint region using different schemes and thresholds (*K* = *K*^1–2^) for the identification of Fermi resonances. Experimental data (black dashed lines) was taken from ref (18). Lorentzian broadening functions with half-width at half-maximum of 2 cm^–1^ were used to match experiment. The inset shows a zoom of the 1400–1600 cm^–1^ region.

The energies of fundamentals represent a narrow picture of the spectral information and a limited benchmark to tackle larger molecules, as it becomes increasingly difficult to extract numerical values from experimental spectra without significant processing and the risk of ambiguity in the assignment. Comparing computed and recorded bandshapes thus seems more natural. For such studies to be meaningful, spectra of high quality are necessary, while calculations need to be able to precisely reproduce the conditions met experimentally. To this end, IR and VCD spectra recently registered in liquid xenon (LXe) at low temperature were used as reference, together with Raman and Raman optical activity (ROA) from the neat liquid.^18^ To match the first set of data (liquid xenon), a new batch of calculations were run, simulating the environment by mean of PCM with the same combination of electronic structure calculation methods as before. It should be mentioned that the results in xenon (ϵ = 1.706) are very close to the gas phase, so that the trend observed for the energies are the same in both environments. The IR and VCD spectra in the 800–1800 cm^–1^ region, the so-called fingerprint region, are shown in Figures 3 and 4, respectively.

Overall and as expected from the results of the energies, the theoretical bandshapes are similar to their experimental counterparts for both IR and VCD. Shifting our attention to IR first (Figure 3), little changes are visible in the 800–1400 cm^–1^ region, where few Fermi resonances have been detected, except with very low thresholds, which is in line with their marginal incidence. The region from 1400 to 1600 cm^–1^ is more intricate. The experimental spectrum shows one relatively intense peak, at about 1405 cm^–1^, followed by a series of peaks of lower intensity up to about 1500 cm^–1^, dominated by a doublet around 1460 cm^–1^. A similar pattern is present in VCD. Most peaks are also predicted by computations, albeit with variable relative intensities depending on the criteria and thresholds used for the identification of Fermi resonances. For a better reading, the region is magnified in an inset in Figure 3 (Figure 4 for VCD). It is noteworthy that the band positions show little variations with the thresholds used to identify resonances, while intensities can change greatly, showing that the latter is more sensitive to Fermi resonances and should be considered as a reference to evaluate their impact. The small shift of about 5 cm^–1^ between experiment and calculations above 1100 cm^–1^ cannot be ascribed to resonances and could be due to some calibration errors in the measurements or shortcomings in the electronic structure calculation. Nevertheless, the consistency of this discrepancy makes the connection between experimental and predicted bands straightforward, thus not hindering further analyses.

On these premises, a primary indicator to assess the reliability of the different schemes is the peak at 1507 cm^–1^, measured with a relatively low intensity. The peak, predicted at about 1510 cm^–1^, is systematically overestimated by **R12WFRQ**, even for *K*^1–2^ = 0.1. Similar results are obtained with **R12CVPT** and *K*^1–2^ ≥ 0.5 as well as **R12COEF** and *K*^1–2^ ≥ 0.8. At variance, **R12MART** gives a more acceptable height even for the largest value of *K*^1–2^ considered here. An analysis of the resonances highlight the importance of the resonance between  and  in the corresponding transition moments. An absence of treatment leads to a cancellation of the intensity for the fundamental and an important gain for the overtone. Conversely, if the terms corresponding to this resonance are removed, a peak, albeit weak, becomes visible at about 1500 cm^–1^, which seems consistent with the small band found experimentally at 1482 cm^–1^. As a first conclusion, **R12WFRQ** does not appear to be a good criterion to identify resonances without resorting to very low thresholds and must be combined with others. Further lowering the threshold for *K*^1–2^ in the other schemes unveils a second Fermi resonance involving , this time with the combination . The removal of the resonant terms and their variational correction result in a redistribution of the intensity in favor of the fundamental. The band at 1510 cm^–1^, joint contribution of  and  separated by 4 cm^–1^, is lowered, while the band below 1500 cm^–1^, associated with  grows in height. A visual comparison with the experiment hints at the necessity to account for both resonances, providing an indicative upper bound for an ideal value of *K*^1–2^.

The three more intense bands in the region, predicted at 1410, 1450, and 1465 cm^–1^, are related to the fundamentals of modes 15, 16, and 17, respectively, less affected by resonances. Very low values of *K*^1–2^ (0.2–0.3) for **R12CVPT** and **R12COEF** lead to the identification of a resonance between  and . The removal of all terms affected by the resonance in the transition moments leads to an increase in intensity of the fundamental band and a large decrease up to almost the disappearance of the band at about 1435 cm^–1^ related to that combination. Further lowering the threshold to 0.1 shows a second Fermi resonance involving , this time with . While this barely affects the energy and intensity for the latter, it contributes to a small increase in the intensity of the band at 1435 cm^–1^. The fundamental of mode 17 is also weakly affected by the resonance thresholds. Like mode 15, low thresholds for **R12CVPT** and **R12COEF** predict a resonance between this mode and  with similar consequences; the removal and variational treatment of the resonance leads to the intensity of the combination band to be nullified. With *K*^1–2^ = 0.1, the treatment of a resonance connecting  to  results in a near complete recovery of the intensity. On the basis of these observations, very low thresholds may lead to some error compensation, which may not be systematic, depending on the systems under study, while a choice at about 0.4 or 0.5 may be more robust.

Switching to VCD (Figure 4), many similarities with IR regarding the effect of the treatment of Fermi resonances can be observed. The computed bandshapes below 1400 cm^–1^ are mostly superimposed with the exception of two small bands predicted at about 1124 and 1175 cm^–1^, which appear at low values of *K*^1–2^, except for **R12WFRQ**, which is not sensitive enough. In these conditions,  and , respectively, responsible for the first and second bands, are found in resonance. The correction gives rise to positive and negative bands of low intensity, more visible than in IR. For **R12CVPT** and **R12COEF**, a second resonance involving the combination is identified with the fundamental of mode 11 (second positive peak in the “+, −, +” pattern observed experimentally between 1100 and 1200 cm^–1^). Its treatment leads to a cancellation of the band at 1125 cm^–1^ with a limited effect on the positive fundamental band at about 1150 cm^–1^. While lower thresholds seem to lead to bandshapes closer in agreement with the experiment, the extent of the changes is too small for conclusive observations.

The main region of interest remains between 1400 and 1600 cm^–1^, like in IR. The evolution of the band patterns at 1495 and 1515 cm^–1^ matches what was found for IR, confirming that resonances are connected to the mechanical anharmonicity. Among the notable changes, the band calculated at 1435 cm^–1^ vanishes with **R12CVPT** or **R12COEF** and thresholds below 0.3. Lowering the threshold to 0.1 does not help recover part of its intensity. Furthermore, all simulations fail to reproduce the experimental band pattern, roughly “–, −, −, +, −”, predicted as “–, −, −, −, +, −” with high thresholds or with purely negative bands at lower thresholds. Such a discrepancy may hint at potential shortcomings in the resonance identification process or in the underlying electronic structure calculation.

The simulated RS and ROA spectra of methyloxirane, respectively, in Figures S2 and S3, are mostly invariant with the chosen thresholds and in very good agreement with the experiment in the region below 1600 cm^–1^. This is in line with what was found for the energy and can be explained by the fact that the main Fermi resonances are stronger in neat liquid than in gas/LXe, so that all schemes are able to capture them, even with relatively high thresholds, while lower-intensity resonances have a very weak impact here, even on transition moments.

The CH-stretching region is in some aspects more challenging than the fingerprint, because of the presence of multiple vibrations within a short energy range. In the case of methyloxirane, the 6 CH-stretching vibrations are within 150 cm^–1^ at the harmonic level (RDSD). This makes the region also an interesting case to test the effect of 1–1 Darling–Dennison resonances and the impact of their correction. The reference experimental spectra show broader bands, so a larger broadening was used for the convolution of the theoretical peaks. In practice, the half-widths at half-maximum have been doubled, and the overall bandshape above 3000 cm^–1^ shifted by −22 (IR/VCD) and −18 cm^–1^ (RS/ROA) to match the position of their experimental counterparts and facilitate the comparisons. In the first step, the influence of 1–1 DDR on the intensities was ignored and the parameters for the identification of Darling–Dennison resonances kept unchanged; so, only the impact of the Fermi resonances on the bandshapes is considered, as done in the fingerprint region. The results are shown in Figures S4 to S7 for IR, VCD, RS, and ROA, respectively. It should be mentioned that the experimental VCD spectrum of this region in liquid xenon is not available. A second set of data, recently recorded in carbon tetrachloride up to 9000 cm^–1^, was used in the complement.^89^ Again, a new set of calculations with this solvent was run using Lorentzian functions with half-widths at half-maximum of 10 cm^–1^ as done in the original work. The results are shown in Figures S8 and S9 for IR and VCD, respectively. Contrary to the fingerprint zone, important differences in the bandshape can be observed depending on the thresholds used for the Fermi resonances. The IR spectrum is predicted quite well, especially with low *K*^1–2^, except for a relatively intense peak systematically present at about 3015 cm^–1^, which is not observed experimentally in liquid xenon (Figure S4). With a broader convolution (Figure S8), this peak becomes merged within the band at 3000 cm^–1^. While this leads to a better agreement with the experiment, these results also underline the importance of the resolution in the reference spectra to avoid risks of error compensation. With this observation in mind, let us first discuss the pure theoretical high-resolution VCD spectrum in the region. The bandshape shows important changes as the list of resonances expands, for instance, in the case of **R12COEF** from 2 resonances with *K*^1–2^ = 1.0 to 17 and *K*^1–2^ = 0.1. In the same region, 6 2–2 DDRs and 4 1–1 DDRs are identified with the chosen parameters, causing an important mixing, even for the fundamental bands. As a consequence, a simple assignment in terms of pure fundamentals is not possible anymore at the GVPT2 level.^48^ The resulting redistribution of intensity causes the two bands between 2970 and 3000 cm^–1^ to switch signs from “+, −” with *K*^1–2^ = 1.0 to “–, +”. The exception here remains **R12WFRQ**, which is ill-suited to find the resonances. Depending on the threshold, the bands at 2925 and 3010 cm^–1^ can vanish. A comparison with the experimental spectrum recorded in CCl_4_ (Figure S9) shows that the strong negative band at about 3000 cm^–1^ is an artifact caused by the missing treatment of the resonances. Except for **R12WFRQ**, lowering the threshold for *K*^1–2^ helps recover a more correct intensity. With the larger broadening, a dominant positive band is observed at about 2970 cm^–1^, in disagreement with the experiment, which shows a couplet of relatively low bands. As all thresholds fail to improve the results, a possible cause of this error could be an incomplete treatment of 1–1 Darling–Dennison resonances. Finally, we note that the lowest threshold for **R12COEF** and **R12CVPT** predicts a broad positive feature spanning 2850–2950 cm^–1^, while other parameters predict a negative band, closer to the experiment. Interestingly, while a similar behavior would be expected for ROA with fluctuations of signs depending on the resonance patterns, this is not the case (Figure S7). The signs appear preserved over the range of tested thresholds, but the intensity of the bands is strongly overestimated, in some cases by several orders of magnitude. While not so blatant, a similar trend is observed with RS (Figure S6). **R12COEF** and **R12CVPT** are able to produce closer intensities with the smallest threshold (*K*^1–2^ = 0.1). However, while the first band at 2930 cm^–1^ as well as the higher-energy wing of the broad feature spanning 2950–3040 cm^–1^ are well reproduced in terms of relative positions and intensities, the central element at 2965 cm^–1^ remains significantly overestimated.

**Figure 5:**
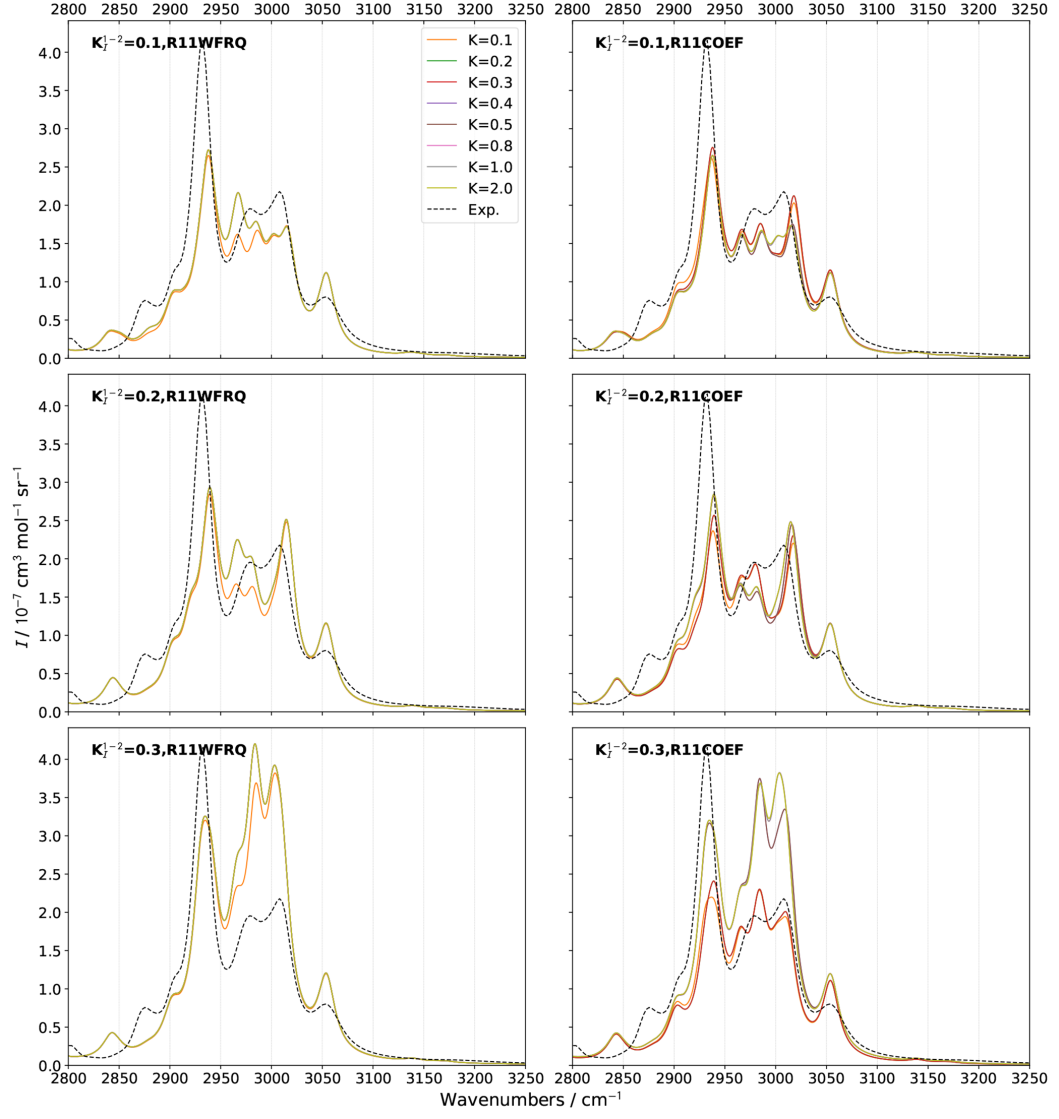
Comparison of theoretical GVPT2 Raman spectra of (*S*)-2-methyloxirane in neat liquid within the CH-stretching region using different schemes and thresholds  for the identification of 1–1 Darling–Dennison resonances. A combination of **R12MART** (*K*^1–2^ = 1.0) and **R12COEF** was used for the Fermi resonances, the second test with the thresholds  (upper panels), 0.2 (middle panels), and 0.3 (lower panels). Experimental data (black dashed lines) was taken from ref (51). Lorentzian broadening functions with half-width at half-maximum of 10 cm^–1^ were used to match the experiment. The computed spectra were shifted by −18 cm^–1^ to match the experiment.

**Figure 6:**
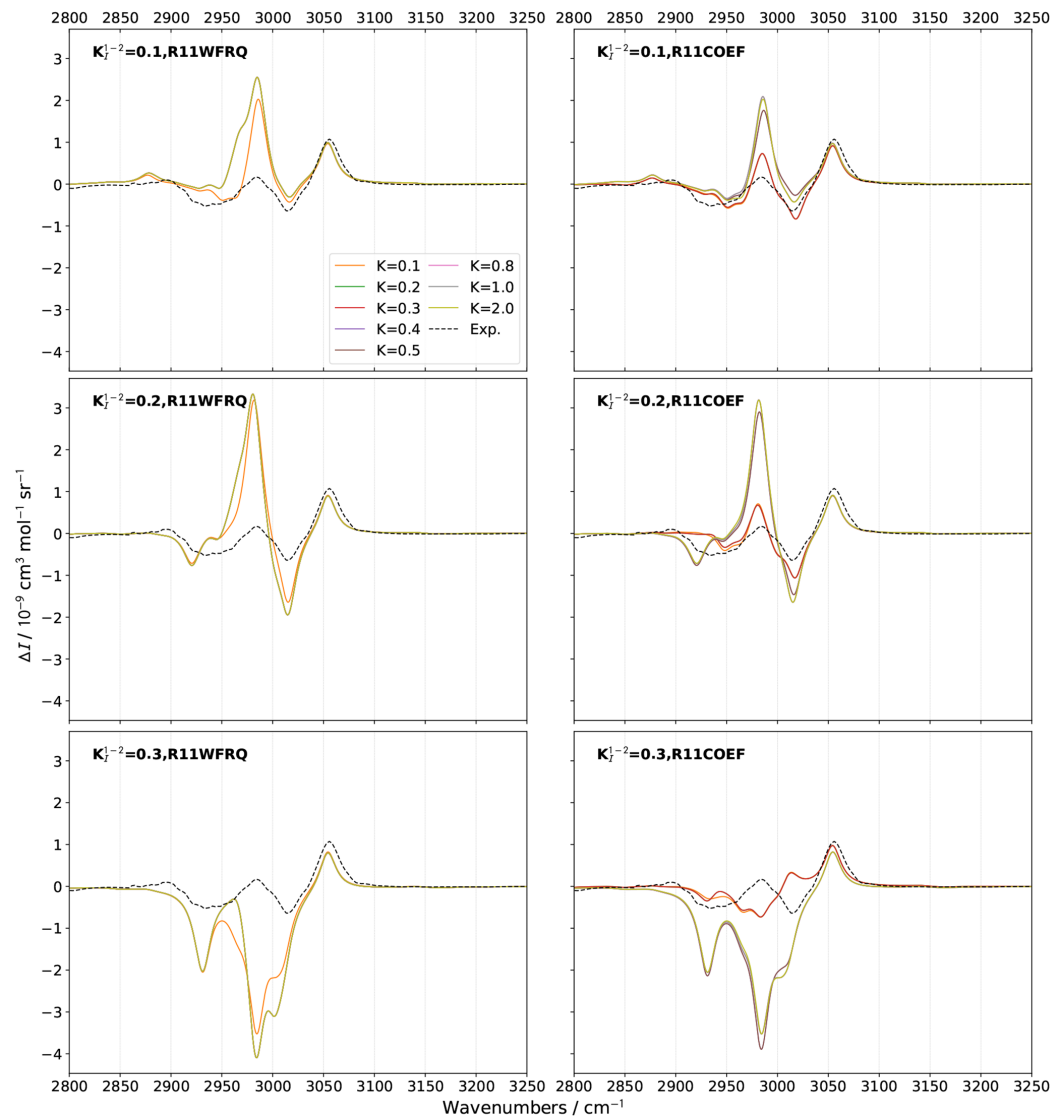
Comparison of theoretical GVPT2 ROA spectra of (*S*)-2-methyloxirane in neat liquid within the CH-stretching region using different schemes and thresholds  for the identification of 1–1 Darling–Dennison resonances. A combination of **R12MART** (*K*^1–2^ = 1.0) and **R12COEF** was used for the Fermi resonances, the second test with the thresholds  (upper panels), 0.2 (middle panels), and 0.3 (lower panels). Experimental data (black dashed lines) was taken from ref (51). Lorentzian broadening functions with half-width at half-maximum of 10 cm^–1^ were used to match experiment. The computed spectra were shifted by −18 cm^–1^ to match the experiment.

The relatively poor results on RS and ROA, but also VCD, underline the necessity for further investigation on the 1–1 Darling–Dennison resonances. The two intensity-specific schemes, **R11WFRQ** and **R11COEF**, were added to the protocol. To keep the discussion focused on a few parameters, only one protocol will be used for the automatic identification of Fermi resonances. On the basis of their performance, a combination of **R12MART** and **R12COEF** is used. *K*^1–2^ was set to 1 (**R12MART**) while different values of  (**R12COEF**), ranging from 0.1 to 0.5, which had shown promising outcomes in the fingerprint region, were considered. The RS and ROA bandshapes in the CH-stretching region for  ranging from 0.1 to 0.3 and different values of  are shown in Figures 5 and 6, respectively, and compared up to 0.5 in Figures S10 and S11.

Because of its sensitivity, ROA represents a good marker to quickly discriminate the less suitable parameters for the identification of resonances. Looking at Figure 6, **R11WFRQ**, which is designed to primarily identify cases of near degeneracy by giving an extra weight to small frequency differences between fundamentals, placed at the denominator, does not provide a significant improvement over **R11HRS**. As a matter of fact, the smallest gap between the harmonic (RDSD) energies of vibrations in LXe beyond 3000 cm^–1^ is 4.6 cm^–1^, which means that new resonances can be identified only with  at least 20 times smaller than the default chosen for *K*^1–1^, 10 cm^–1^. In practice, slight changes are only visible with  but do not significantly affect the bandshape with the sign pattern remaining stable and the band at 2980 cm^–1^ strongly overestimated. The situation differs with **R11COEF**, as clear progressions can be noted when the threshold relative to this scheme is moved. A lower value, , leads to an important drop in intensity, in agreement with experimental measurements. This also highlights the poor performance of the identification scheme used for Fermi resonances with  unable to predict the correct sign pattern in the 2950–3050 cm^–1^ range. In this zone, with *K_I_*^1–1^ ≤ 0.3, an almost perfect mirror image is then obtained. Lower thresholds  give the correct sign sequence, and the application of **R11COEF** coupled with  produces bandshapes relatively close to the experiment. From this study, a combination of the dual criteria **R12MART**/**R12COEF** for Fermi resonances and **R11HRS**/**R11COEF** provides a satisfactory prediction of the spectral bandshape. A similar behavior is observed for the Raman spectrum (Figure 5) with **R11WFRQ** again having no influence. The intensity of the band at 2940 cm^–1^ is constantly underestimated, but the shoulder on its lower-energy side at 2900 cm^–1^ is generally well reproduced. The band below 2900 cm^–1^ seems either missing, predicted as a broad shoulder of low intensity, or red-shifted at about 2850 cm^–1^. Conversely, the relative position and intensity of the higher-energy band at 3050 cm^–1^ is very satisfactory. The relative mode (24) is only weakly coupled to the rest of the system, which explains why the band is mostly unchanged with any value of the threshold to identify resonances. The broader feature spanning 2950–3030 cm^–1^ is a more revealing indicator. With higher values of  (0.3, lower panels), it is predicted with a very high intensity, higher than the band at 2940 cm^–1^, which is experimentally found to be the highest in that region. A more correct description can be recovered with , but the closest shape remains with  and is relatively stable between different values of .

The influence of 1–1 DDR on the IR spectrum (Figures S12 and S14) is small with most noticeable changes in the bandshape related to the Fermi resonances. The lowest threshold for the latter (top panels) seems to again provide the closest match with the experiment, even if discrepancies can be noted below 2950 cm^–1^, a limitation already observed with RS. More differences are observed with CCl_4_ as solvent, but the influence of  remains marginal overall with the exception of . However this threshold does not appear satisfactory, almost constantly giving an excessively broad band around 3000 cm^–1^. Low values of  give a more correct shape, but the positions of the shoulder, experimentally at 2970 cm^–1^, and the band at about 2920 cm^–1^ are offset. A better agreement seems to be with , even if the intensity of the shoulder remains notably underestimated. It is noteworthy that a second shoulder appears at about 3015 cm^–1^ with , consistent with the peak observed at higher resolution in liquid xenon. More changes are visible on VCD (Figures S13 and S15), especially on the relative intensities of the peaks between 2950 and 3030 cm^–1^. In liquid xenon, the first three peaks (“+, −, +”) are related to the fundamentals of modes 21 and 23, while the variational state involved in the fourth one in this range, at 3020 cm^–1^, is related to the first overtone of mode 18. The most impressive transformation regards the peak at 2980 cm^–1^, which is systematically negative with , but in some conditions, positive for higher values of the threshold used for **R12COEF**. The primary reason is the evolution of the DVPT2 electric dipole transition moment and its relative orientation with the magnetic dipole. Indeed, with , the *z* component of  is small (see Table S1). When  is raised to 0.2 with low values of , the magnitude of the *z* component becomes closer to the other components. This causes the angle between the two vectors to be slightly above 90°, hence a change of sign and a near depletion of the band intensity. When the resonances between the fundamentals are reduced, that is, when  is increased, the norm of the electric dipole transition moment increases, while the angle decreases below 90°, flipping the sign. Switching now to the VCD spectrum in CCl_4_ (Figure S15), we observe a good agreement with the experiment by combining the lowest threshold for **R12COEF** and low values of , lower or equal to 0.3. More specifically, the intensity of the band at 2970 cm^–1^ is noticeably reducing, getting close to experiment, and the bandshape between 2900 and 2950 cm^–1^ becomes slightly negative, matching well with the reference. All calculations give a relatively intense positive peak at 3050 cm^–1^, overestimating the low-intensity feature observed. While the comparison study on methyloxirane has provided ample information for the definition of suitable criteria, other tests with different molecules and conditions, which may provide new insights, are necessary to narrow down the most reliable parameters.

As a final note, notable changes are observed with  varying from 0.1 to 0.3, but no particular evolution is observed going further, up to 0.5 (see Figures S10 and S11). Using **R12CVPT** instead of **R12MART**/**R12COEF** (Figures S16 and S17) produces very similar results. No visible differences are observed for ROA, while some small disparities can be observed on the Raman spectra, especially below 2950 cm^–1^. The relative position of the shoulder, experimentally observed at 2900 cm^–1^ is correctly predicted with **R12MART**/**R12COEF** but shifts to lower energies with *K*^1–2^ values larger than 0.3. This is actually due to Martin’s test, confirming that the combination of two tests may be more robust to identify Fermi resonances. Finally, the bandshapes of all four spectra in the fingerprint region, shown in Figures S18 to S21, are unaffected by the addition of the intensity-specific scheme for 1–1 DDR, independently of the chosen threshold, confirming the low impact of the 1–1 Darling–Dennison resonance there.

**Figure 7:**
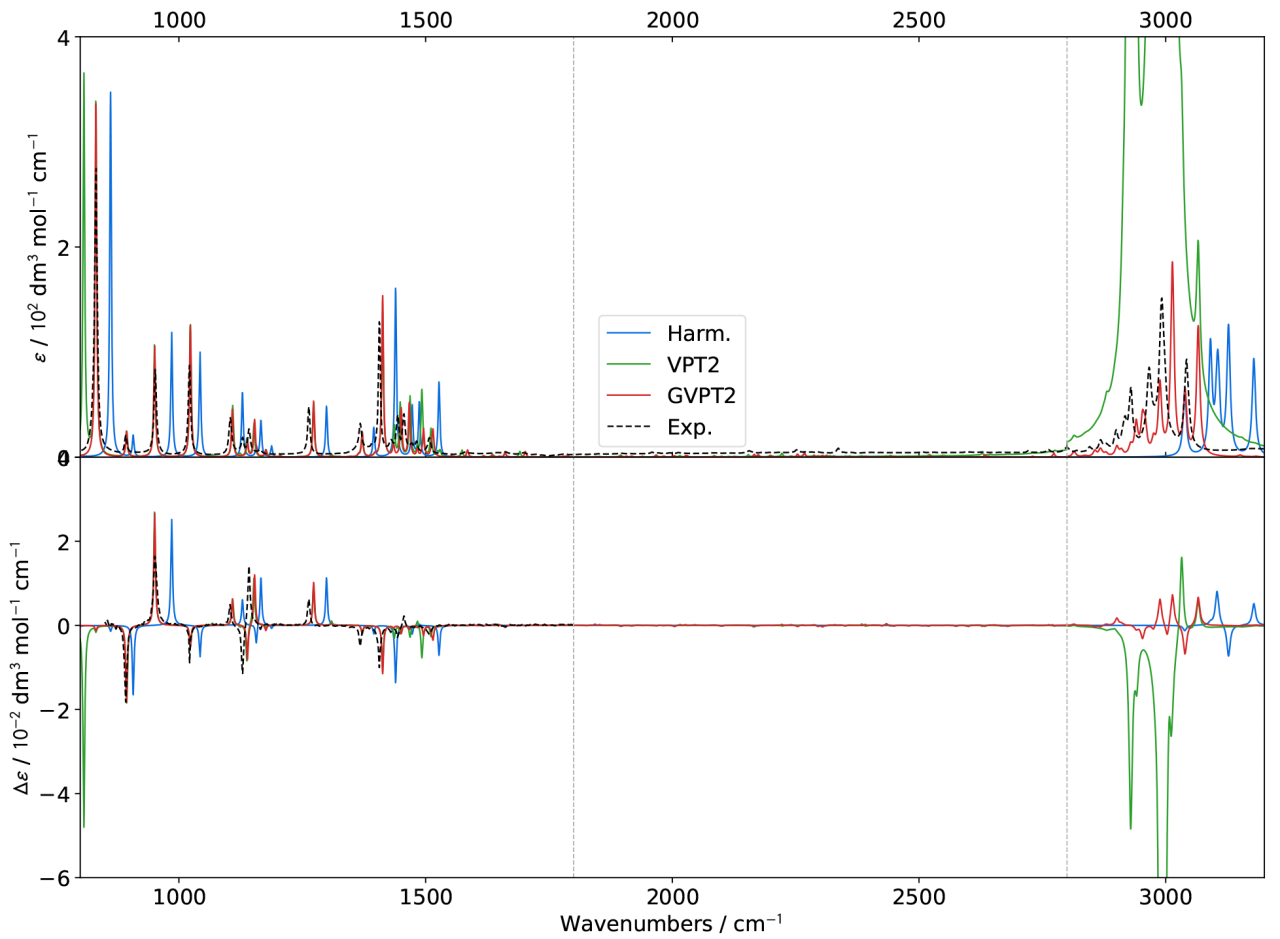
Experimental (black dashed lines),^18^ harmonic (blue), VPT2 (green), and GVPT2 (red) IR (top panel) and VCD (lower panel) spectra of (*S*)-2-methyloxirane in liquid xenon. The automatic procedure used a combination of **R12MART**/**R12COEF** (Δ^1–2^ = 200/*K*^1–2^ = 1.0/*K*_*I*_^1–2^ = 0.1) for FRs, **R11HRS**/**R11COEF** (Δ^1–1^ = 100/*K*^1–1^ = 10/*K*_*I*_^1–1^ = 0.3) for 1–1 DDRs, and **R22HRS** (Δ^2–2^ = 100/*K*^2–2^ = 20) for 2–2 DDRs. Lorentzian broadening functions with half-width at half-maximum of 2 cm^–1^ were used to match the experiment, except in the CH-stretching region, where 4 cm^–1^ was used. The *y* axis was truncated based on the experimental/GVPT2 spectra.

**Figure 8:**
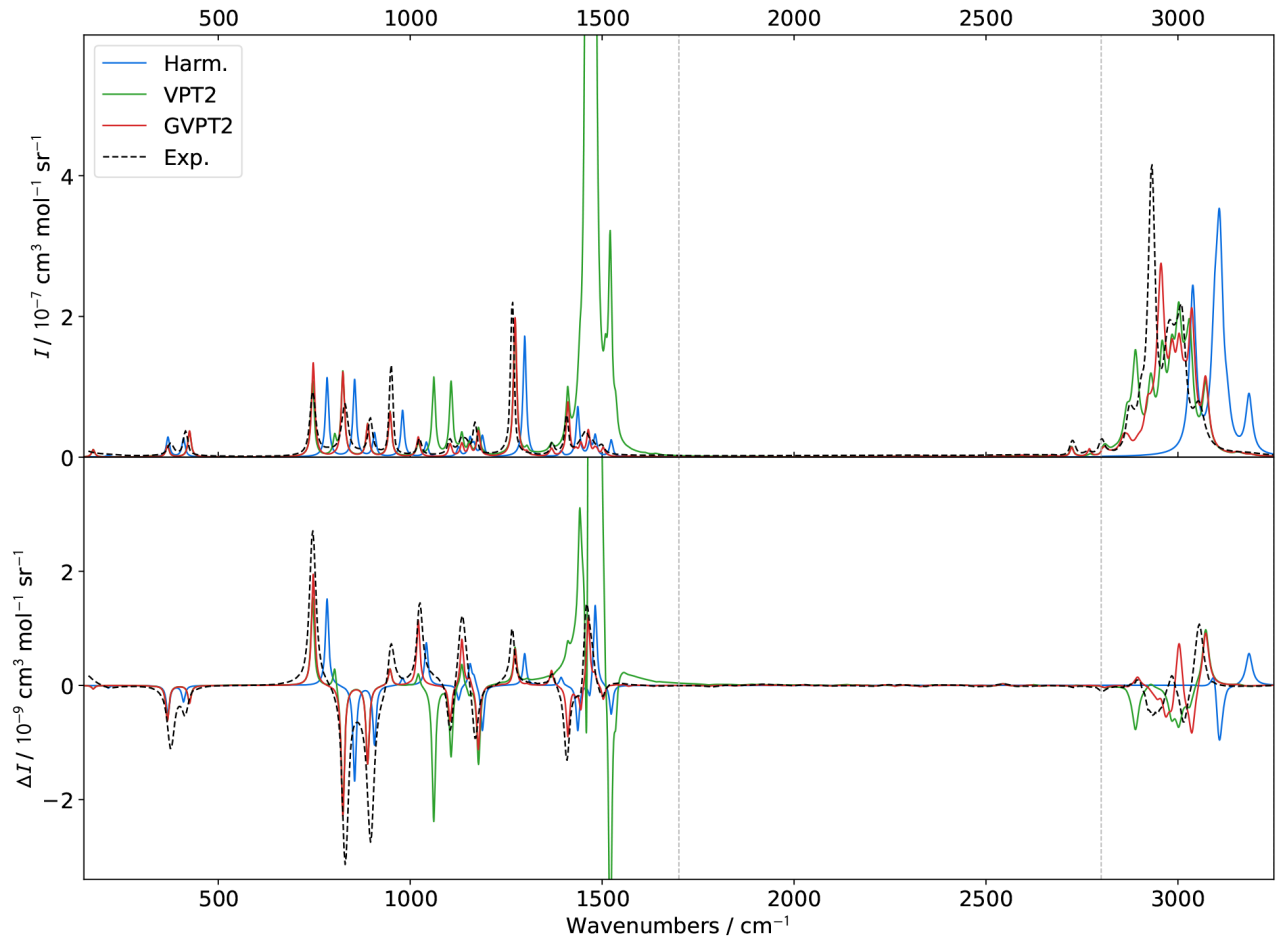
Experimental (black dashed lines),^51^ harmonic (blue), VPT2 (green), and GVPT2 (red) RS (top panel) and ROA (bottom panel) spectra of (*S*)-2-methyloxirane in neat liquid. The automatic procedure used a combination of **R12MART**/**R12COEF** (Δ^1–2^ = 200/*K*^1–2^ = 1.0/*K*_*I*_^1–2^ = 0.1) for FRs, **R11HRS**/**R11COEF** (Δ^1–1^ = 100/*K*^1–1^ = 10/*K*_*I*_^1–1^ = 0.3) for 1–1 DDRs, and **R22HRS** (Δ^2–2^ = 100/*K*^2–2^ = 20) for 2–2 DDRs. Lorentzian broadening functions with half-width at half-maximum of 4 cm^–1^ were used to match the experiment, except in the CH-stretching region, where 10 cm^–1^ was used. The *y* axis was truncated based on the experimental/GVPT2 spectra.

The final, GVPT2 spectra, using a purely automated identification of resonances with optimal parameters, are compared to pure VPT2 (no treatment of resonances), the harmonic approximation, and the experiment in Figure 7 (for IR and VCD) and Figure 8 (for Raman and ROA). While the errors on the VPT2 energies seemed relatively contained, the intensities, especially in the CH-stretching regions, are largely overestimated. In order to see the details, the ordinate axis was truncated. The full spectra can be found in Figures S22 and S23. As expected, the harmonic band positions are blue-shifted, except at lower energies, and most fundamental transitions can still be identified for such a small molecule. The relative intensity of some peaks are incorrectly predicted, especially in the CH-stretching region, where some features recorded experimentally are also clearly missing. Most are well recovered with VPT2, once resonances have been properly identified and treated. It is noteworthy that, even if the CH-stretching energy range is ignored and only the fingerprint region considered, the performance of pure VPT2 remains poor. While VCD and IR appear relatively well predicted, except for an intense peak at 1500 cm^–1^, the computed RS and ROA bandshapes show excessively large features. More specifically, the fundamental transitions related to the strong Fermi resonances discussed before cover the 1480–1520 cm^–1^ range, hiding all other band patterns there. Such a contrast highlights the influence of the properties, but also the environmental effects, in modulating the intensity and the impact of the resonances, confirming the importance of combining multiple spectroscopies and conditions to define robust protocols to systematically identify resonances. The automated procedure described here shows a good stability, being able to provide results of high quality for all spectroscopies.

### Pinene

**Figure 9:**
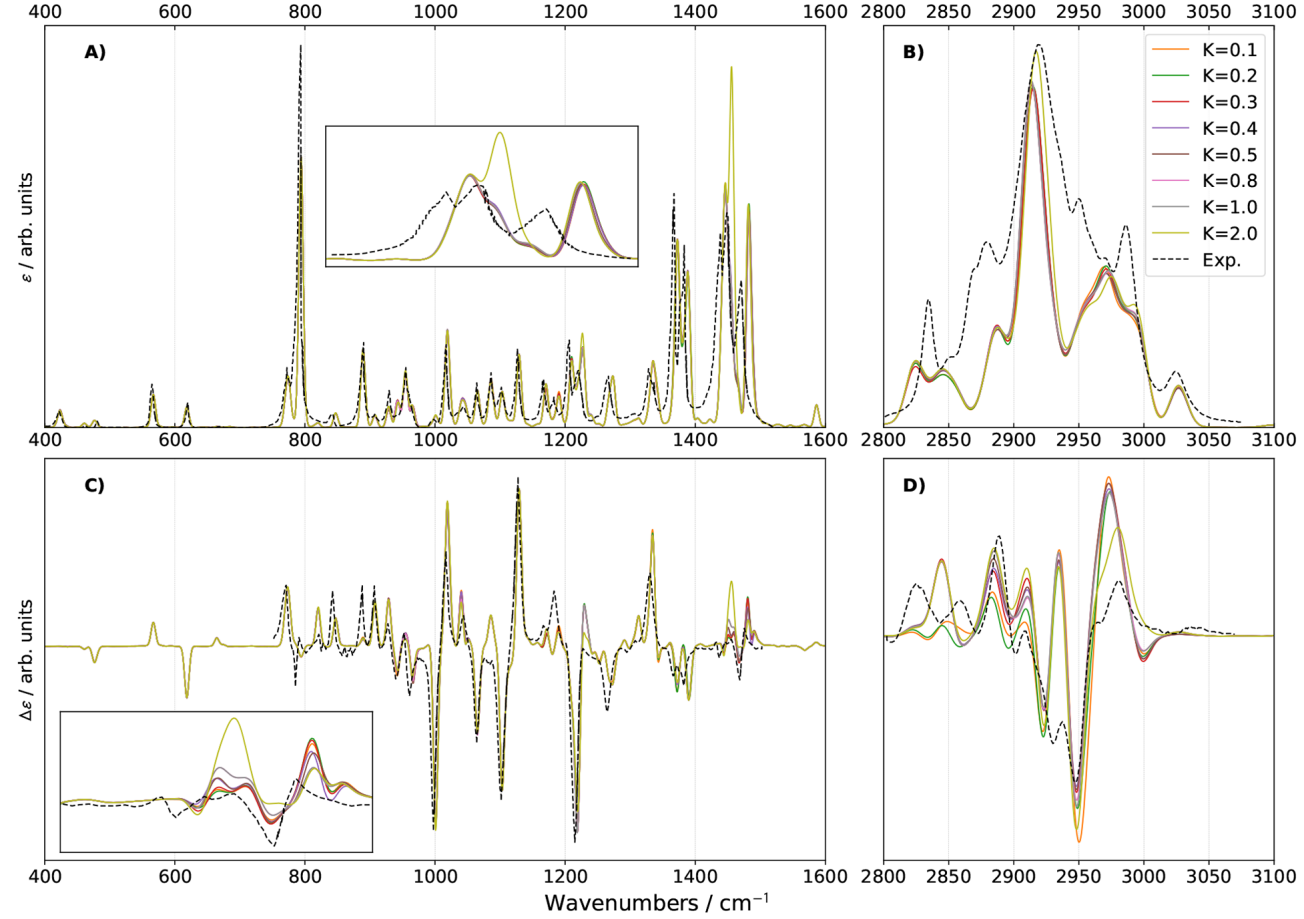
Comparison of the theoretical GVPT2 IR (upper panels) and VCD (lower panels) spectra of (1*R*,5*R*)-α-pinene in CCl_4_ within the fingerprint (left panels) and CH-stretching (right panels) regions using different schemes and thresholds  for the identification of 1–1 Darling–Dennison resonances. Experimental data (black dashed lines) was taken from ref (94). A combination of **R12MART** (*K*^1–2^ = 1.0) and **R12COEF** was used for the Fermi resonances. Gaussian broadening functions with half-width at half-maximum of 4 cm^–1^ (left panels) and 8 cm^–1^ (right panels) were used to match the experiment. The theoretical spectra were shifted by −20 cm^–1^ to match the position of the experimental band in the right panels.

With the experience built on methyloxirane, we move to the IR and VCD spectra of pinene (panel B in Figure 1). With 26 atoms (72 modes) more than twice the size of methyloxirane, it already represents an interesting challenge for anharmonic calculations in terms of computational cost. The rigidity of the structure makes it fully suitable for VPT2 and a good model to study the impact of VPT2 and the problem of resonances. Indeed, there are in theory 2556 possible 1–1 DDRs, more than 184 000 Fermi resonances, and 3 million 2–2 DDRs. A systematic analysis is only conceivable through automated schemes. Like methyloxirane, it is one of the most studied chiral molecules and a standard benchmark for chiroptical instruments, ensuring that reliable data are available for comparison.^84,85,90−97^ For this study, experimental spectra registered in carbon tetrachloride,^94^ which also covered the CH-stretching region, were used as reference. To match the bandshape, the computed peaks were convoluted by mean of Gaussian distribution functions with half-widths at half-maximum of 4 and 8 cm^–1^ in the fingerprint and CH-stretching regions, respectively. To refine the choice of the value of the thresholds for the intensity-related criteria, different values of  and  were tested again. The basis remains the combination validated with methyloxirane, a combination of **R12MART**/**R12COEF** for Fermi resonances, *K*^1–2^ set to 1.0, and **R11HRS**/**R11COEF** (*K*^1–1^ = 10) for 1–1 DDRs. In Figure 9, the threshold for **R12COEF**, , was set to 0.1, a value which gave good results for methyloxirane. The effects on the bandshape with other values are shown in Figures S24 to S27.

Before the results are analyzed further, it should be mentioned that pinene possesses 3 methyl groups, often associated with the presence of large amplitude motions (LAMs). Indeed, the torsional motions are poorly described by polynomial force fields, which can lead to particularly large errors in VPT2. Here, modes 2 (177.9 cm^–1^ at the RDSD level), 4 (205.8), and 5 (225.8) are identified as hindered rotors^98^ with large diagonal fourth derivatives of the energy (respectively, 3715, 2277, and 1907 cm^–1^). These LAMs are customarily removed from VPT2 treatments. To assess their impact and the necessity to discard them, the influence of the  and  thresholds on the bandshapes was analyzed with these modes fully excluded from the VPT2 treatment. In practice, this means that all anharmonic constants involving any of modes 2, 4, or 5 (e.g., and  with *i* and *j* being any normal mode) were set to zero. The comparison between truncated (“NO2,4,5”) and full (“FULL”) systems, in Figures S28 to S31, shows little impact from LAMs and overall better results when considering the whole system. For these reasons, the latter was used. The comparison between **R11WFRQ** and **R11COEF** (Figures S24 to S27) also confirmed the limited performance of the first one and its low sensitivity with the bandshape varying only for important changes in the thresholds, here only when lowering  from 2.0 to 0.1. As a consequence, **R11WFRQ** appears mostly suitable to treat near accidental degeneracies, severely narrowing its applicability.

Starting from IR (upper panels of Figure 9), modifying the value of  produces small changes with the notable exception of the 1400–1500 cm^–1^ zone, magnified in an inset. The agreement in the fingerprint region is overall very good with relative peak intensities in general correctly predicted. The main discrepancy concerns the couplet band above 1400 cm^–1^ and shows how the inclusion of one resonance can lead to significant rearrangements in the intensities and band patterns. Here, using a lower threshold causes modes 51 and 52, both CH_3_ bending motions with harmonic energies within 3 cm^–1^, to be identified as resonant, leading to an important reduction of the dipole strength of the former from 51 to 18 × 10^–40^ esu^2^ cm^2^. While the computations with  do not predict a doublet, the relative intensity of the single band is in better agreement than with , where it is exceedingly large. In the CH-stretching region, the general shape of the band recorded experimentally is confirmed by computations with some of its visible features, especially at 2920 and 3030 cm^–1^, correctly estimated. To facilitate the comparison, the anharmonic bandshape was shifted as a whole by −20 cm^–1^, a value similar to what was used for methyloxirane. The lower wing, below 2900 cm^–1^, is less satisfactorily reproduced. In particular, the band at 2840 cm^–1^ appears as a doublet in the simulations and with larger widths. While the relative position of the peak at 2880 cm^–1^ is correctly estimated, its intensity is only half of the measured one. Higher experimental resolution would be needed to further elucidate the origin of the broader experimental band and help explain the discrepancies between the experiment and theory.

The VCD spectrum shows a higher sensitivity to the resonances with multiple features in the fingerprint regions impacted by the choice of the threshold used for **R11COEF**. The agreement is also overall lower with the relative intensities of some peaks incorrectly predicted in particular between 800 and 950 cm^–1^ and also at 1180 and 1230 cm^–1^. The latter is an interesting case of intensity redistribution and interference caused by the proximity of peaks and the broadening. The intense negative peak at 1220 cm^–1^ arises from the contribution of two GVPT2 states, numbered in the variational sequence 260 and 262 and both balanced mixtures (about 40%) of states  and . The next positive feature with  is primarily due to  at 1227 cm^–1^ with a rotatory strength (*R*) of 5.12 × 10^–44^ esu^2^ cm^2^ (the magnitude and unit will be dropped in the following for clarity). In the convoluted spectrum, this band is partially affected by the intense band at 1220 cm^–1^ but even more impacted by a very close positive transition to the combination  at 1228 cm^–1^ (*R* = −2.4). With lower values of , a resonance is identified between  and . While the impact on the DVPT2 transition moments is relatively limited, the new coupling induces a change in the nature of some variational states, in particular those at 1227–1228 cm^–1^. In practice, the GVPT2 rotatory strengths associated with states 260 and 262 are barely affected (*R* going from −6.6 to −7.8 and −8.2 to −7.4, respectively). On the other end, the transition at 1227 cm^–1^ shrinks in intensity (*R* = 1.6), while the rotatory strength of the one at 1228 cm^–1^, originally negative, becomes positive (from −2.4 to 2.1). The reason for these changes can be explained by the transformation of the GVPT2 states. Without the 1–1 resonance, they are very close to the DVPT2 ones, with the former being predominantly defined as  (>80%) and the latter by  (75%). The inclusion of a coupling between  and  leads to a mixture of these states after the variational correction. The first state, numbered as 265, becomes a 50/40 combination of the fundamental and binary combination, while the second state, 267 is dominated by  and , respectively, with 45% and 20% of overlap. As a result, the destructive effect observed with a high value of  is replaced by a constructive action of the two transitions, leading to a rise in intensity of the resulting band.

Like IR, the 1400–1500 cm^–1^ zone shows a higher dependence than the rest of the fingerprint region on the threshold used to identify 1–1 DDR. To make those changes easier to see, a zoom is included as an inset in the lower left panel of Figure 9. Notwithstanding a general shift of about 5 cm^–1^, values of  below 0.3 lead to a very good agreement with the experiment. The relative intensities of the peaks are correctly predicted with the exception of the one at 1480 cm^–1^, where the band is notably overestimated. While  performs the best here, the rest is far less satisfactory with the negative band at 1470 cm^–1^ missing and an intense peak predicted at 1450 cm^–1^ that is closer to a low doublet in the experiment. These observations cast a doubt on the reliability of the results at 1480 cm^–1^ and a possible effect of error compensation.

Moving to higher energies, the CH-stretching region (lower right panel of Figure 9) shows a high sensitivity of VCD to couplings between CH-stretching modes. The region can be roughly divided in 3 zones depending on the performance of computations, 2800–2870 cm^–1^ (A), 2870–2920 cm^–1^ (B), and 2920–3020 cm^–1^ (C). The experimental spectra in (A) are characterized by two bands, while the theory shows a single band. It is noteworthy that the exact opposite happened for IR. Most thresholds produce the same result in this zone, except the two lowest values, which lead to a near cancellation of the band. While none are close to the experiment,  seems to produce a more reliable picture. The agreement appears better in (B) with two peaks obtained for any value of , as observed experimentally. A peculiarity of this part is that almost each threshold produces a distinct bandshape with the only exception being  and , which are superimposed. The explanation comes from the complex networks of resonances in the CH-stretching region. As a matter of fact, zone (B) contains 22 transitions, all connected through a single polyad involving 51 states, among which 15 are fundamentals. The presence or absence of couplings between these fundamentals leads to redistributions of the intensities and changes in the definition of the variational states. All methods overestimate the relative intensity of the second peak above 2900 cm^–1^, but here again, the lowest thresholds also significantly underestimate the first peak. Finally, the performance of GVPT2 in zone (C) is more complicated to assess. The couplet of negative peaks at 2925–2950 cm^–1^ is correctly predicted, and their relative intensity is quite satisfactory with  being the closest to experiment. However, a strong positive peak is consistently found between the two negative ones, at about 2940 cm^–1^. The chosen broadening (full-width at half-maximum of 16 cm^–1^) seems reasonable with respect to the overall bandshape, hinting to the potential lack of resolution in the experiment. In these conditions, the relative intensities of the two negative peaks could be incorrect. Above 2950 cm^–1^, there is little variation in the bandshape for  with an overestimation of the positive band, followed by a negative peak at 3000 cm^–1^ not found experimentally. On the contrary, reducing the coupling in the region  seems to lead to a shape closer to the experiment with the only divergence on the intensity of the positive band. Despite some notable variations in the bandshape depending on the threshold, the identification of an outperforming criterion is hindered by the limited resolution of the experiment. While further studies could help refine this choice, values of  in the range of 0.3–0.5 appear to provide the steadiest performance with the most accurate prediction of the spectral bandshape.

**Figure 10:**
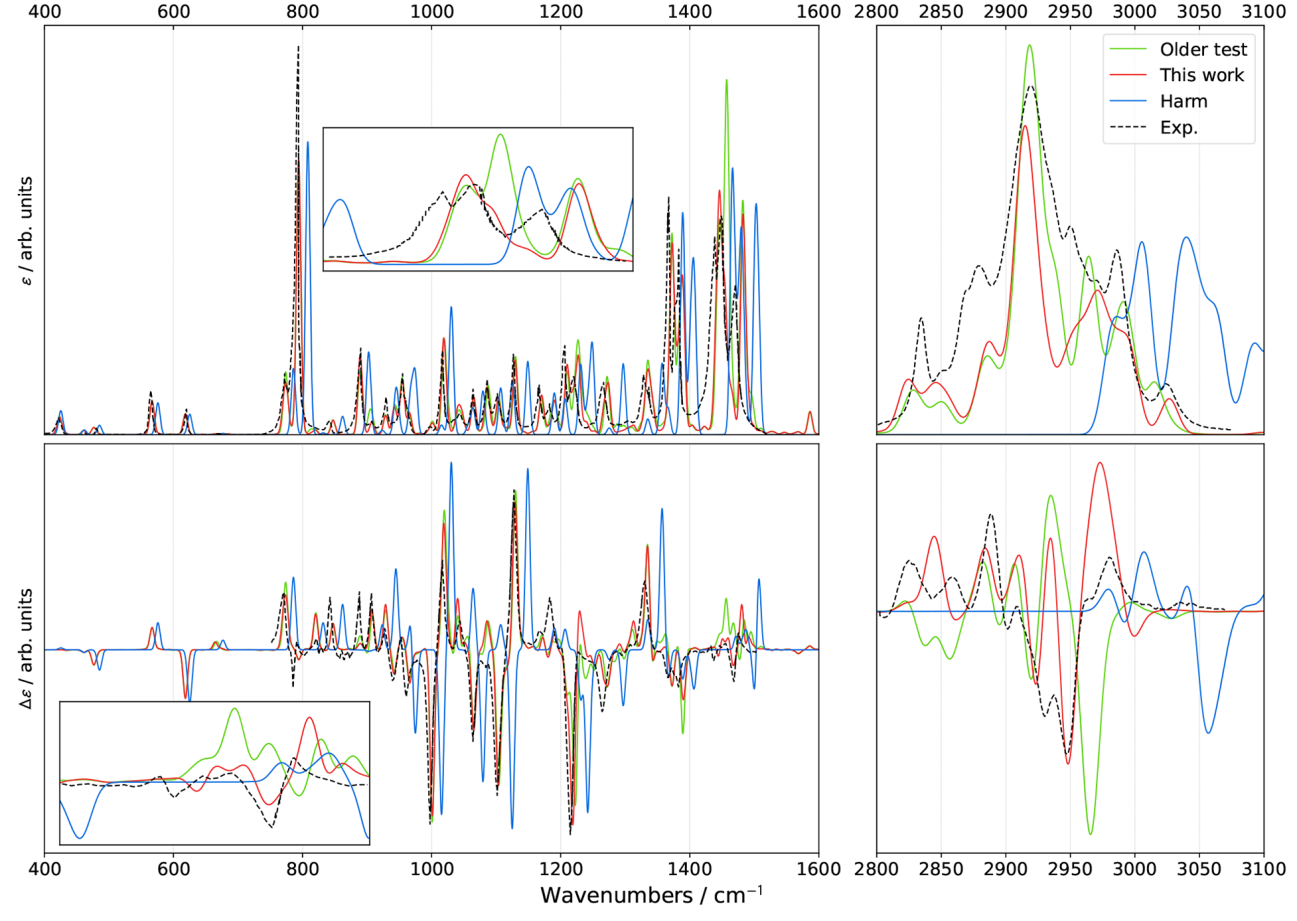
Experimental (black dashed lines)^94^ and GVPT2 IR (upper panels) and VCD (lower panels) spectra of (1*R*,5*R*)-α-pinene in CCl_4_ within the fingerprint (left panels) and CH-stretching (right panels) regions. The GVPT2 spectra were obtained using the protocol described in ref (48) (“Older test”) and with the new protocol presented here (“This work”; see text for details). Gaussian broadening functions with half-width at half-maximum of 4 cm^–1^ (left panels) and 8 cm^–1^ (right panels) were used to match experiment. The theoretical spectra were shifted by −20 cm^–1^ to match the position of the experimental band in the right panels.

Higher values of  (Figures S24 to S27) have rather minor effects on the IR bandshape. The most visible impact is the splitting of the broad cluster spanning 2940–3010 cm^–1^ with *K*^1–2^ ≥ 0.3 (Figure S25) into two distinct bands. The differences are clearer on VCD (Figures S26 and S27), including the fingerprint region (Figure S26). The negative band at 1470 cm^–1^ is an interesting marker. While it was in some conditions missing or underestimated with , the sign is systematically wrong with higher thresholds. A similar situation happens in the CH-stretching region. The 2800–2870 cm^–1^ zone is still poorly reproduced. With  and , a doublet is actually predicted but is a mirror of the experiment. The rest of the bandshape is also unsatisfactory with little overlap between the theory and experiment.  has the worst agreement with a nearly flat band predicted above 2870 cm^–1^ for  and mostly wrong signs with higher thresholds for the 1–1 DDR.  fares better but is still unable to recover the most intense feature in the CH-stretching region between 2920 and 2950 cm^–1^. This comparison confirms the choice of . Undoubtedly, a higher resolution of the reference spectrum could provide more details and further refines the protocol. Nevertheless, the high sensitivity of the VCD signal has permitted an identification of reliable parameters and confirmed the quality of the proposed protocol and associated criteria. The final spectra with optimized parameters are shown in Figure 10. A marked improvement in the prediction of the bandshape compared to the experiment over the previously proposed protocol^46,48^ can be noted starting from 1350 cm^–1^.

### Artemisinin

**Figure 11:**
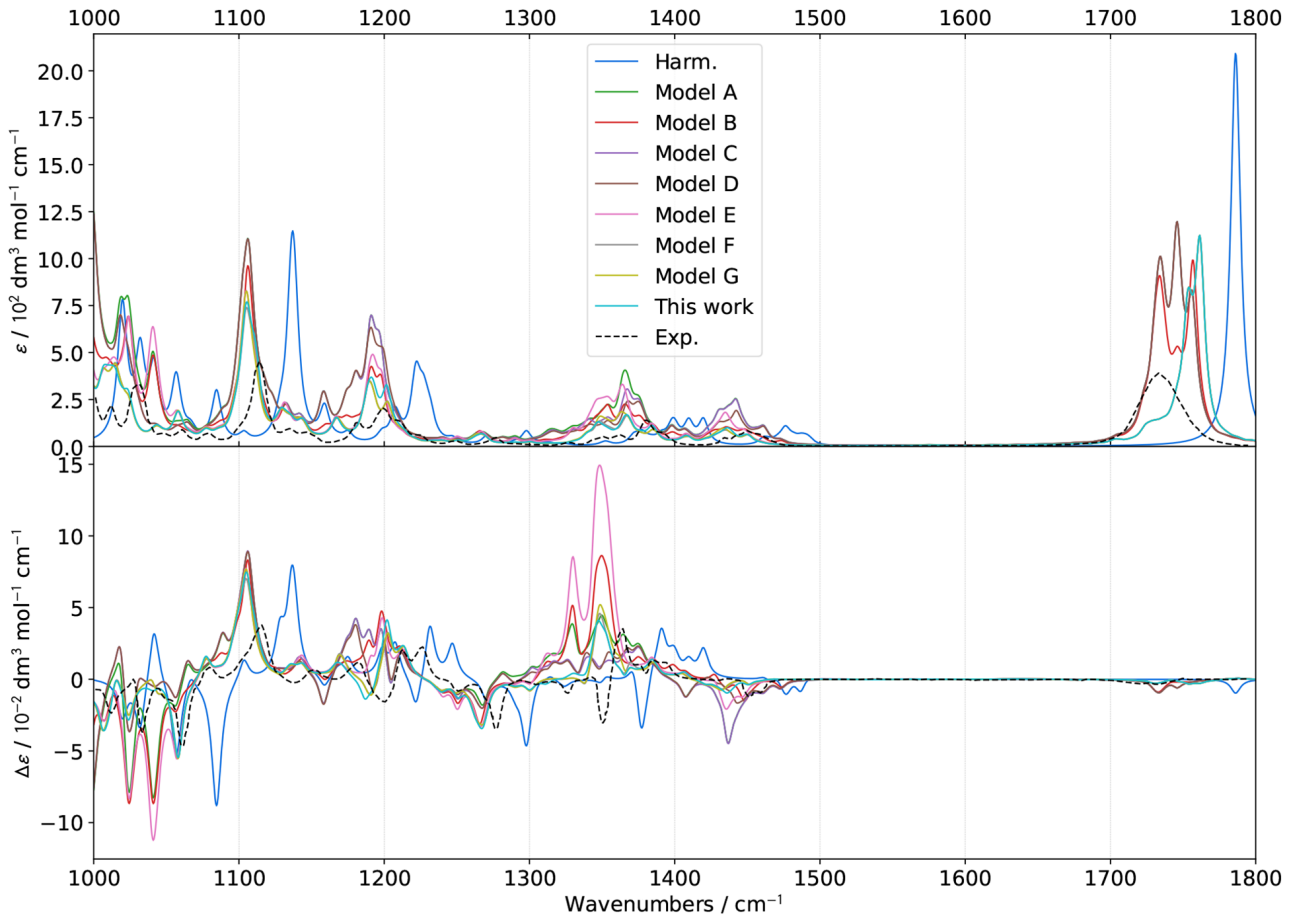
Experimental (black dashed lines)^11^ and GVPT2 IR (upper panel) and VCD (lower panel) spectra of artemisinin in chloroform. The models are described in Table 2. Lorentzian broadening functions with half-width at half-maximum of 4 cm^–1^ were used to match the experiment.

As a final test for the automated procedure, we consider the IR and VCD spectra of artemisinin (panel C in Figure 1), whose fingerprint-region IR and VCD spectra have been recently published as an example of the power of VCD and ROA to obtain unambiguous assignments of the absolute configuration.^11^ With 42 atoms (120 normal modes) and seven chiral centers, the molecule represents a complex challenge, even for the calculation of the harmonic frequencies with double-hybrid functionals and triple ζ-quality basis sets used previously as reference. For this study, a more affordable level of theory was chosen, B3PW91-D3(BJ) in conjunction with the SNSD basis set, a basis set built upon 6-31G(d,p) in which the diffuse functions of the aug-cc-pVDZ basis set and, for non-hydrogen atoms, a very tight *s* function have been added to provide a better description of spectroscopic observables in a cost-effective way.^99^ Different criteria based on parameters proposed in the literature (see Table 2) were applied to simulate the spectra, shown in Figures 11 and S32.

**Table 2:** Description of the Criteria Used to Automatically Identify the Resonances in Figure 11^a^

	Fermi				1–1 Darling–Dennison			
	schemes		thresholds		schemes		thresholds	
model	energy	intensity	*K*^1–2^	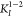	energy	intensity	*K*^1–1^	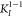
A	**R12MART**		1.0		**R11RHS**		10.0	
B	**R12MART**		0.1		**R11RHS**		10.0	
C	**R12MART**		1.0		**R11RHS**	**R11WFRQ**	10.0	1.0
D	**R12MART**		1.0		**R11RHS**	**R11WFRQ**	10.0	0.1
E	**R12CVPT**		0.1		**R11RHS**		10.0	
F	**R12CVPT**		0.1		**R11RHS**		0.1	
G	**R12CVPT**		0.1		**R11RHS**		0.3	
this work	**R12MART**	**R12COEF**	1.0	0.1	**R11RHS**	**R11COEF**	10.0	0.3

a For all models, the thresholds on the energy were set to Δ^1–2^ = 200 and Δ^1–1^ = 100. 2-2 Darling–Dennison resonances were identified by means of **R22HRS** with the parameters: Δ^2–2^ = 100 and *K*^2–2^ = 20.

First of all, we note a clear decrease of the overall quality of the simulations compared to methyloxirane and pinene. This is consistent with the lower quality of the underlying electronic structure calculation. Using pure VPT2, without treatment of the resonances, results in huge intensities due to the near degeneracies of some states involved in resonances. For instance, 19 Fermi resonances involve states within 0.1 cm^–1^, reaching even for one case 10^–2^ cm^–1^. Let us look more into details at the models (Table 2). Models A, B, E, and F are rooted on the energy. Models A and B rely on Martin’s test for the identification of Fermi resonances with 2 different thresholds, 1.0 (A) and 0.1 (B), while the test on 1–1 DDR is constant (**R11HRS**, *K*^1–1^ = 10.0). The models show especially large discrepancies compared to other models in the 1300–1400 cm^–1^ region as well as below 1100 cm^–1^. In both IR and VCD spectra, this translates into strongly overestimated bands, for instance, between 1100 and 1200 cm^–1^ in the IR spectrum. The prediction of the VCD pattern over 1300–1400 cm^–1^ is far from the measurements, both qualitatively and quantitatively. While the experiment shows an alternation of sign (“–, −, +, +”), calculations produce either a broad positive band spanning the whole range at high threshold (model A) or 2 very intense bands with lower values (model B).

Replacing Martin’s test with the one proposed in ref (67) with *K*^1–2^ = 0.1 (model E) actually nearly systematically worsens the quality of the calculations with an even larger overestimation of some features. This is particularly evident in the VCD spectrum between 1300 and 1400 cm^–1^. Lowering the threshold for **R11HRS** to *K*^1–1^ = 0.1 (model F) leads to a bandshape closer to the experiment and to the protocol proposed in this work. Some small differences can be perceived. However, because of the limited resolution of the experiment, associated with the lack of information on the CH-stretching region, where the treatment of 1–1 DDRs is expected to be crucial, it is not possible to confirm the reliability of such a threshold. Nevertheless, we can note that the test is not tailored for transition moments but instead related to the energy, so that low thresholds may be necessary as compensation, rising the risk of an overcorrection. Increasing *K*^1–1^ to 0.3 (model G), the same threshold as the one used for **R11COEF** in our protocol, induces negligible changes with respect to 0.1.

Finally, the test previously used and relying on **R11WFRQ** is shown as model C^48^ and model D . Contrary to before, the scheme has an influence on the bandshape even with a higher threshold (model C), which can be explained by the existence of very close harmonic states which satisfy the conditions of near-degeneracy sought with this scheme. A lower intensity of the IR and VCD spectra is observed in the 1300–1400 cm^–1^ region. However, the VCD bandshape remains in poor agreement with the experiment. Lowering the threshold on **R11WFRQ** again improves the results with the strong negative VCD peak at about 1440 cm^–1^ significantly reduced, closer in intensity to the small negative group measured experimentally at about 1460 cm^–1^. Overall, the computed spectral bandshape remains distant from its experimental counterpart.

The new test in general offers a satisfactory agreement below 1300 cm^–1^ in terms of band intensities, but the error on the band positions is significant with an almost systematic red-shift compared to the experiments. This behavior is observed with all models, hinting at a problem rooted in the electronic structure calculation itself. Another common trend between the models is in regard to the negative band observed experimentally at about 1350 cm^–1^, missing in anharmonic corrections, while present at the harmonic level. This could again be related to the electronic calculations and more specifically to the harmonic frequencies at the B3PW91-D3(BJ)/SNSD level. Indeed, small variations in the harmonic energies can impact the magnitude of the perturbative terms, which often involve frequency differences at the denominator, but also influence the definition of the variational states and the intensity redistribution. This kind of discrepancy can also signal potential risks of error compensation at a purely harmonic level, which should be carefully assessed to confirm the validity of the overall computational protocol. A higher level of theory for the harmonic level could help improve the overall quality of the GVPT2 calculations. Still, the new protocol seems capable of identifying all important resonances, providing reliable results even in complex conditions of resonances. This will pave the way to a more detailed analysis of the physicochemical properties of artemisinin and its bioactivity, a study deferred to a future work.

## Conclusions

A new, automated protocol has been developed and implemented, which takes into account the specificities of energy and intensity calculations. It relies on two sets of tests for Fermi and 1–1 Darling–Dennison resonances, one designed for energies, which are less sensitive to weak resonances, and one for intensities. For Fermi resonances, the standard test by Martin et al.^43^ is complemented by the new one based on the wave function coefficients. For 1–1 DDRs, the new **R11COEF** scheme is added to the common test based on the magnitude of the Darling–Dennison term. The gain over previously proposed strategies, especially for Darling–Dennison resonances, is undeniable. The additional cost induced by the extra test can be mitigated, since it relies on perturbative wave function coefficients, which are also necessary to build effective vibrations representing the VPT2 states. This work also paves the way to robust VPT2 protocols including 3-quanta transitions.

Using methyloxirane as a primary reference, thanks to the availability of high-quality experimental data, it was shown that the development and validation of strategies to automatically identify resonances based only on the energy could be incomplete, as the latter shows little variation on weak couplings. On the other hand, transition moments and intensities are far more sensitive, representing a better benchmark. Among them, chiroptical spectroscopies can provide fine details even on the most subtle interplays between resonances. However, they are also more challenging for the experiment, due to the weakness of the signal, and theory, as multiple properties are involved, often unveiling shortcomings in the chosen electronic structure calculation method. Another noteworthy aspect highlighted by the study is that even small changes in the solvent effects can have important consequences on the impact of resonances, especially by modulating the harmonic energy difference present at the denominator. Hence, depending on the conditions and system size, resonances may be more or less obvious to identify. This aspect is further complicated by the contribution of the properties to the transition moment, which can modulate the effect of the mechanical anharmonicity and thus weaken or enhance the impact of resonances. In this respect, the present study provides a thorough and methodical picture on resonances and establishes robust criteria to automatically identify them. In practice, we found that using the combination **R12MART**/**R12COEF** with the parameters “Δ^1–2^ = 200/*K*^1–2^ = 1.0/*K*_*I*_^1–2^ = 0.1” and **R11HRS**/**R11COEF** with “Δ^1–1^ = 100/*K*^1–1^ = 10/*K*_*I*_^1–1^ = 0.3” overall gave the best results.

The application of this new protocol on larger systems, pinene and artemisinin, shows encouraging results toward the design of black-box procedures to facilitate the use of VPT2 on large systems, even by nonspecialists. This study also highlighted the necessity of more properties available from high-level electronic structure calculation methods, including chiroptical properties, and the importance of high-quality experiments to identify shortcomings and paths of improvements in current theoretical methodologies. The availability of a robust protocol to find and treat resonances in VPT2 is also an important step in precisely establishing the limitations of VPT2 in the description of semirigid molecular systems and to devise *ad hoc* protocols combining VPT2 with suitable methods to treat large amplitude motions, paving the way for the accurate characterization of biomolecules and other semiflexible systems of technological interest.

## Supporting Information Available

An explicit definition of the VPT2 transition moments with respect to the harmonic wave functions, a discussion of the treatment of resonances within Darling–Dennison terms, and the equations for 3-quanta transitions; additional figures for the analysis of the impact of the thresholds on the energies and intensities on methyloxirane, pinene, and artemisinin (PDF)
