# Supplementary material for: An Effective and Automated Processing of Resonances in Vibrational Perturbation Theory Applied to Spectroscopy
Source: J Phys Chem A. 2022 Nov 30;126(49):9276–302. doi: 10.1021/acs.jpca.2c06460 (PMC9761684; doi:10.1021/acs.jpca.2c06460)
Supplement: Supplementary file 1 — jp2c06460_si_001.pdf [file jp2c06460_si_001.pdf]

Supporting Information: An Effective and Automated Processing of Resonances in Vibrational Perturbation Theory Applied to Spectroscopy

Qin Yang*,†,‡ and Julien Bloino*,†

†*Faculty of Science, Scuola Normale Superiore, Piazza dei Cavalieri 7, I-56126 Pisa, Italy*

‡*Institute of Organic Chemistry and Biochemistry, Czech Academy of Sciences, 16610 Prague, Czech Republic*

E-mail: qin.yang@uochb.cas.cz; julien.bloino@sns.it

List of Figures

S1 MAE, |MAX|, standard deviation for modes 1-17 of methyloxirane S-12

S2 Fermi res.: Raman spectrum of meox in fingerprint region S-13

S3 Fermi res.: ROA spectrum of meox in fingerprint region S-14

S4 Fermi res.: IR spectrum of meox in CH-str. region (LXe) S-15

S5 Fermi res.: VCD spectrum of meox in CH-str. region (LXe) S-16

S6 Fermi res.: Raman spectrum of meox in CH-str. region S-17

S7 Fermi res.: ROA spectrum of meox in CH-str. region S-18

S8 Fermi res.: IR spectrum of meox in CH-str. region (CCl_4) S-19

S9 Fermi res.: VCD spectrum of meox in CH-str. region (CCl_4) S-20

S10 1-1 DDR: Raman spectrum of meox in CH-str. region ($K_I^{1-2} = 0.3/0.4/0.5$) . S-22

S11 1-1 DDR: ROA spectrum of meox in CH-str. region ($K_I^{1-2} = 0.3/0.4/0.5$) . . S-23

S12 1-1 DDR: IR spectrum of meox in CH-str. region (LXe, $K_I^{1-2} = 0.1/0.2/0.3$) S-24

S13 1-1 DDR: VCD spectrum of meox in CH-str. region (LXe, $K_I^{1-2} = 0.1/0.2/0.3$)S-25

S14 1-1 DDR: IR spectrum of meox in CH-str. region (CCl_4, $K_I^{1-2} = 0.1/0.2/0.3$) S-26

S15 1-1 DDR: VCD spectrum of meox in CH-str. region (CCl_4, $K_I^{1-2} = 0.1/0.2/0.3$)S-27

S16 1-1 DDR: Raman spectrum of meox in CH-str. region ($K_I^{1-2} = 0.1/0.2/0.3$) . S-28

S17 1-1 DDR: ROA spectrum of meox in CH-str. region ($K_I^{1-2} = 0.1/0.2/0.3$) . . S-29

S18 1-1 DDR: IR spectrum of meox in fingerprint region ($K_I^{1-2} = 0.1/0.2/0.3$) . . S-30

S19 1-1 DDR: VCD spectrum of meox in fingerprint region ($K_I^{1-2} = 0.1/0.2/0.3$) S-31

S20 1-1 DDR: Raman spectrum of meox in fingerpring region ($K_I^{1-2} = 0.1/0.2/0.3$)S-32

S21 1-1 DDR: ROA spectrum of meox in fingerpring region ($K_I^{1-2} = 0.1/0.2/0.3$) S-33

S22 Final IR/VCD spectra of methyloxirane (full y scale) S-34

S23 Final Raman/ROA spectra of methyloxirane (full y scale) S-35

S24 1-1 DDR: IR spectrum of α-pinene in fingerprint region ($K_I^{1-2} = 0.1/0.3/0.5$) S-37

S25 1-1 DDR: IR spectrum of α-pinene in CH-str. region ($K_I^{1-2} = 0.1/0.3/0.5$) . S-38

S26 1-1 DDR: VCD spectrum of α-pinene in fingerprint region ($K_I^{1-2} = 0.1/0.3/0.5$)S-39

S27 1-1 DDR: VCD spectrum of α-pinene in CH-str. region ($K_I^{1-2} = 0.1/0.3/0.5$) S-40

S28 LAM effect on IR spectrum of α-pinene in fingerprint region S-41

S29 LAM effect on IR spectrum of α-pinene in CH-stretching region S-42

S30 LAM effect on VCD spectrum of α-pinene in fingerprint region S-43

S31 LAM effect on VCD spectrum of α-pinene in CH-stretching region S-44

S32 IR and VCD spectra of artemisinin (shifted) S-46

List of Tables

S1 DVPT2 transition moments of $\langle \mathbf{P} \rangle_{0,1_{22}}$ S-21

Transition moments of properties

By expressing the perturbed wave functions into the RSPT development of the transition moment of a property $\mathbf{P}$, the following form is reached,

$$\langle \mathbf{P} \rangle_{I,F} = \langle \boldsymbol{v}_I | \mathbf{P}^{(0)} | \boldsymbol{v}_F \rangle \tag{1a}$$

$$+ \langle \boldsymbol{v}_I | \mathbf{P}^{(1)} | \boldsymbol{v}_F \rangle \tag{1b}$$

$$+ \sum_{r \neq I} \frac{\langle \boldsymbol{v}_I | \mathcal{H}^{(1)} | \boldsymbol{v}_r \rangle \langle \boldsymbol{v}_r | \mathbf{P}^{(0)} | \boldsymbol{v}_F \rangle}{\varepsilon_I{}^{(0)} - \varepsilon_r{}^{(0)}} \tag{1c}$$

$$+ \sum_{r \neq F} \frac{\langle \boldsymbol{v}_I | \mathbf{P}^{(0)} | \boldsymbol{v}_r \rangle \langle \boldsymbol{v}_r | \mathcal{H}^{(1)} | \boldsymbol{v}_F \rangle}{\varepsilon_F{}^{(0)} - \varepsilon_r{}^{(0)}} \tag{1d}$$

$$+ \langle \boldsymbol{v}_I | \mathbf{P}^{(2)} | \boldsymbol{v}_F \rangle \tag{1e}$$

$$+ \sum_{r,s \neq I} \frac{\langle \boldsymbol{v}_I | \mathcal{H}^{(1)} | \boldsymbol{v}_r \rangle \langle \boldsymbol{v}_r | \mathcal{H}^{(1)} | \boldsymbol{v}_s \rangle \langle \boldsymbol{v}_s | \mathbf{P}^{(0)} | \boldsymbol{v}_F \rangle}{\left(\varepsilon_I{}^{(0)} - \varepsilon_s{}^{(0)}\right)\left(\varepsilon_I{}^{(0)} - \varepsilon_r{}^{(0)}\right)} + \sum_{r \neq I} \frac{\langle \boldsymbol{v}_I | \mathcal{H}^{(2)} | \boldsymbol{v}_r \rangle \langle \boldsymbol{v}_r | \mathbf{P}^{(0)} | \boldsymbol{v}_F \rangle}{\varepsilon_I{}^{(0)} - \varepsilon_r{}^{(0)}} \tag{1f}$$

$$+ \sum_{r,s \neq F} \frac{\langle \boldsymbol{v}_I | \mathbf{P}^{(0)} | \boldsymbol{v}_r \rangle \langle \boldsymbol{v}_r | \mathcal{H}^{(1)} | \boldsymbol{v}_s \rangle \langle \boldsymbol{v}_s | \mathcal{H}^{(1)} | \boldsymbol{v}_F \rangle}{\left(\varepsilon_F{}^{(0)} - \varepsilon_s{}^{(0)}\right)\left(\varepsilon_F{}^{(0)} - \varepsilon_r{}^{(0)}\right)} + \sum_{r \neq F} \frac{\langle \boldsymbol{v}_I | \mathbf{P}^{(0)} | \boldsymbol{v}_r \rangle \langle \boldsymbol{v}_r | \mathcal{H}^{(2)} | \boldsymbol{v}_F \rangle}{\varepsilon_F{}^{(0)} - \varepsilon_r{}^{(0)}} \tag{1g}$$

$$+ \sum_{r \neq I} \frac{\langle \boldsymbol{v}_I | \mathcal{H}^{(1)} | \boldsymbol{v}_r \rangle \langle \boldsymbol{v}_r | \mathbf{P}^{(1)} | \boldsymbol{v}_F \rangle}{\varepsilon_I{}^{(0)} - \varepsilon_r{}^{(0)}} \tag{1h}$$

$$+ \sum_{r \neq F} \frac{\langle \boldsymbol{v}_I | \mathbf{P}^{(1)} | \boldsymbol{v}_r \rangle \langle \boldsymbol{v}_r | \mathcal{H}^{(1)} | \boldsymbol{v}_F \rangle}{\varepsilon_F{}^{(0)} - \varepsilon_r{}^{(0)}} \tag{1i}$$

$$+ \sum_{r \neq I, s \neq F} \frac{\langle \boldsymbol{v}_I | \mathcal{H}^{(1)} | \boldsymbol{v}_r \rangle \langle \boldsymbol{v}_r | \mathbf{P}^{(0)} | \boldsymbol{v}_s \rangle \langle \boldsymbol{v}_s | \mathcal{H}^{(1)} | \boldsymbol{v}_F \rangle}{\left(\varepsilon_I{}^{(0)} - \varepsilon_r{}^{(0)}\right)\left(\varepsilon_F{}^{(0)} - \varepsilon_s{}^{(0)}\right)} \tag{1j}$$

$$- \frac{\langle \boldsymbol{v}_I | \mathbf{P}^{(0)} | \boldsymbol{v}_F \rangle}{2} \left[\sum_{r \neq I} \frac{\langle \boldsymbol{v}_I | \mathcal{H}^{(1)} | \boldsymbol{v}_r \rangle \langle \boldsymbol{v}_r | \mathbf{P}^{(0)} | \boldsymbol{v}_I \rangle}{\left(\varepsilon_I{}^{(0)} - \varepsilon_r{}^{(0)}\right)^2} + \sum_{r \neq F} \frac{\langle \boldsymbol{v}_F | \mathcal{H}^{(1)} | \boldsymbol{v}_r \rangle \langle \boldsymbol{v}_r | \mathbf{P}^{(0)} | \boldsymbol{v}_F \rangle}{\left(\varepsilon_F{}^{(0)} - \varepsilon_r{}^{(0)}\right)^2} \right] \tag{1k}$$

Each line corresponds to a different term in Eq. 8 in the main article.

Resonances in Darling–Dennison terms

As mentioned in the main article, a particularly challenging aspect of the calculation of Darling–Dennison (DD) couplings is that they can be themselves affected by Fermi resonances. If such singularities occur, the corresponding terms would be incorrectly evaluated, with the risk of excessively large values predicted. While this problem is known and has been documented, few strategies have been proposed to identify these resonances, especially in ways compatible with black-box implementations. Indeed, with most studies focused on small molecules, a commonly chosen method is to flag manually resonances, based on the harmonic frequency differences at the denominator, or the overall magnitude of $\kappa_{\text{HRS}}^{A-B}$.[S1–S4] Conversely, ecause of the different nature with respect to the energy, tests like the one proposed by Martin and coworkers cannot be directly applied. While a test like **R12CVPT** could be used, it cannot be applied on the analytic formulas for the DD terms, but instead must be considered in the development of the equations, so that the resonances are correctly removed. This either requires the development and implementation of different formulas based on the possible resonance cases, or an explicit expansion of the perturbative terms at runtime.[S5] Both solutions can represent a significant increase in complexity for the implementation, with the latter having an important computational cost over the use of analytic formulas.

In this work, we have adapted a technique initially designed for the calculation of so-called resonance-free energies, based on the on the degeneracy-corrected PT2 (DCPT2) treatment proposed by Kuhler and coworkers[S6] and called the hybrid DCPT2-VPT2 scheme.[S7] The principle is similar to Martin's test and designed for the calculation of vibrational energies. Considering the first-order correction to the energy of two harmonic states, potentially involved in a Fermi resonance and assumed uncoupled from the rest, the variational energy can be written

$$E = \frac{{\varepsilon_r}^{(0)} + {\varepsilon_s}^{(0)}}{2} \pm \sqrt{\Delta^2 + k^2} \tag{2}$$

where k is proportional to the cubic force constant related to the modes involved in states r and s and $\Delta = ({\varepsilon_r}^{(0)} - {\varepsilon_s}^{(0)})/2$. For simplicity, Δ and k are assumed positive. This can be easily generalized by factorizing the whole term by $+1$ or -1, using absolute values of these quantities. The square root can then be expanded with respect to k up to the second order,

$$\sqrt{\Delta^2 + k^2} \approx \Delta + \frac{k^2}{2\Delta} \tag{3}$$

so that the potentially resonant term can be recast as,

$$\frac{k^2}{2\Delta} \approx \sqrt{\Delta^2 + k^2} - \Delta \tag{4}$$

By choosing appropriately k, the previous equality can be generalized to other quantities, not specific to energy, such as those appearing in the analytic formulas of Darling–Dennison terms.[S8] The main limitation of this transformation is that it is valid for negligible couplings ($k \sim 0$), and would introduce a significant error compared to the correct term for large values of k far from resonances. To compensate this, an hybridization is operated, by combining the original term (f_{orig}) with the alternative form obtained through the DCPT2 scheme (f_{corr}) with a transition function such as an hyperbolic tangent,[S7,S8]

$$f = \Lambda f_{\text{orig}} + (1 - \Lambda) f_{\text{corr}} \quad ; \quad \Lambda = \frac{\tanh\bigl(a[\sqrt{k^2\Delta^2} - b]\bigr) + 1}{2} \tag{5}$$

b controls the transition threshold from the corrected to the original term, and can be set at relatively high value (e.g., $b \sim 10^5$), while a controls the degree of mixing permitted during the transition. Step-like functions can be adopted with $a = 1$. This form can be easily implemented with analytic formulas of Darling–Dennison resonances and have been used here.

Extension of VPT2/GVPT2 to 3 quanta

Analytic transition moments at the VPT2 level

The transition moments and coefficients associated to 3-quanta states (second overtones, "2+1" binary combinations and "1+1+1" ternary combinations), can be generalized in few equations, by introducing the constant factor,

$$A = \frac{1}{\sqrt{(1 + \delta_{ij} + \delta_{ik})!}}$$

The transitions have then the form,[S9–S11]

$$
\begin{aligned}
&\langle \mathbf{P}\rangle_{0,(1+\delta_{ij}+\delta_{ik})_i(1-\delta_{ij})_j(1-\delta_{ik})_k} = \\
&\quad s_2(\mathbf{P}_{ijk}+\mathbf{P}_{ikj}+\mathbf{P}_{jki})\mathrm{S} \\
&\quad + A\sum_{l=1}^{N}\Bigg\{\frac{s_0}{4}\mathsf{f}_{ijkl}\mathbf{P}_l\left[\frac{\mathrm{S}}{\omega_i+\omega_j+\omega_k-\omega_l}-\frac{1}{\omega_i+\omega_j+\omega_k+\omega_l}\right] \\
&\qquad + \frac{s_0}{2}\sum_{\tau} B^{\mathrm{eq}}_{\tau}\mathbf{P}_l\Bigg\{\Bigg[\zeta_{ij,\tau}\zeta_{kl,\tau}\left(\sqrt{\frac{\omega_j\omega_l}{\omega_i\omega_k}}-\sqrt{\frac{\omega_i\omega_l}{\omega_j\omega_k}}\right)+\zeta_{ik,\tau}\zeta_{jl,\tau}\left(\sqrt{\frac{\omega_k\omega_l}{\omega_i\omega_j}}-\sqrt{\frac{\omega_i\omega_l}{\omega_j\omega_k}}\right) \\
&\qquad\qquad + \zeta_{il,\tau}\zeta_{jk,\tau}\left(\sqrt{\frac{\omega_k\omega_l}{\omega_i\omega_j}}-\sqrt{\frac{\omega_j\omega_l}{\omega_i\omega_k}}\right)\Bigg]\times\left[\frac{1}{\omega_i+\omega_j+\omega_k+\omega_l}+\frac{\mathrm{S}}{\omega_i+\omega_j+\omega_k-\omega_l}\right] \\
&\qquad\quad + \Bigg[\zeta_{ij,\tau}\zeta_{kl,\tau}\left(\sqrt{\frac{\omega_i\omega_k}{\omega_j\omega_l}}-\sqrt{\frac{\omega_j\omega_k}{\omega_i\omega_l}}\right)+\zeta_{ik,\tau}\zeta_{jl,\tau}\left(\sqrt{\frac{\omega_i\omega_j}{\omega_k\omega_l}}-\sqrt{\frac{\omega_j\omega_k}{\omega_i\omega_l}}\right) \\
&\qquad\qquad + \zeta_{il,\tau}\zeta_{jk,\tau}\left(\sqrt{\frac{\omega_i\omega_j}{\omega_k\omega_l}}-\sqrt{\frac{\omega_i\omega_k}{\omega_j\omega_l}}\right)\Bigg]\times\left[\frac{1}{\omega_i+\omega_j+\omega_k+\omega_l}-\frac{\mathrm{S}}{\omega_i+\omega_j+\omega_k-\omega_l}\right]\Bigg\} \\
&\qquad + \frac{s_1}{4}\Bigg\{\mathsf{f}_{ijl}\left[\frac{\mathrm{S}(\mathbf{P}_{kl}+\mathbf{P}_{lk})}{\omega_i+\omega_j-\omega_l}-\frac{\mathbf{P}_{kl}+\mathrm{S}\mathbf{P}_{lk}}{\omega_i+\omega_j+\omega_l}\right]+\mathsf{f}_{ikl}\left[\frac{\mathrm{S}(\mathbf{P}_{jl}+\mathbf{P}_{lj})}{\omega_i+\omega_k-\omega_l}-\frac{\mathbf{P}_{jl}+\mathrm{S}\mathbf{P}_{lj}}{\omega_i+\omega_k+\omega_l}\right] \\
&\qquad\qquad + \mathsf{f}_{jkl}\left[\frac{\mathrm{S}(\mathbf{P}_{il}+\mathbf{P}_{li})}{\omega_j+\omega_k-\omega_l}-\frac{\mathbf{P}_{il}+\mathrm{S}\mathbf{P}_{li}}{\omega_j+\omega_k+\omega_l}\right]\Bigg\} \\
&\qquad + \frac{s_0}{8}\sum_{m=1}^{N}\mathsf{f}_{ijm}\mathsf{f}_{klm}\mathbf{P}_l\Bigg[\frac{1}{(\omega_m+\omega_j+\omega_i)(\omega_l+\omega_k+\omega_j+\omega_i)}+\frac{1}{(\omega_m-\omega_j-\omega_i)(\omega_l+\omega_k+\omega_j+\omega_i)} \\
&\qquad\qquad + \frac{\mathrm{S}}{(\omega_m+\omega_j+\omega_i)(\omega_l-\omega_k-\omega_j-\omega_i)}+\frac{\mathrm{S}}{(\omega_m-\omega_j-\omega_i)(\omega_l-\omega_k-\omega_j-\omega_i)}\Bigg] \\
&\qquad\quad + \mathsf{f}_{ikm}\mathsf{f}_{jlm}\mathbf{P}_l\Bigg[\frac{1}{(\omega_m+\omega_k+\omega_i)(\omega_l+\omega_k+\omega_j+\omega_i)}+\frac{1}{(\omega_m-\omega_k-\omega_i)(\omega_l+\omega_k+\omega_j+\omega_i)} \\
&\qquad\qquad + \frac{\mathrm{S}}{(\omega_m+\omega_k+\omega_i)(\omega_l-\omega_k-\omega_j-\omega_i)}+\frac{\mathrm{S}}{(\omega_m-\omega_k-\omega_i)(\omega_l-\omega_k-\omega_j-\omega_i)}\Bigg] \\
&\qquad\quad + \mathsf{f}_{jkm}\mathsf{f}_{ilm}\mathbf{P}_l\Bigg[\frac{1}{(\omega_m+\omega_k+\omega_j)(\omega_l+\omega_k+\omega_j+\omega_i)}+\frac{1}{(\omega_m-\omega_k-\omega_j)(\omega_l+\omega_k+\omega_j+\omega_i)} \\
&\qquad\qquad + \frac{\mathrm{S}}{(\omega_m+\omega_k+\omega_j)(\omega_l-\omega_k-\omega_j-\omega_i)}+\frac{\mathrm{S}}{(\omega_m-\omega_k-\omega_j)(\omega_l-\omega_k-\omega_j-\omega_i)}\Bigg]\Bigg\}
\end{aligned}
\tag{6}
$$

Coefficients for the second-order peturbed wave function

The coefficient of the second-order wave function involved in the 1-3 Darling–Dennison resonance has the form,

$$
\begin{aligned}
&C_{1_i1_j1_k,1_l}{}^{(2)} = \\
&\quad A\sum_{m=1}^{N}\Bigg\{\frac{1}{16}\mathsf{f}_{imm}\mathsf{f}_{jkl}\left[\frac{1}{(\omega_i+\omega_j+\omega_k-\omega_l)(\omega_j+\omega_k-\omega_l)}+\frac{1}{(\omega_i+\omega_j+\omega_k-\omega_l)\omega_i}\right] \\
&\qquad +\frac{1}{8}\mathsf{f}_{ilm}\mathsf{f}_{jkm}\left[\frac{1}{(\omega_i+\omega_j+\omega_k-\omega_l)(\omega_j+\omega_k-\omega_m)}+\frac{1}{(\omega_i+\omega_j+\omega_k-\omega_l)(\omega_i-\omega_l-\omega_m)}\right] \\
&\qquad +\frac{1}{8}\mathsf{f}_{ikm}\mathsf{f}_{jlm}\left[\frac{1}{(\omega_i+\omega_j+\omega_k-\omega_l)(\omega_j-\omega_l-\omega_m)}+\frac{1}{(\omega_i+\omega_j+\omega_k-\omega_l)(\omega_i+\omega_k-\omega_m)}\right] \\
&\qquad +\frac{1}{16}\mathsf{f}_{ikl}\mathsf{f}_{jmm}\left[\frac{1}{(\omega_i+\omega_j+\omega_k-\omega_l)\omega_j}+\frac{1}{(\omega_i+\omega_j+\omega_k-\omega_l)(\omega_i+\omega_k-\omega_l)}\right] \\
&\qquad +\frac{1}{8}\mathsf{f}_{ijm}\mathsf{f}_{klm}\left[\frac{1}{(\omega_i+\omega_j+\omega_k-\omega_l)(\omega_k-\omega_l-\omega_m)}+\frac{1}{(\omega_i+\omega_j+\omega_k-\omega_l)(\omega_i+\omega_j-\omega_m)}\right] \\
&\qquad +\frac{1}{16}\mathsf{f}_{ijl}\mathsf{f}_{kmm}\left[\frac{1}{(\omega_i+\omega_j+\omega_k-\omega_l)\omega_k}+\frac{1}{(\omega_i+\omega_j+\omega_k-\omega_l)(\omega_i+\omega_j-\omega_l)}\right] \\
&\qquad +\frac{1}{16}\mathsf{f}_{ijk}\mathsf{f}_{lmm}\left[-\frac{1}{(\omega_i+\omega_j+\omega_k-\omega_l)\omega_l}+\frac{1}{(\omega_i+\omega_j+\omega_k-\omega_l)(\omega_i+\omega_j+\omega_k)}\right]\Bigg\} \\
&\quad +\frac{A}{4}\frac{\mathsf{f}_{ijkl}}{\omega_i+\omega_j+\omega_k-\omega_l} \\
&\quad +\frac{A}{2}\sum_{\tau}B_\tau^{\mathrm{eq}}\Bigg\{\zeta_{ij}\zeta_{kl}\left(\frac{1}{\omega_i+\omega_j+\omega_k-\omega_l}\right)\left[\sqrt{\frac{\omega_j\omega_l}{\omega_i\omega_k}}+\sqrt{\frac{\omega_j\omega_k}{\omega_i\omega_l}}-\sqrt{\frac{\omega_i\omega_l}{\omega_j\omega_k}}-\sqrt{\frac{\omega_i\omega_k}{\omega_j\omega_l}}\right] \\
&\qquad \zeta_{ik}\zeta_{jl}\left(\frac{1}{\omega_i+\omega_j+\omega_k-\omega_l}\right)\left[\sqrt{\frac{\omega_k\omega_l}{\omega_i\omega_j}}+\sqrt{\frac{\omega_j\omega_k}{\omega_i\omega_l}}-\sqrt{\frac{\omega_i\omega_l}{\omega_j\omega_k}}-\sqrt{\frac{\omega_i\omega_j}{\omega_k\omega_l}}\right] \\
&\qquad \zeta_{jk}\zeta_{il}\left(\frac{1}{\omega_i+\omega_j+\omega_k-\omega_l}\right)\left[\sqrt{\frac{\omega_k\omega_l}{\omega_i\omega_j}}+\sqrt{\frac{\omega_i\omega_k}{\omega_j\omega_l}}-\sqrt{\frac{\omega_j\omega_l}{\omega_i\omega_k}}-\sqrt{\frac{\omega_i\omega_j}{\omega_k\omega_l}}\right]\Bigg\}
\end{aligned}
\tag{7}
$$

Non-resonant form of the VPT2 transition moments

Fundamental bands

In the case where $|1_i\rangle$ and $|1_j\rangle$ are involved in a 1-1 DDR, an alternative formula must be adopted for the transition moment given in Eq. 11.

$$
\begin{aligned}
\langle \mathbf{P}\rangle_{0,1_i} =& \frac{s_0}{16}\sum_{j,k,l=1}^{N} \mathsf{f}_{ikl}\mathsf{f}_{jkl}\boldsymbol{P}_j \Bigg\{ (1-\delta_{ij})(1-\delta_{ik})(1-\delta_{il}) \Bigg[\frac{1}{(\omega_i+\omega_j)(\omega_i+\omega_k+\omega_l)} - \frac{1}{(\omega_i+\omega_j)(\omega_i-\omega_k-\omega_l)} \\
&\quad + \frac{\mathrm{S}}{(\omega_i+\omega_k+\omega_l)(\omega_j+\omega_k+\omega_l)} \Bigg] \\
&\quad + \delta_{ij}(1+\delta_{ik})(1-\delta_{il}) \Bigg[\frac{1}{2\omega_i(\omega_i+\omega_k+\omega_l)} - \frac{1}{2\omega_i(\omega_i-\omega_k-\omega_l)} + \frac{\mathrm{S}}{2(\omega_i+\omega_k+\omega_l)^2} \\
&\quad - \frac{\mathrm{S}}{2(\omega_i-\omega_k-\omega_l)^2} \Bigg] \\
&\quad + (1-\delta_{ij})(1-\delta_{ik})\delta_{il} \Bigg[\frac{4}{\omega_k(\omega_i+\omega_j)} + \frac{2}{(\omega_i+\omega_j)(2\omega_i+\omega_k)} \\
&\quad - \frac{\mathrm{S}}{\omega_k(\omega_i-\omega_j-\omega_k)} + \frac{2\mathrm{S}}{(\omega_i+\omega_j+\omega_k)(2\omega_i+\omega_k)} \Bigg] \Bigg\} \\
& + \mathsf{f}_{ijk}\mathsf{f}_{llk}\boldsymbol{P}_j \Bigg\{ \frac{\delta_{ij}}{\omega_i\omega_k}\left(1+\frac{\delta_{ik}\delta_{il}(6-4\mathrm{S})}{9}\right) \\
&\quad + (1-\delta_{ij})(1-\delta_{ik})(1-\delta_{il}) \Bigg[\frac{2}{\omega_k(\omega_i+\omega_j)} - \frac{\mathrm{S}}{\omega_k(\omega_i-\omega_j-\omega_k)} \Bigg] \\
&\quad + \delta_{ik}(1-\delta_{ij}) \Bigg[\frac{2(1+2\delta_{il}/3)}{\omega_i(\omega_i+\omega_j)} + \frac{3\mathrm{S}}{\omega_i\omega_j} + \frac{\delta_{il}\mathrm{S}}{3\omega_i(2\omega_i+\omega_j)} \Bigg] \Bigg\}
\end{aligned}
\tag{8}
$$

3-quanta transitions

In the case where $|1_i1_j1_k\rangle$ and $|1_l\rangle$ are involved in a 1-3 DDR, an alternative formula must be adopted for the transition moment given in Eq. S3,

$$
\begin{aligned}
&\langle \boldsymbol{P}\rangle_{0,(1+\delta_{ij}+\delta_{ik})_i(1-\delta_{ij})_j(1-\delta_{ik})_k} = \\
&\quad s_2(\boldsymbol{P}_{ijk}+\boldsymbol{P}_{ikj}+\boldsymbol{P}_{jki})\mathrm{S} \\
&\quad + A\sum_{l=1}^{N}\Bigg\{\frac{s_0}{4}\mathsf{f}_{ijkl}\boldsymbol{P}_l\left[-\frac{1}{\omega_i+\omega_j+\omega_k+\omega_l}\right] \\
&\qquad + \frac{s_0}{2}\sum_{\tau}B^{\mathrm{eq}}_{\tau}\boldsymbol{P}_l\Bigg\{\Bigg[\zeta_{ij,\tau}\zeta_{kl,\tau}\left(\sqrt{\frac{\omega_j\omega_l}{\omega_i\omega_k}}+\sqrt{\frac{\omega_i\omega_k}{\omega_j\omega_l}}-\sqrt{\frac{\omega_i\omega_l}{\omega_j\omega_k}}-\sqrt{\frac{\omega_j\omega_k}{\omega_i\omega_l}}\right) \\
&\qquad\quad + \zeta_{ik,\tau}\zeta_{jl,\tau}\left(\sqrt{\frac{\omega_k\omega_l}{\omega_i\omega_j}}+\sqrt{\frac{\omega_i\omega_j}{\omega_k\omega_l}}-\sqrt{\frac{\omega_i\omega_l}{\omega_j\omega_k}}-\sqrt{\frac{\omega_j\omega_k}{\omega_i\omega_l}}\right) \\
&\qquad\quad + \zeta_{il,\tau}\zeta_{jk,\tau}\left(\sqrt{\frac{\omega_k\omega_l}{\omega_i\omega_j}}+\sqrt{\frac{\omega_i\omega_j}{\omega_k\omega_l}}-\sqrt{\frac{\omega_j\omega_l}{\omega_i\omega_k}}-\sqrt{\frac{\omega_i\omega_k}{\omega_j\omega_l}}\right)\Bigg]\times\frac{1}{\omega_i+\omega_j+\omega_k+\omega_l}\Bigg\} \\
&\qquad + \frac{s_1}{4}\Bigg\{\mathsf{f}_{ijl}\left[\frac{\mathrm{S}(\boldsymbol{P}_{kl}+\boldsymbol{P}_{lk})}{\omega_i+\omega_j-\omega_l}-\frac{\boldsymbol{P}_{kl}+\mathrm{S}\boldsymbol{P}_{lk}}{\omega_i+\omega_j+\omega_l}\right]+\mathsf{f}_{ikl}\left[\frac{\mathrm{S}(\boldsymbol{P}_{jl}+\boldsymbol{P}_{lj})}{\omega_i+\omega_k-\omega_l}-\frac{\boldsymbol{P}_{jl}+\mathrm{S}\boldsymbol{P}_{lj}}{\omega_i+\omega_k+\omega_l}\right] \\
&\qquad\quad + \mathsf{f}_{jkl}\left[\frac{\mathrm{S}(\boldsymbol{P}_{il}+\boldsymbol{P}_{li})}{\omega_j+\omega_k-\omega_l}-\frac{\boldsymbol{P}_{il}+\mathrm{S}\boldsymbol{P}_{li}}{\omega_j+\omega_k+\omega_l}\right]\Bigg\} \\
&\qquad + \frac{s_0}{8}\sum_{m=1}^{N}\mathsf{f}_{ijm}\mathsf{f}_{klm}\boldsymbol{P}_l\Bigg[\frac{1}{(\omega_m+\omega_j+\omega_i)(\omega_l+\omega_k+\omega_j+\omega_i)}+\frac{1}{(\omega_m-\omega_j-\omega_i)(\omega_l+\omega_k+\omega_j+\omega_i)} \\
&\qquad\quad - \frac{\mathrm{S}}{(\omega_m+\omega_j+\omega_i)(\omega_k-\omega_l-\omega_m)}\Bigg] \\
&\qquad\quad + \mathsf{f}_{ikm}\mathsf{f}_{jlm}\boldsymbol{P}_l\Bigg[\frac{1}{(\omega_m+\omega_k+\omega_i)(\omega_l+\omega_k+\omega_j+\omega_i)}+\frac{1}{(\omega_m-\omega_k-\omega_i)(\omega_l+\omega_k+\omega_j+\omega_i)} \\
&\qquad\quad - \frac{\mathrm{S}}{(\omega_m+\omega_k+\omega_i)(\omega_j-\omega_l-\omega_m)}\Bigg] \\
&\qquad\quad + \mathsf{f}_{jkm}\mathsf{f}_{ilm}\boldsymbol{P}_l\Bigg[\frac{1}{(\omega_m+\omega_k+\omega_j)(\omega_l+\omega_k+\omega_j+\omega_i)}+\frac{1}{(\omega_m-\omega_k-\omega_j)(\omega_l+\omega_k+\omega_j+\omega_i)} \\
&\qquad\quad - \frac{\mathrm{S}}{(\omega_m+\omega_k+\omega_j)(\omega_i-\omega_l-\omega_m)}\Bigg] \\
&\qquad\quad + \mathsf{f}_{ijl}\mathsf{f}_{kmm}\boldsymbol{P}_l\left[\frac{\mathrm{S}}{2\omega_k(\omega_l-\omega_i-\omega_j)}\right]+\mathsf{f}_{ikl}\mathsf{f}_{jmm}\boldsymbol{P}_l\left[\frac{\mathrm{S}}{2\omega_j(\omega_l-\omega_i-\omega_k)}\right] \\
&\qquad\quad + \mathsf{f}_{jkl}\mathsf{f}_{imm}\boldsymbol{P}_l\left[\frac{\mathrm{S}}{2\omega_i(\omega_l-\omega_j-\omega_k)}\right]+\mathsf{f}_{ijk}\mathsf{f}_{lmm}\boldsymbol{P}_l\left[\frac{\mathrm{S}}{2\omega_l(\omega_i+\omega_j+\omega_k)}\right]\Bigg\}
\end{aligned}
\tag{9}
$$

Additional figures and tables on methyloxirane

Error on energy

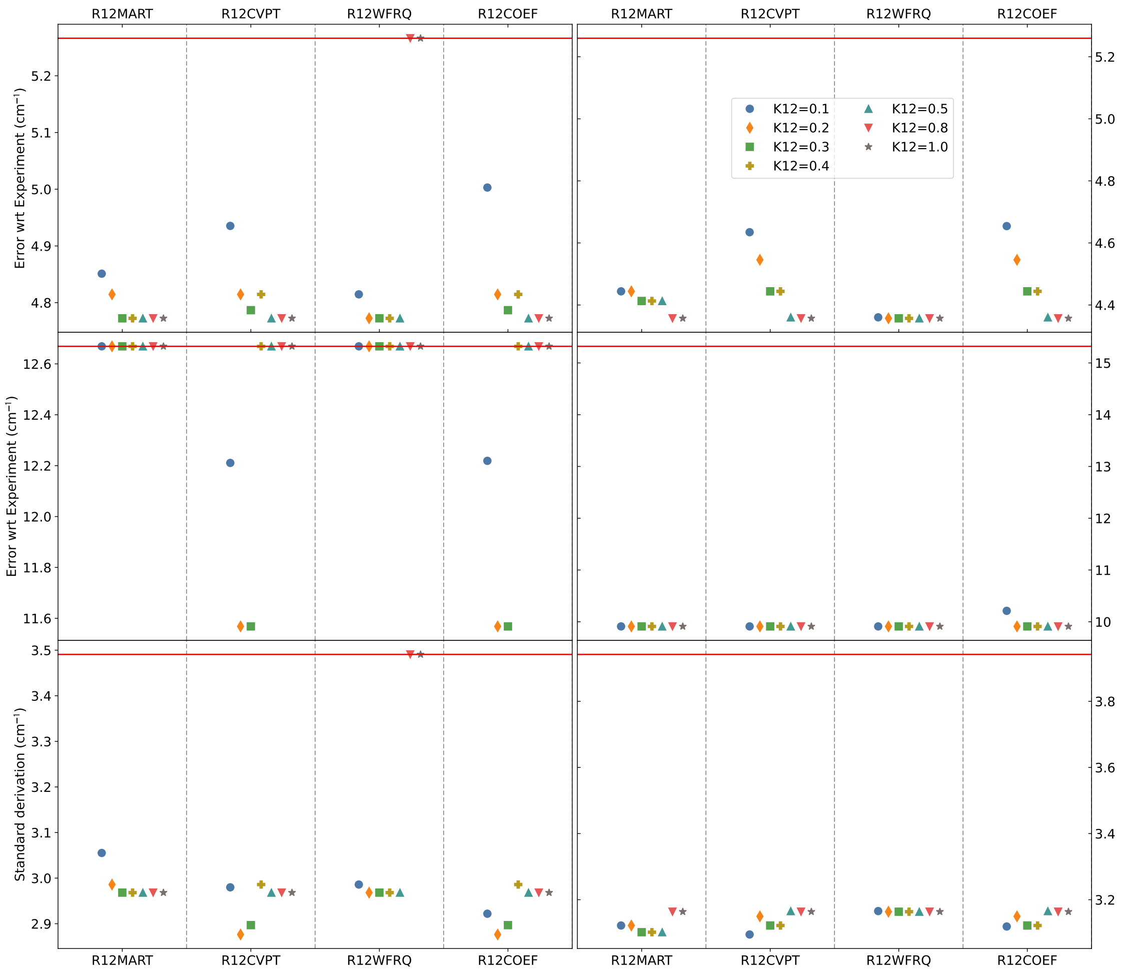

Figure S1: Mean absolute error (MAE, upper panel), maximum unsigned error (|MAX|, center panel), and standard deviation (lower panel) for the fundamental energies below 2800 cm^{-1} for methyloxirane in gas phase (left panels) and neat liquid (right panels), excluding mode 18 (17 modes out of 24). Experimental data were taken from Refs. S12,S13. To facilitate comparisons, the GVPT2 energies were used, selecting the variational overlap with the highest overlap over the fundamental DVPT2 states. **R12COEF** and **R12WFRQ** were applied as unique tests, without any energy-relative test. The red horizontal line represents the error if no Fermi resonances are treated.

Thresholds on Fermi resonances

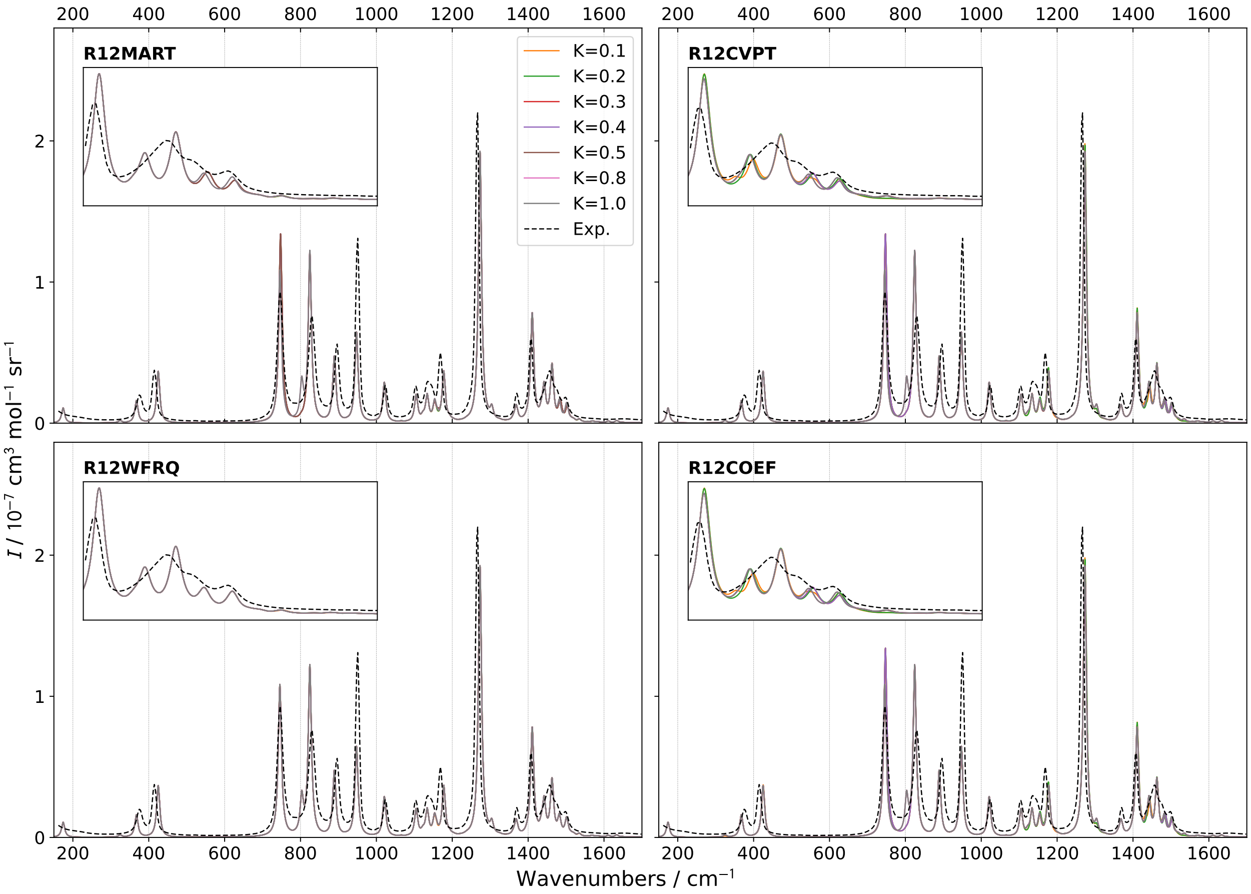

Figure S2: Comparison of theoretical GVPT2 Raman spectra of (S)-2-methyloxirane in neat liquid within the fingerprint region using different schemes and thresholds (K=K^{1-2}) for the identification of Fermi resonances. Experiment (black dashed lines) was taken from Ref. S13. Gaussian broadening functions with half-width at half-maximum of 5 cm^{-1} were used to match experiment. The inset shows a zoom of the 1400–1600 cm^{-1} region.

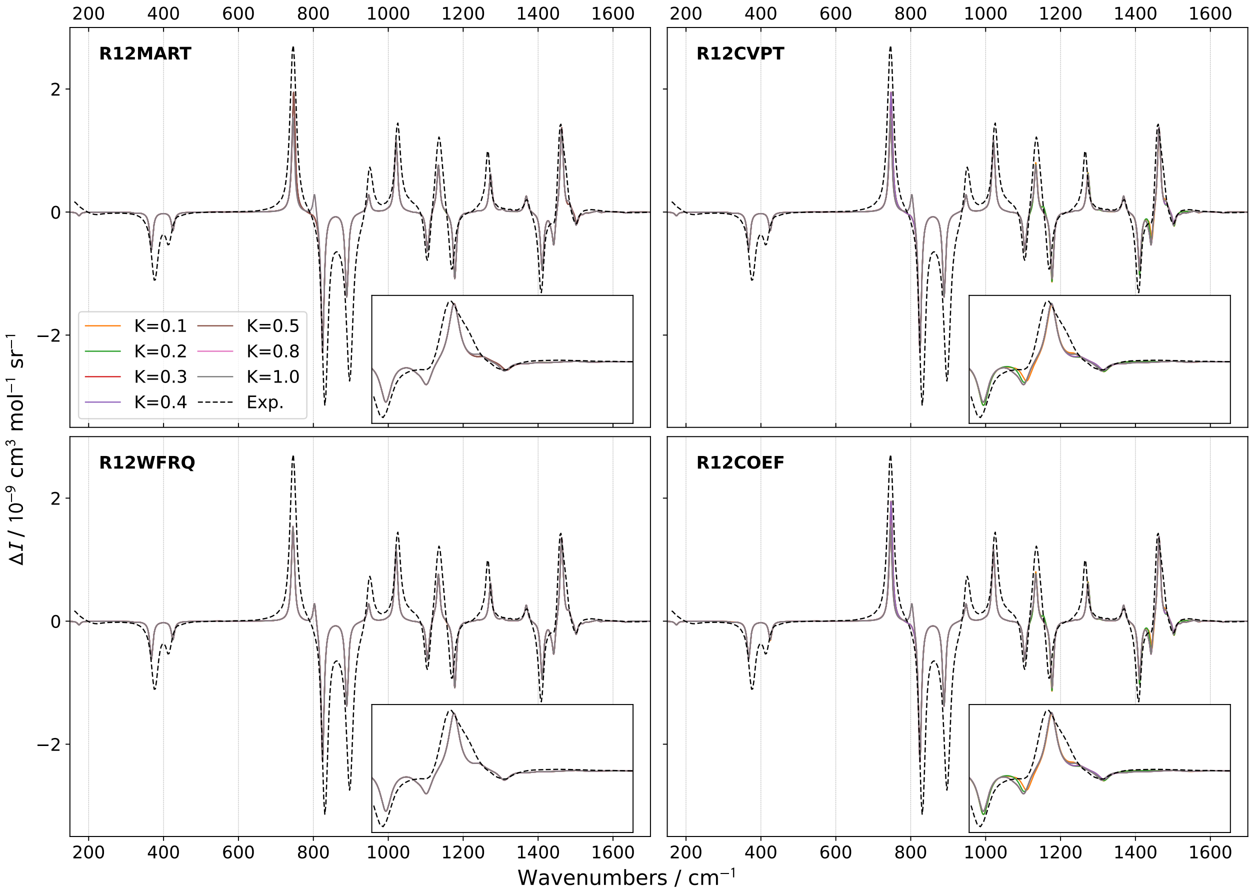

Figure S3: Comparison of theoretical GVPT2 ROA spectra of (S)-2-methyloxirane in neat liquid within the fingerprint region using different schemes and thresholds (K=K^{1-2}) for the identification of Fermi resonances. Experiment (black dashed lines) was taken from Ref. S13. Gaussian broadening functions with half-width at half-maximum of 5 cm^{-1} were used to match experiment. The inset shows a zoom of the 1400–1600 cm^{-1} region.

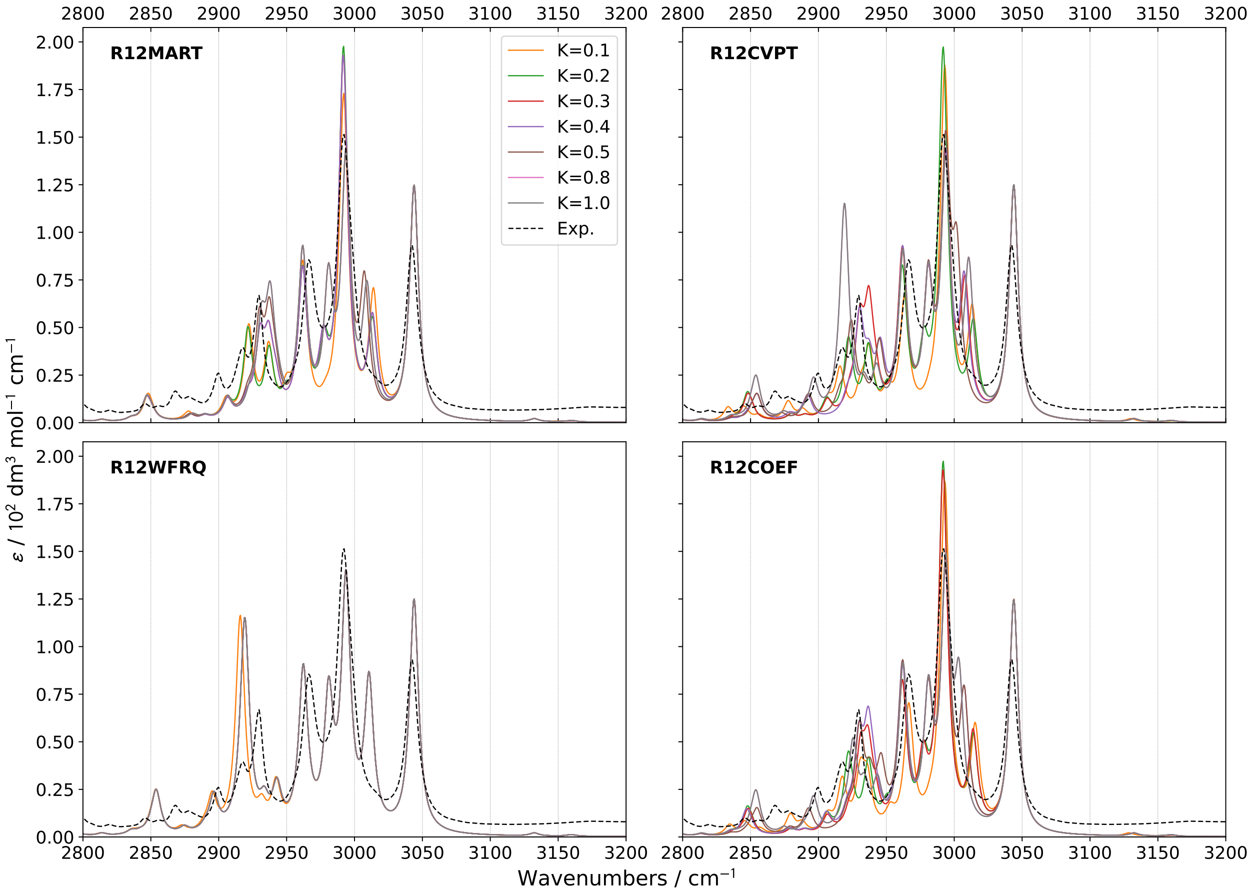

Figure S4: Comparison of theoretical GVPT2 IR spectra of (S)-2-methyloxirane in liquid xenon within the CH-stretching region using different schemes and thresholds (K=K^{1-2}) for the identification of Fermi resonances. Experiment (black dashed lines) was taken from Ref. S14. Lorentzian broadening functions with half-width at half-maximum of 4 cm^{-1} were used to match experiment. The computed spectra were shifted by -22 cm^{-1} to match experiment.

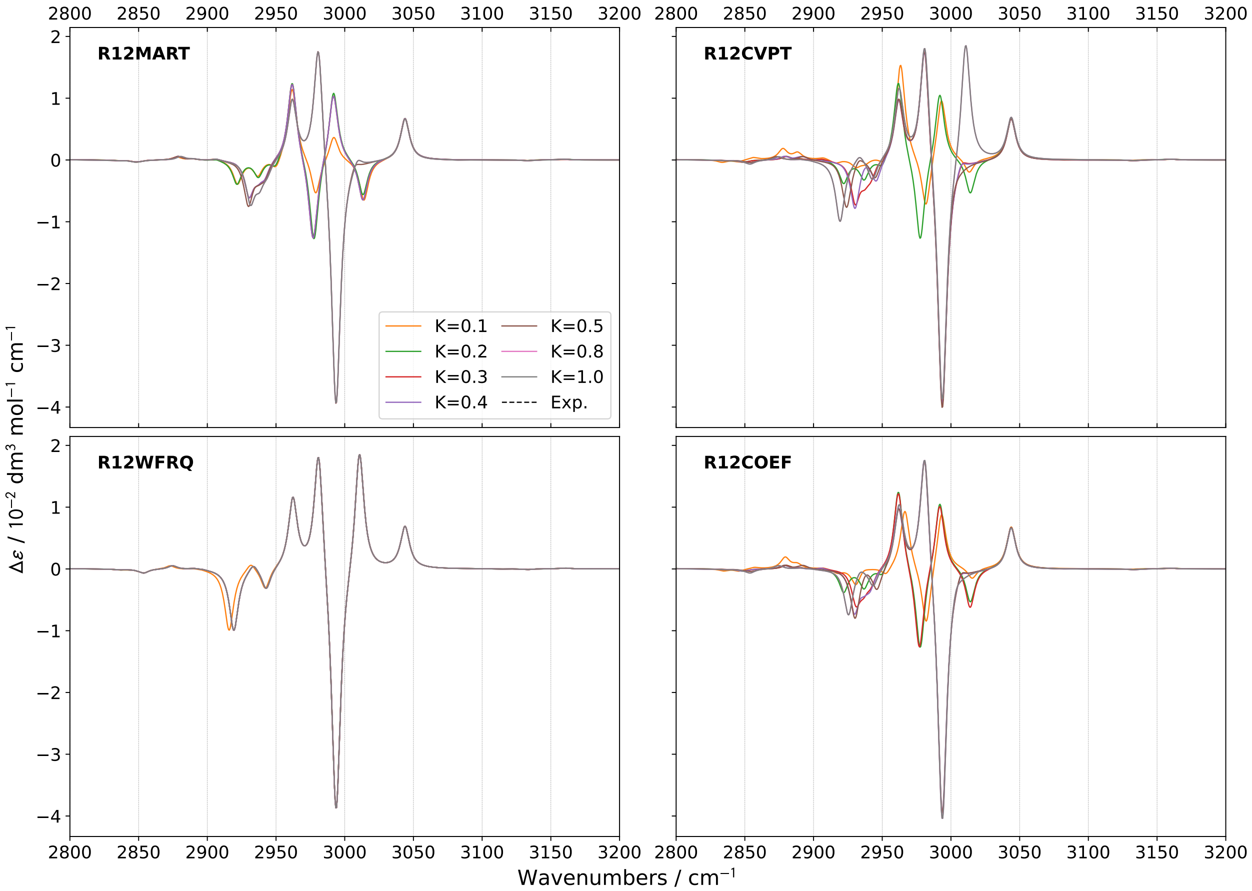

Figure S5: Comparison of theoretical GVPT2 VCD spectra of (S)-2-methyloxirane in liquid xenon within the CH-stretching region using different schemes and thresholds (K=K^{1-2}) for the identification of Fermi resonances. Experiment (black dashed lines) was taken from Ref. S14. Lorentzian broadening functions with half-width at half-maximum of 4 cm^{-1} were used to match experiment. The computed spectra were shifted by -22 cm^{-1} to match experiment.

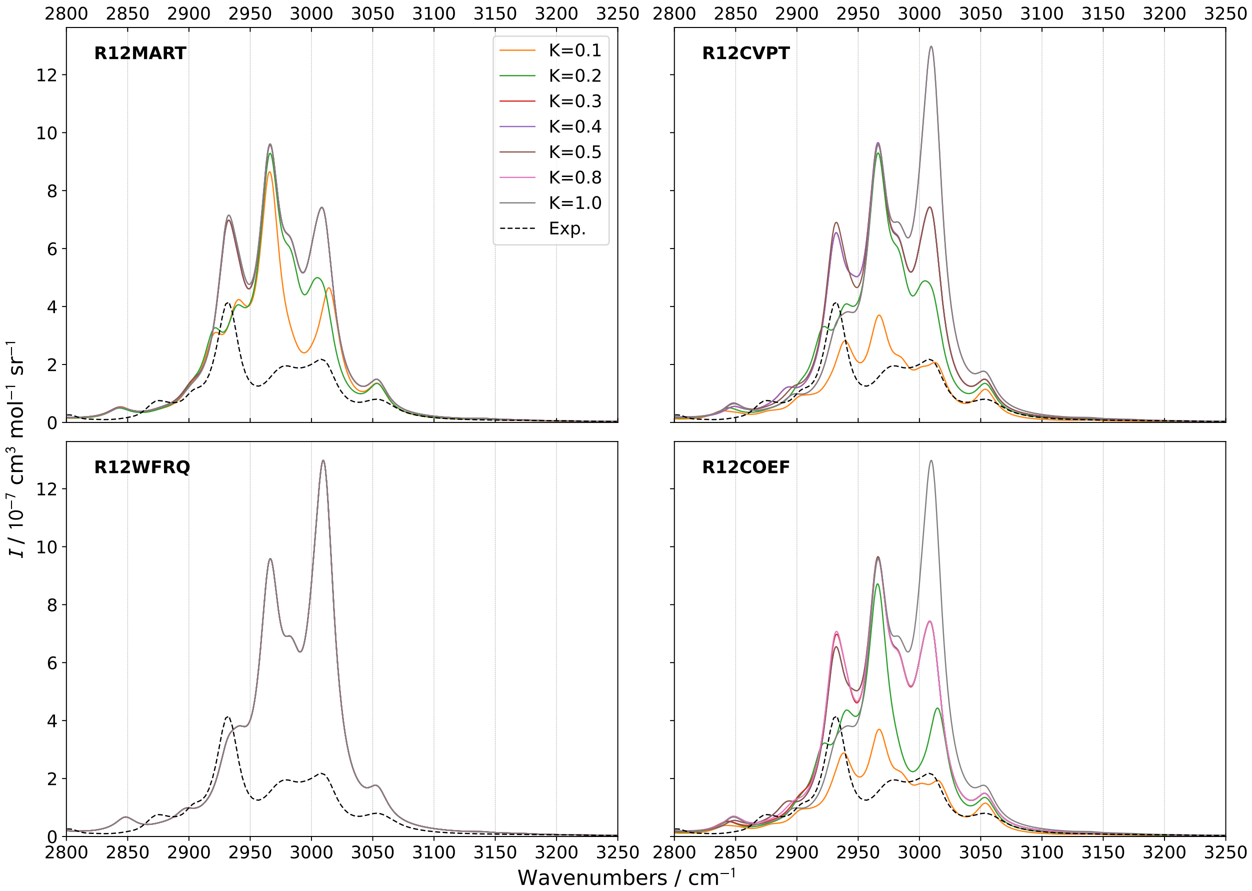

Figure S6: Comparison of theoretical GVPT2 Raman spectra of (S)-2-methyloxirane in neat liquid within the CH-stretching region using different schemes and thresholds (K=K^{1-2}) for the identification of Fermi resonances. Experiment (black dashed lines) was taken from Ref. S13. Gaussian broadening functions with half-width at half-maximum of 10 cm^{-1} were used to match experiment. The computed spectra were shifted by -18 cm^{-1} to match experiment.

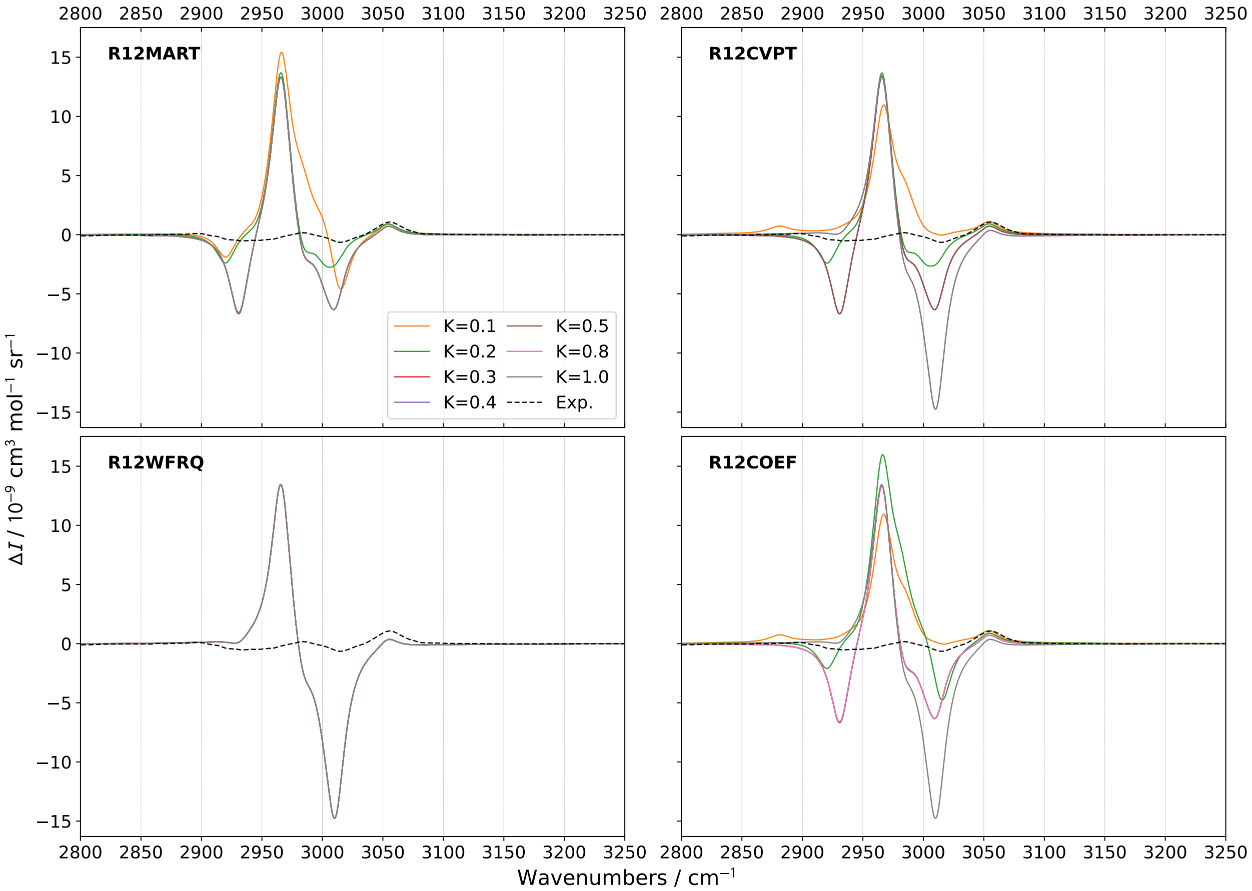

Figure S7: Comparison of theoretical GVPT2 ROA spectra of (S)-2-methyloxirane in neat liquid within the CH-stretching region using different schemes and thresholds (K=K^{1-2}) for the identification of Fermi resonances. Experiment (black dashed lines) was taken from Ref. S13. Gaussian broadening functions with half-width at half-maximum of 10 cm^{-1} were used to match experiment. The computed spectra were shifted by -18 cm^{-1} to match experiment.

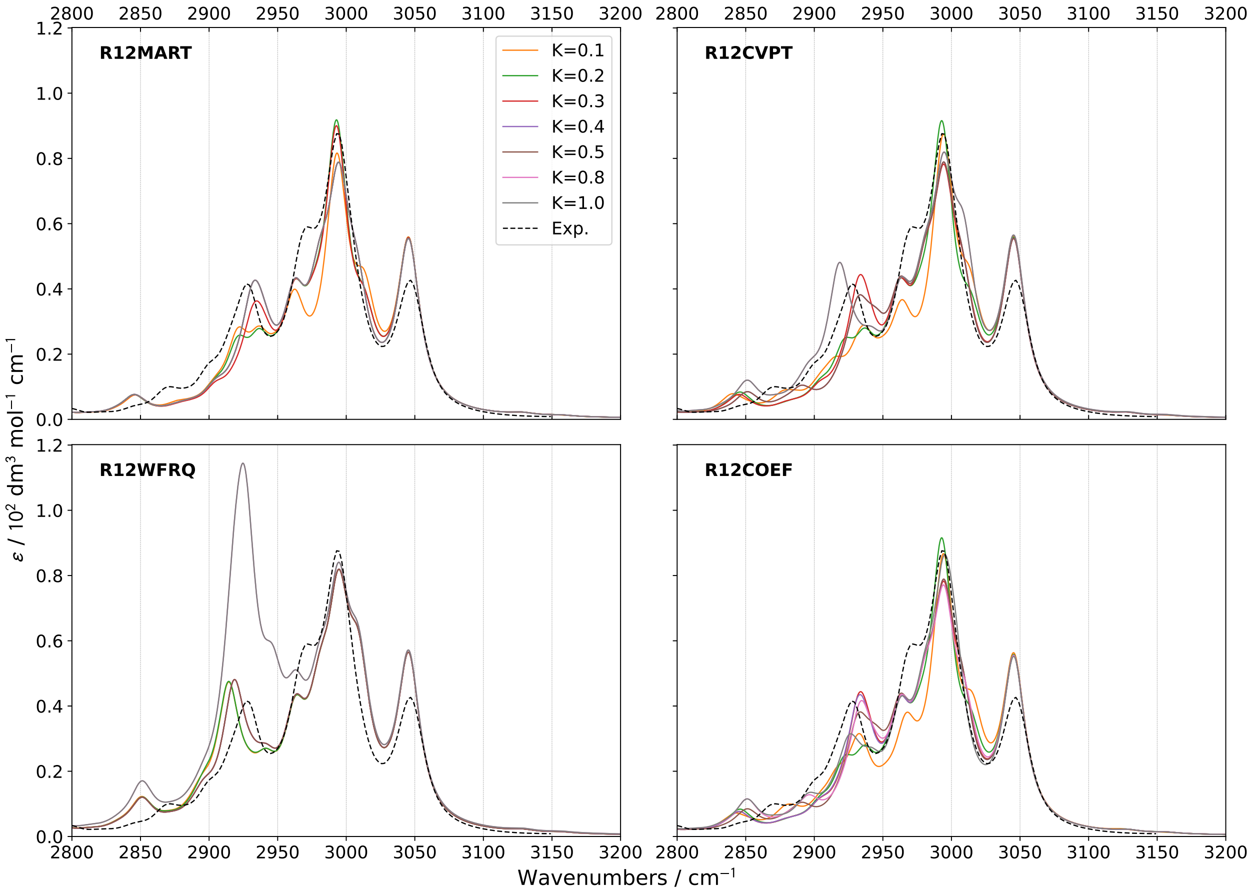

Figure S8: Comparison of theoretical GVPT2 IR spectra of (S)-2-methyloxirane in CCl_4 within the CH-stretching region using different schemes and thresholds (K=K^{1-2}) for the identification of Fermi resonances. Experiment (black dashed lines) was taken from Ref. S15. Lorentzian broadening functions with half-width at half-maximum of 10 cm^{-1} were used to match experiment. The computed spectra were shifted by -22 cm^{-1} to match experiment.

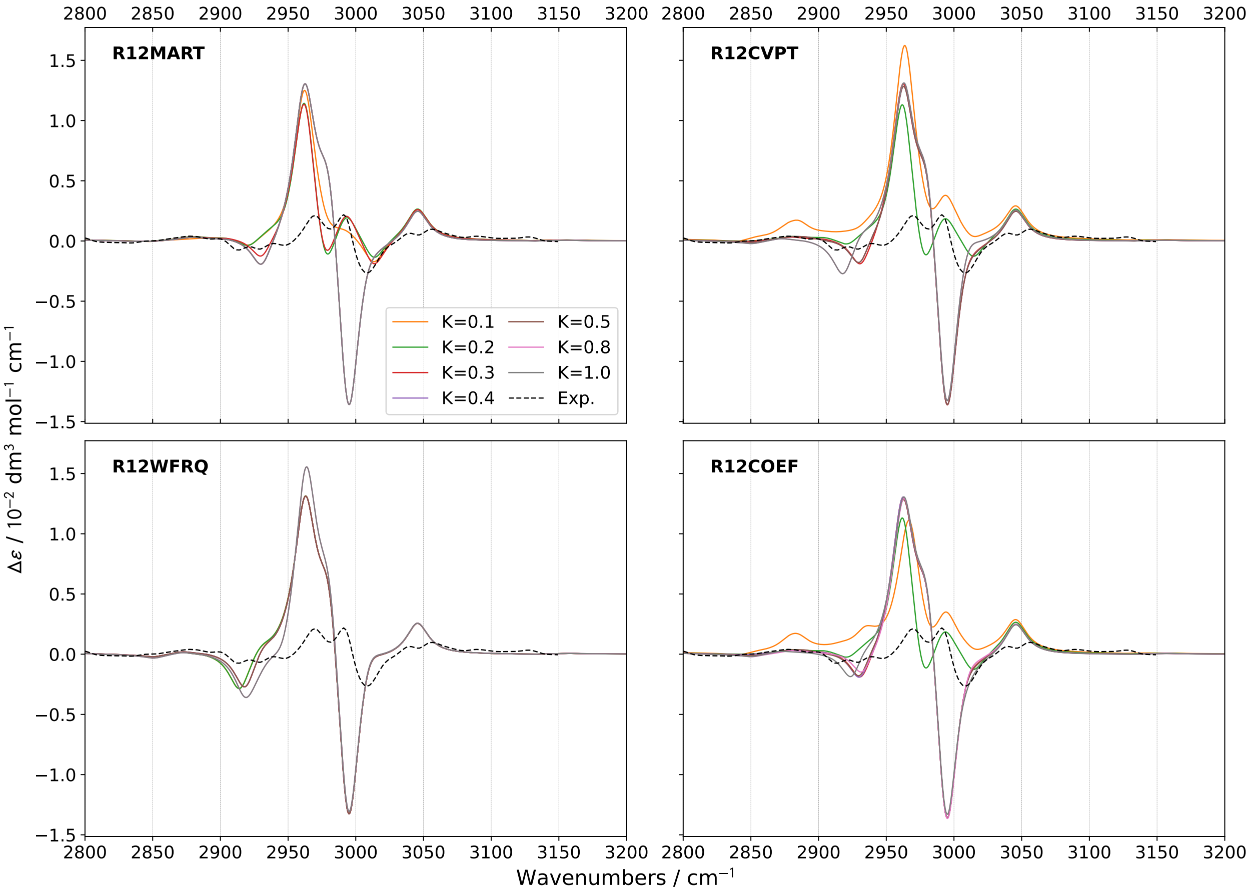

Figure S9: Comparison of theoretical GVPT2 VCD spectra of (S)-2-methyloxirane in CCl_4 within the CH-stretching region using different schemes and thresholds (K=K^{1-2}) for the identification of Fermi resonances. Experiment (black dashed lines) was taken from Ref. S15. Lorentzian broadening functions with half-width at half-maximum of 10 cm^{-1} were used to match experiment. The computed spectra were shifted by -22 cm^{-1} to match experiment.

Thresholds on 1-1 Darling–Dennison resonances

Table S1: DVPT2 transition moments $\langle \mathbf{P} \rangle_{0,1_{22}}$ of the electric and magnetic dipoles in atomic units. Resonances were identified with a combination of R12MART/R12COEF ($K^{1-2} = 1.0$) for Fermi resonances and R11HRS/R11COEF ($K^{1-1} = 10$) for 1-1 Darling–Dennison resonances. The variable criteria are specified in the left column.

Criteria	x	y	z
Electric dipole			
$K_I^{1-2} = 0.1$, $K_I^{1-1} = 0.1$	-0.347044e-03	0.223993e-02	-0.666927e-04
$K_I^{1-2} = 0.2$, $K_I^{1-1} = 0.1$	-0.304193e-02	0.275593e-02	-0.537648e-02
$K_I^{1-2} = 0.2$, $K_I^{1-1} = 1.0$	-0.562161e-02	0.108158e-01	-0.355303e-02
Magnetic dipole			
$K_I^{1-2} = 0.1$, $K_I^{1-1} = 0.1$	0.193775e+00	0.601831e+00	0.176862e+00
$K_I^{1-2} = 0.2$, $K_I^{1-1} = 0.1$	0.189465e+00	0.446221e+00	0.175600e+00
$K_I^{1-2} = 0.2$, $K_I^{1-1} = 1.0$	0.181141e+00	0.618876e+00	0.295739e+00

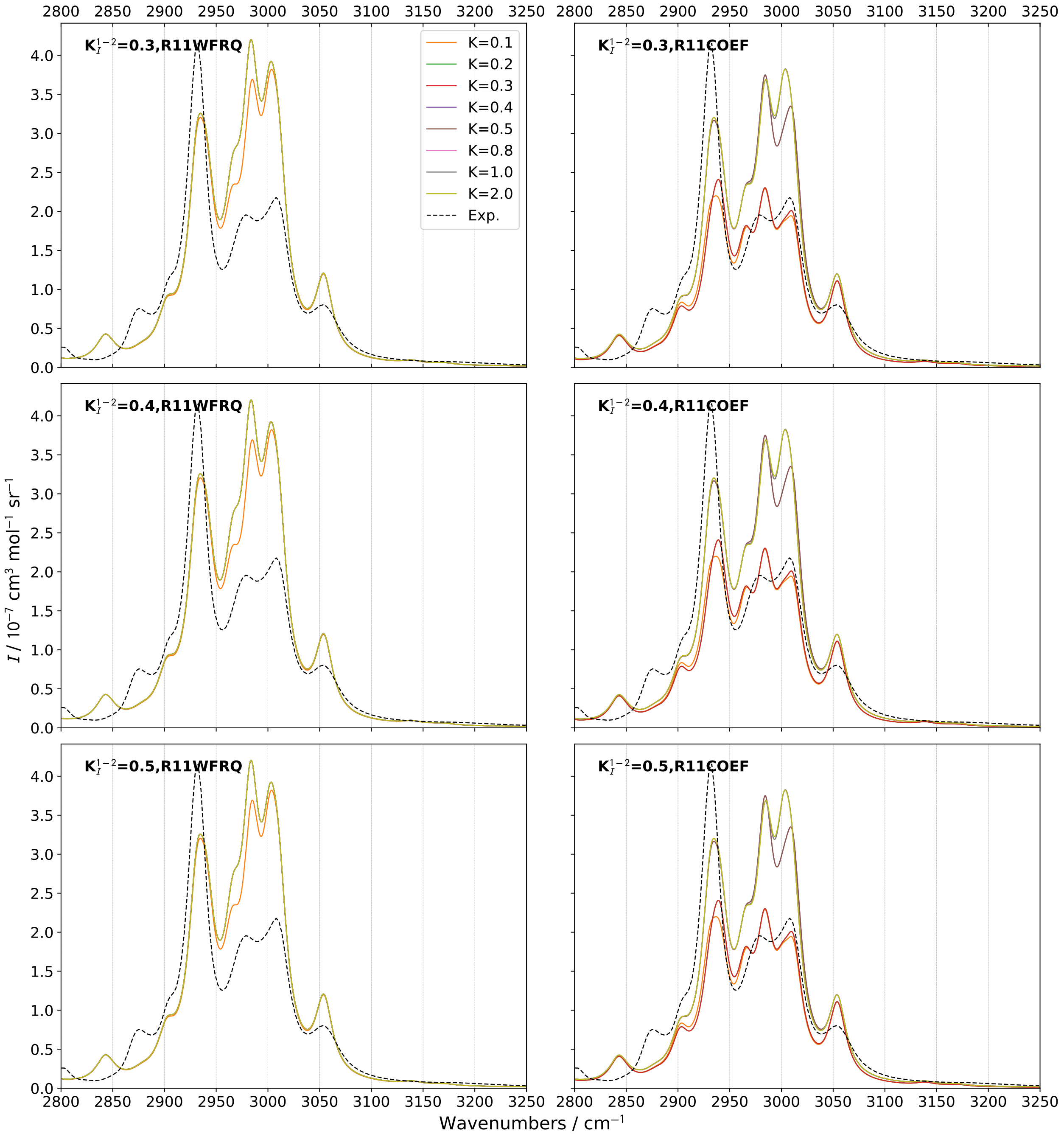

Figure S10: Comparison of theoretical GVPT2 Raman spectra of (S)-2-methyloxirane in neat liquid within the CH-stretching region using different schemes and thresholds (K=K_I^{1-1}) for the identification of 1-1 Darling–Dennison resonances. A combination of **R12MART** (K^{1-2} = 1.0) and **R12COEF** was used for the Fermi resonances, the second test with the thresholds K_I^{1-2}=0.3 (upper panels), 0.4 (middle panels) and 0.5 (lower panels). Experiment (black dashed lines) was taken from Ref. S13. Gaussian broadening functions with half-width at half-maximum of 10 cm^{-1} were used to match experiment. The computed spectra were shifted by -18 cm^{-1} to match experiment.

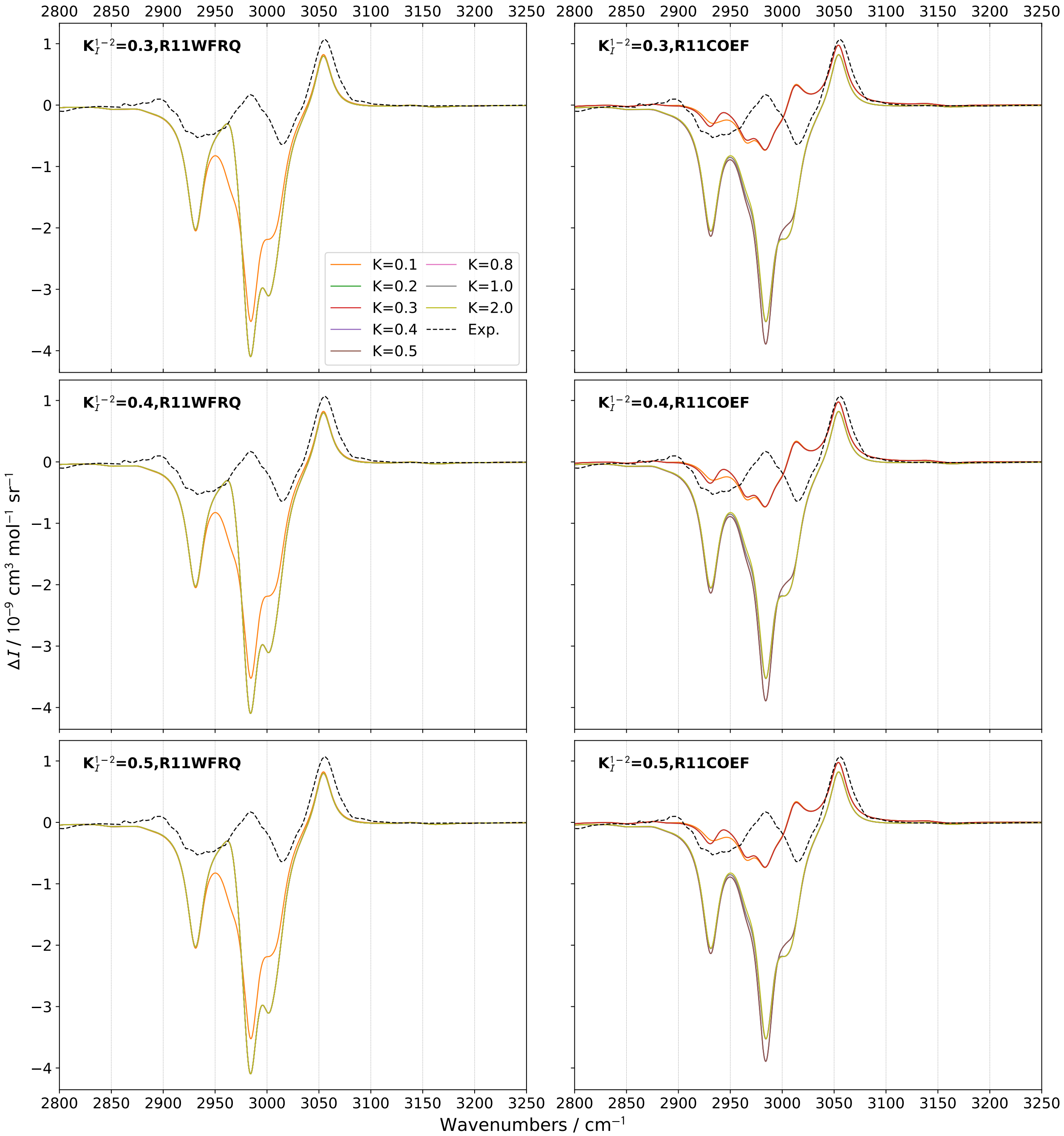

Figure S11: Comparison of theoretical GVPT2 ROA spectra of (S)-2-methyloxirane in neat liquid within the CH-stretching region using different schemes and thresholds (K=K_I^{1-1}) for the identification of 1-1 Darling–Dennison resonances. A combination of **R12MART** ($K^{1-2} = 1.0$) and **R12COEF** was used for the Fermi resonances, the second test with the thresholds K_I^{1-2}=0.3 (upper panels), 0.4 (middle panels) and 0.5 (lower panels). Experiment (black dashed lines) was taken from Ref. S13. Gaussian broadening functions with half-width at half-maximum of 10 cm^{-1} were used to match experiment. The computed spectra were shifted by -18 cm^{-1} to match experiment.

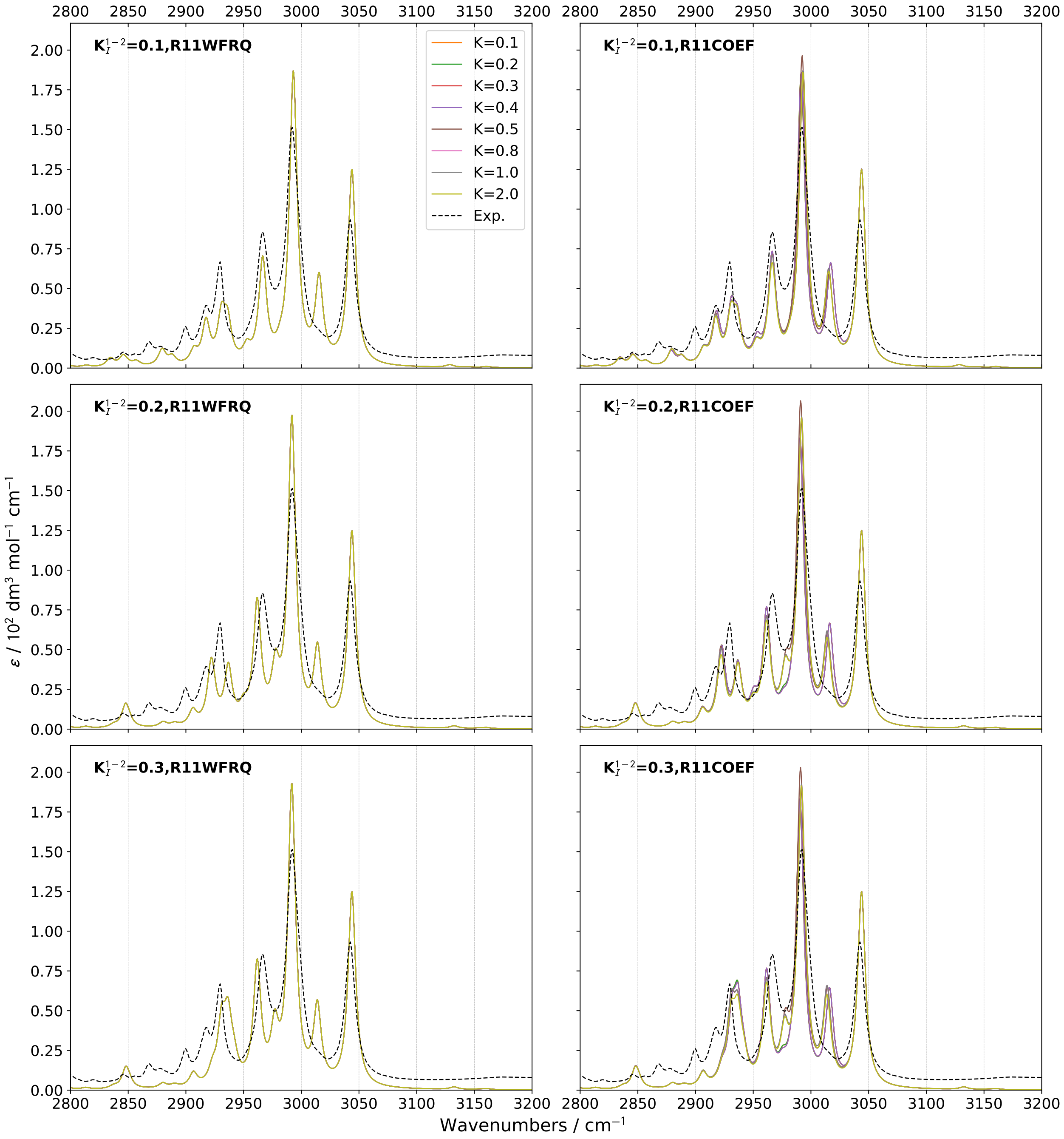

Figure S12: Comparison of theoretical GVPT2 IR spectra of (S)-2-methyloxirane in liquid xenon within the CH-stretching region using different schemes and thresholds (K=K_I^{1-1}) for the identification of 1-1 Darling–Dennison resonances. A combination of **R12MART** ($K^{1-2} = 1.0$) and **R12COEF** was used for the Fermi resonances, the second test with the thresholds K_I^{1-2}=0.1 (upper panels), 0.2 (middle panels) and 0.3 (lower panels). Experiment (black dashed lines) was taken from Ref. S14. Lorentzian broadening functions with half-width at half-maximum of 4 cm^{-1} were used to match experiment. The computed spectra were shifted by -22 cm^{-1} to match experiment.

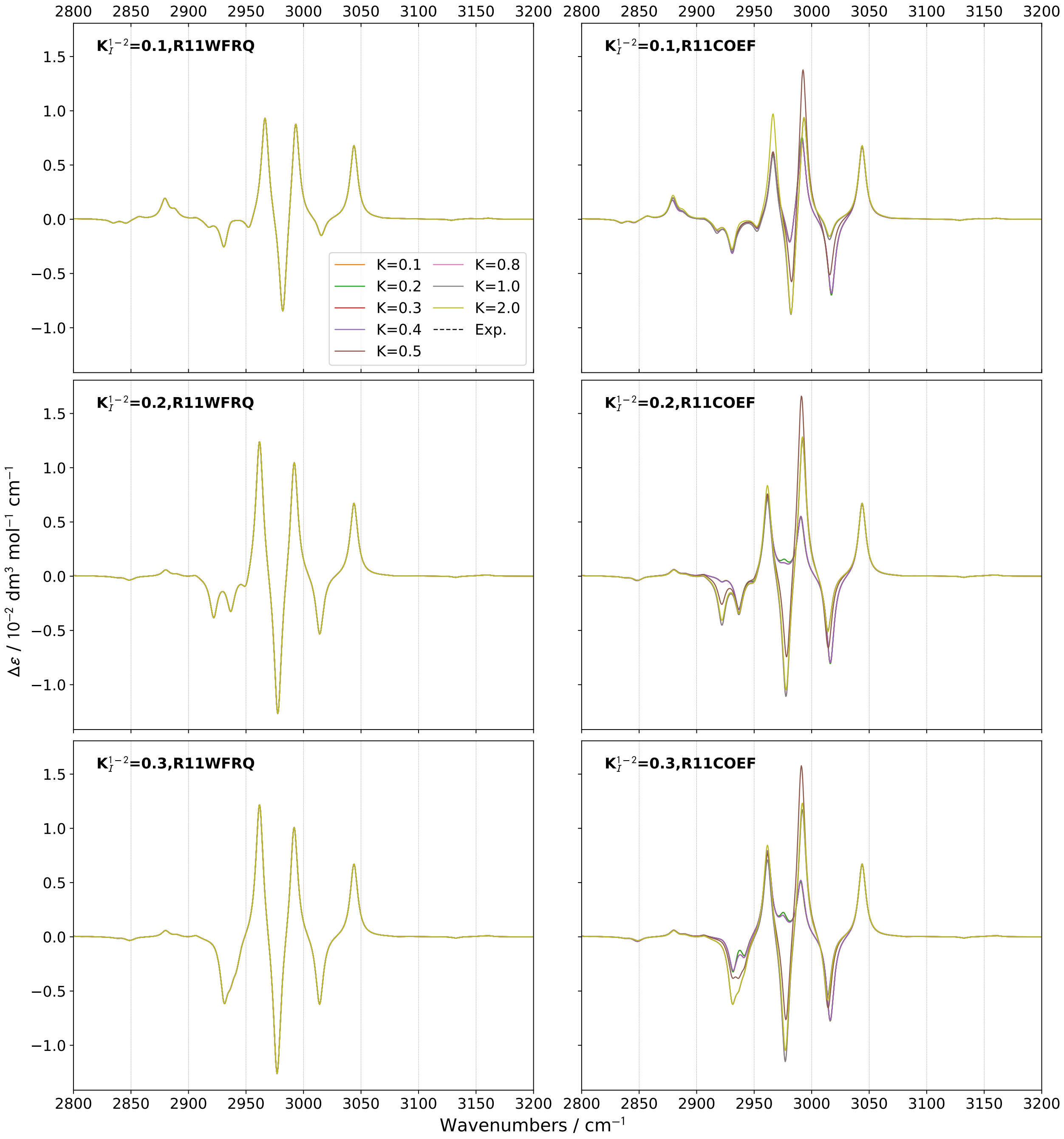

Figure S13: Comparison of theoretical GVPT2 VCD spectra of (S)-2-methyloxirane in liquid xenon within the CH-stretching region using different schemes and thresholds (K=K_I^{1-1}) for the identification of 1-1 Darling–Dennison resonances. A combination of **R12MART** ($K^{1-2} = 1.0$) and **R12COEF** was used for the Fermi resonances, the second test with the thresholds K_I^{1-2}=0.1 (upper panels), 0.2 (middle panels) and 0.3 (lower panels). Experiment (black dashed lines) was taken from Ref. S14. Lorentzian broadening functions with half-width at half-maximum of 4 cm^{-1} were used. The computed spectra were shifted by -22 cm^{-1} for consistency with the IR spectrum.

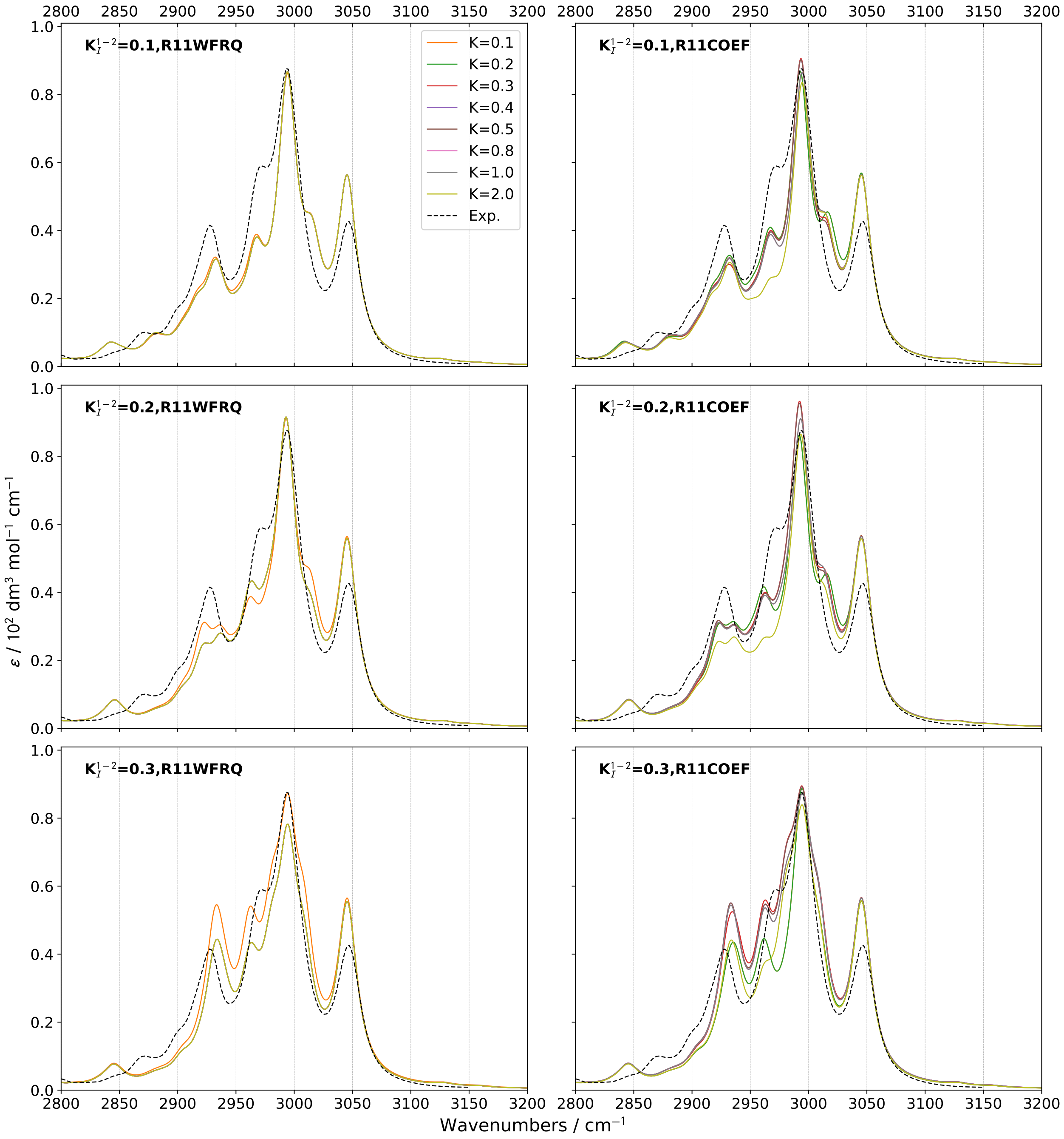

Figure S14: Comparison of theoretical GVPT2 IR spectra of (S)-2-methyloxirane in CCl_4 within the CH-stretching region using different schemes and thresholds (K=K_I^{1-1}) for the identification of 1-1 Darling–Dennison resonances. A combination of **R12MART** (K^{1-2} = 1.0) and **R12COEF** was used for the Fermi resonances, the second test with the thresholds K_I^{1-2}=0.1 (upper panels), 0.2 (middle panels) and 0.3 (lower panels). Experiment (black dashed lines) was taken from Ref. S15. Lorentzian broadening functions with half-width at half-maximum of 10 cm^{-1} were used to match experiment. The computed spectra were shifted by -22 cm^{-1} to match experiment.

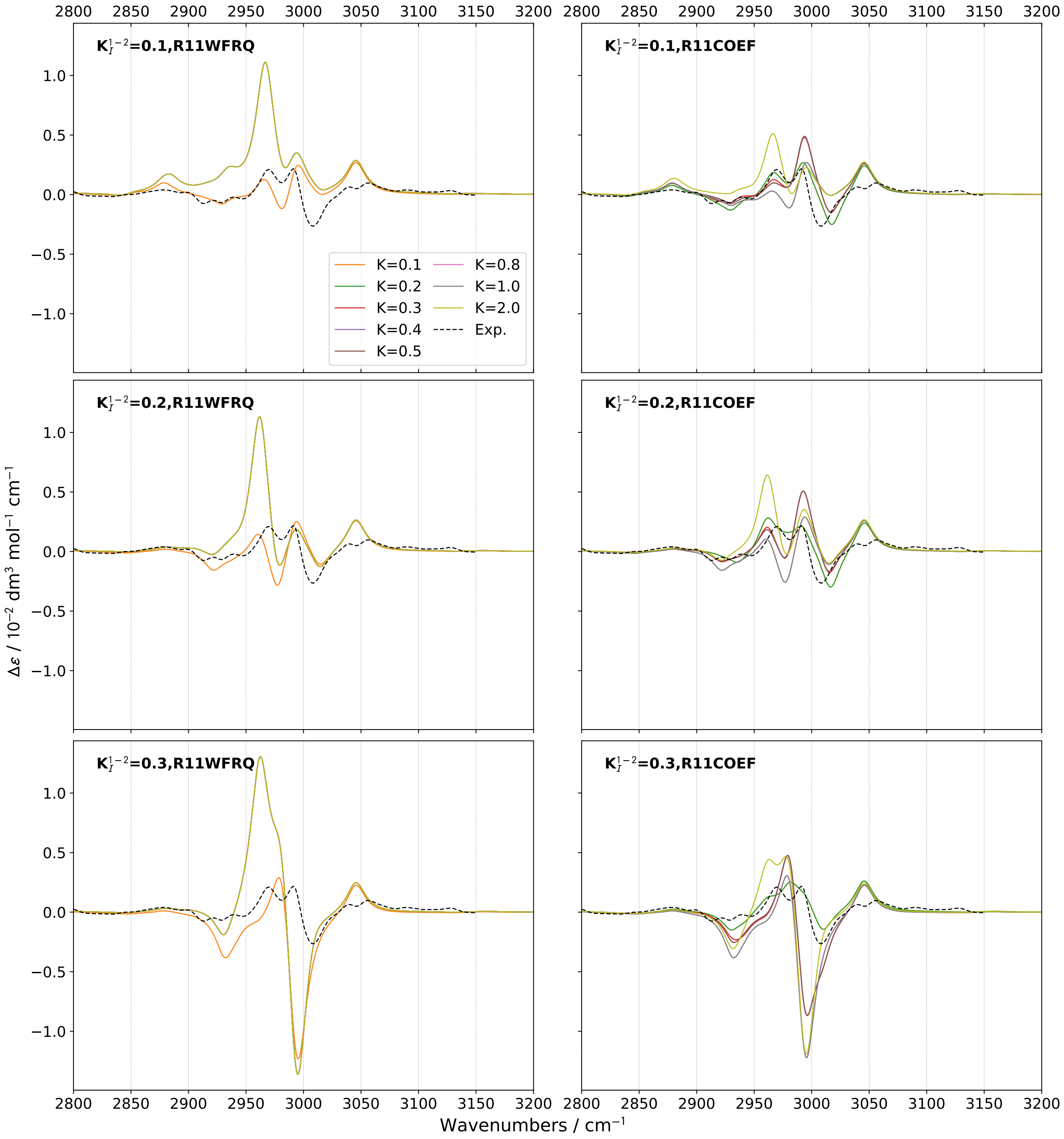

Figure S15: Comparison of theoretical GVPT2 VCD spectra of (S)-2-methyloxirane in CCl_4 within the CH-stretching region using different schemes and thresholds (K=K_I^{1-1}) for the identification of 1-1 Darling–Dennison resonances. A combination of **R12MART** (K^{1-2} = 1.0) and **R12COEF** was used for the Fermi resonances, the second test with the thresholds K_I^{1-2}=0.1 (upper panels), 0.2 (middle panels) and 0.3 (lower panels). Experiment (black dashed lines) was taken from Ref. S15. Lorentzian broadening functions with half-width at half-maximum of 10 cm^{-1} were used. The computed spectra were shifted by -22 cm^{-1} for consistency with the IR spectrum.

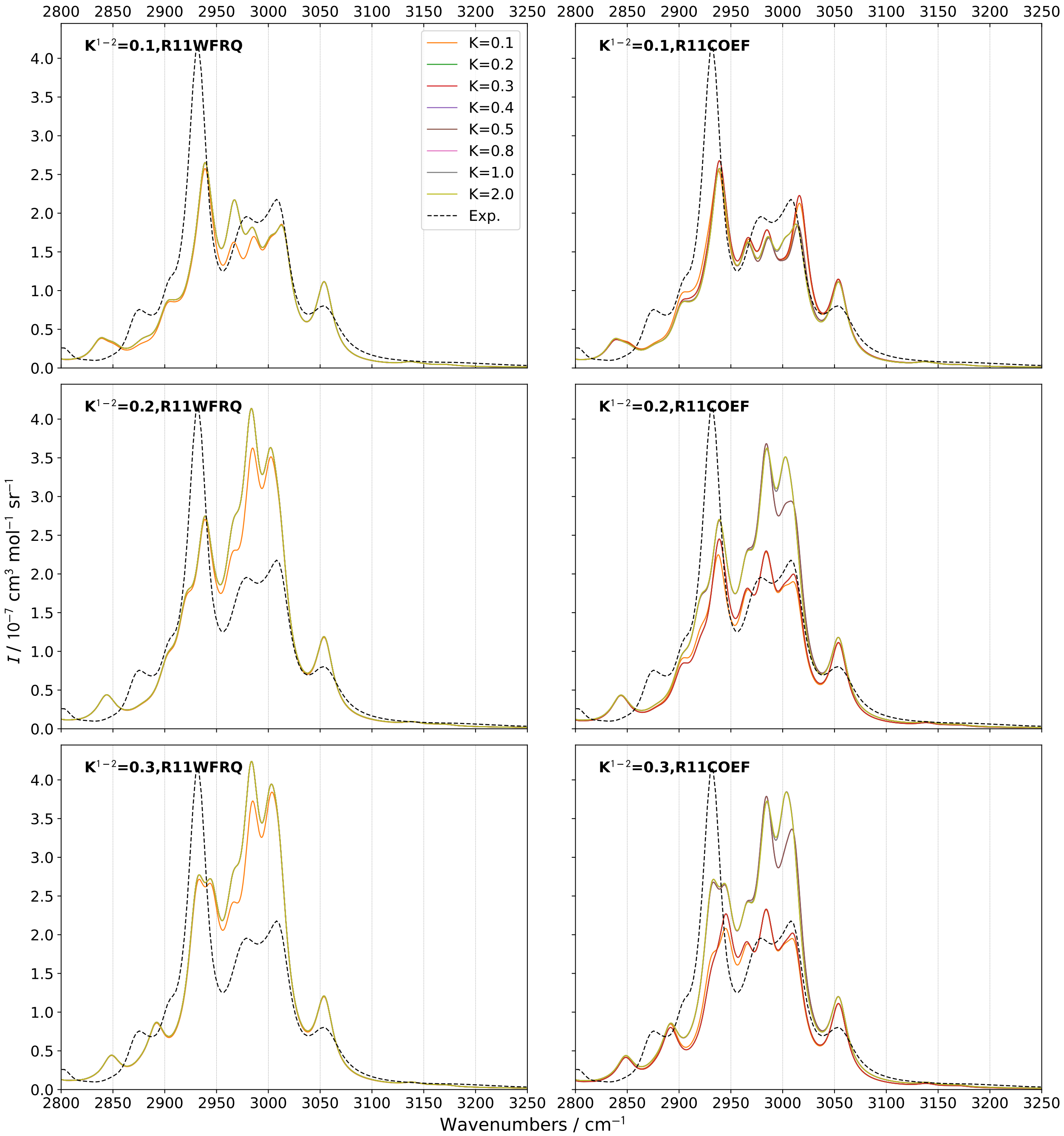

Figure S16: Comparison of theoretical GVPT2 Raman spectra of (S)-2-methyloxirane in neat liquid within the CH-stretching region using different schemes and thresholds (K=K_I^{1-1}) for the identification of 1-1 Darling–Dennison resonances. The **R12CVPT** scheme was used for the Fermi resonances, with the thresholds K_I^{1-2}=0.1 (upper panels), 0.2 (middle panels) and 0.3 (lower panels). Experiment (black dashed lines) was taken from Ref. S13. Gaussian broadening functions with half-width at half-maximum of 10 cm^{-1} were used to match experiment. The computed spectra were shifted by -18 cm^{-1} to match experiment.

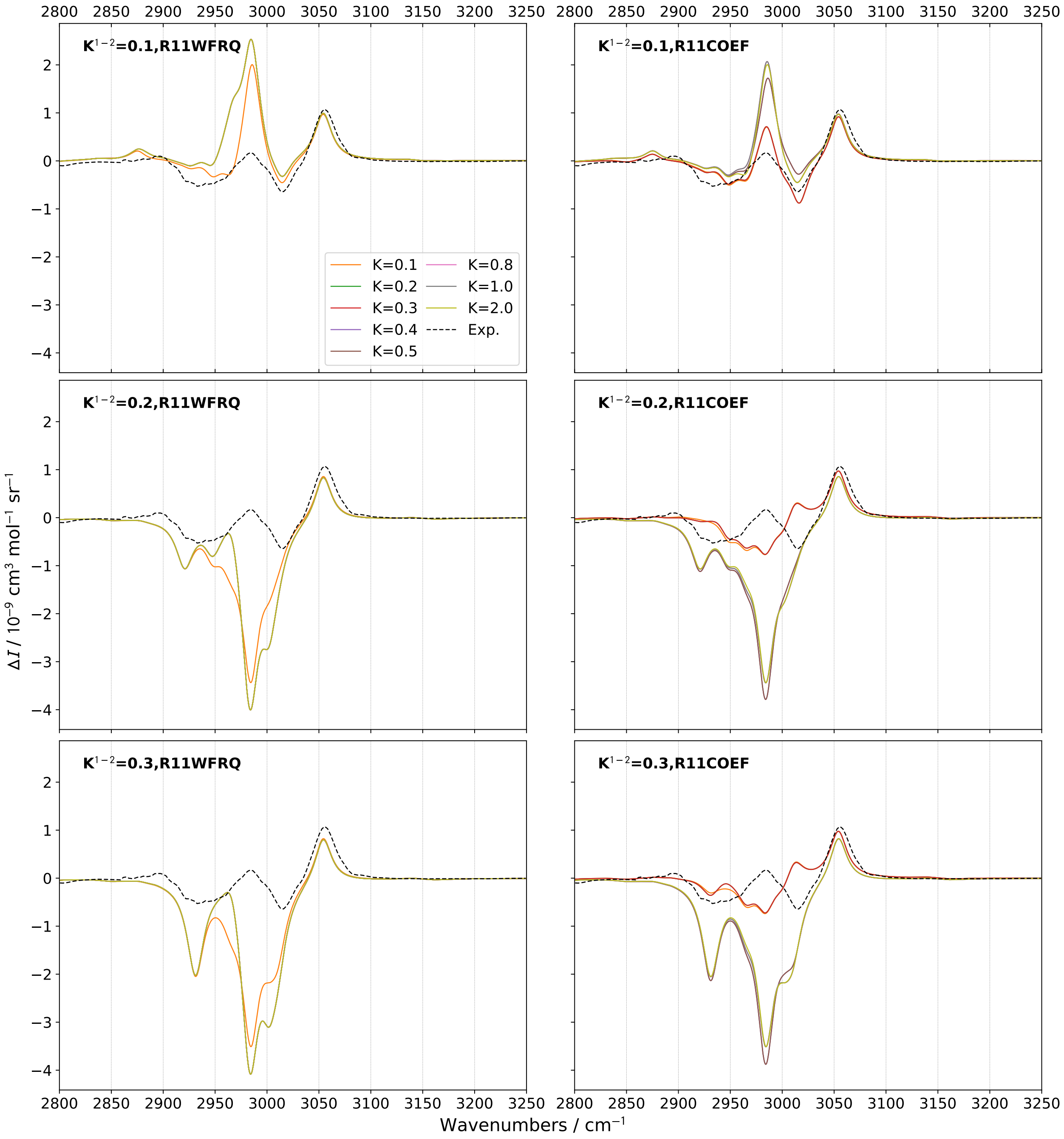

Figure S17: Comparison of theoretical GVPT2 ROA spectra of (S)-2-methyloxirane in neat liquid within the CH-stretching region using different schemes and thresholds (K=K_I^{1-1}) for the identification of 1-1 Darling–Dennison resonances. The **R12CVPT** scheme was used for the Fermi resonances, with the thresholds K_I^{1-2}=0.1 (upper panels), 0.2 (middle panels) and 0.3 (lower panels). Experiment (black dashed lines) was taken from Ref. S13. Gaussian broadening functions with half-width at half-maximum of 10 cm^{-1} were used to match experiment. The computed spectra were shifted by -18 cm^{-1} to match experiment.

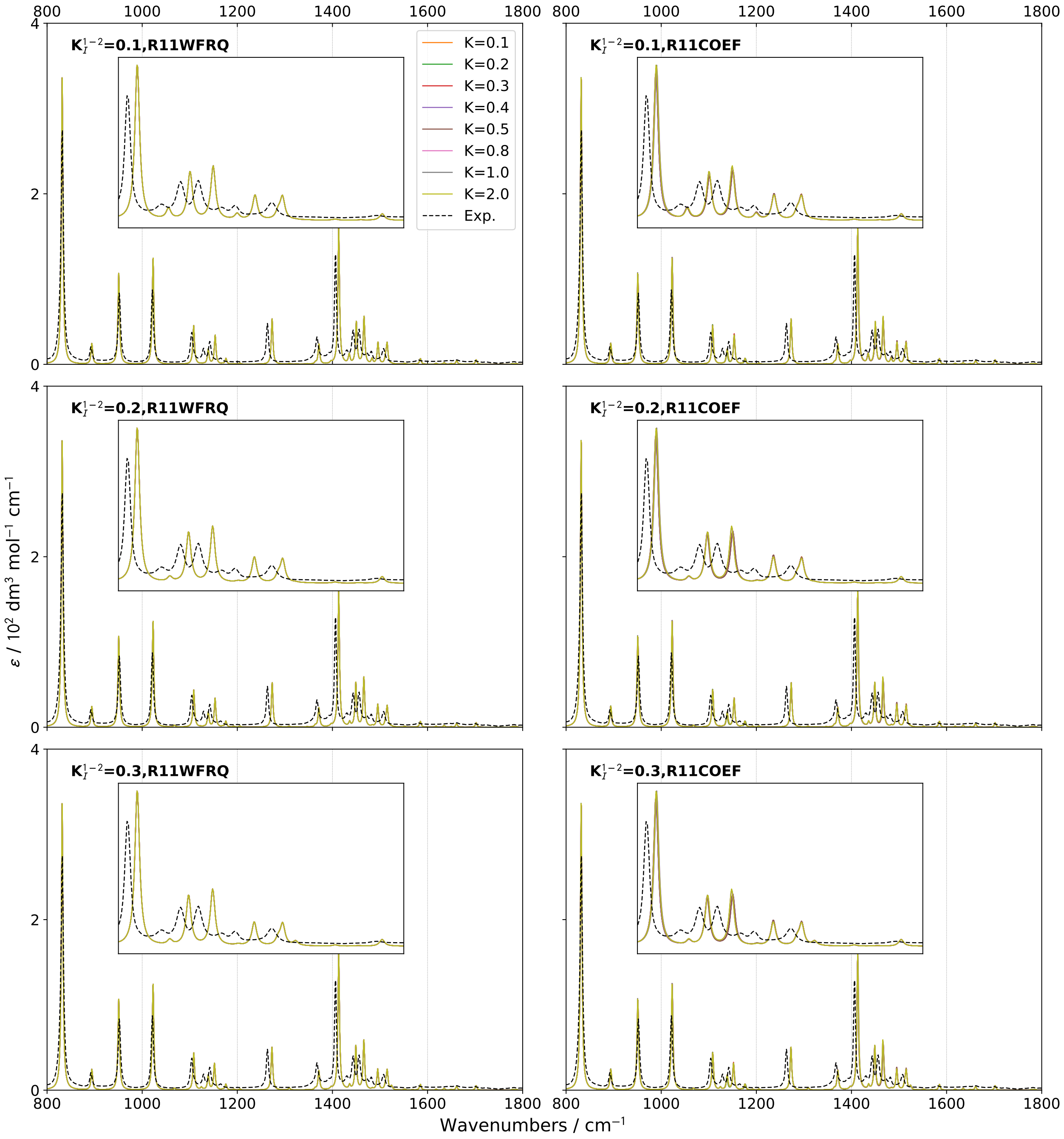

Figure S18: Comparison of theoretical GVPT2 IR spectra of (S)-2-methyloxirane in liquid xenon within the fingerprint region using different schemes and thresholds (K=K_I^{1-1}) for the identification of 1-1 Darling–Dennison resonances. A combination of **R12MART** (K^{1-2} = 1.0) and **R12COEF** was used for the Fermi resonances, the second test with the thresholds K_I^{1-2}=0.1 (upper panels), 0.2 (middle panels) and 0.3 (lower panels). Experiment (black dashed lines) was taken from Ref. S14. Lorentzian broadening functions with half-width at half-maximum of 2 cm^{-1} were used to match experiment. The inset shows a zoom of the 1400–1600 cm^{-1} region.

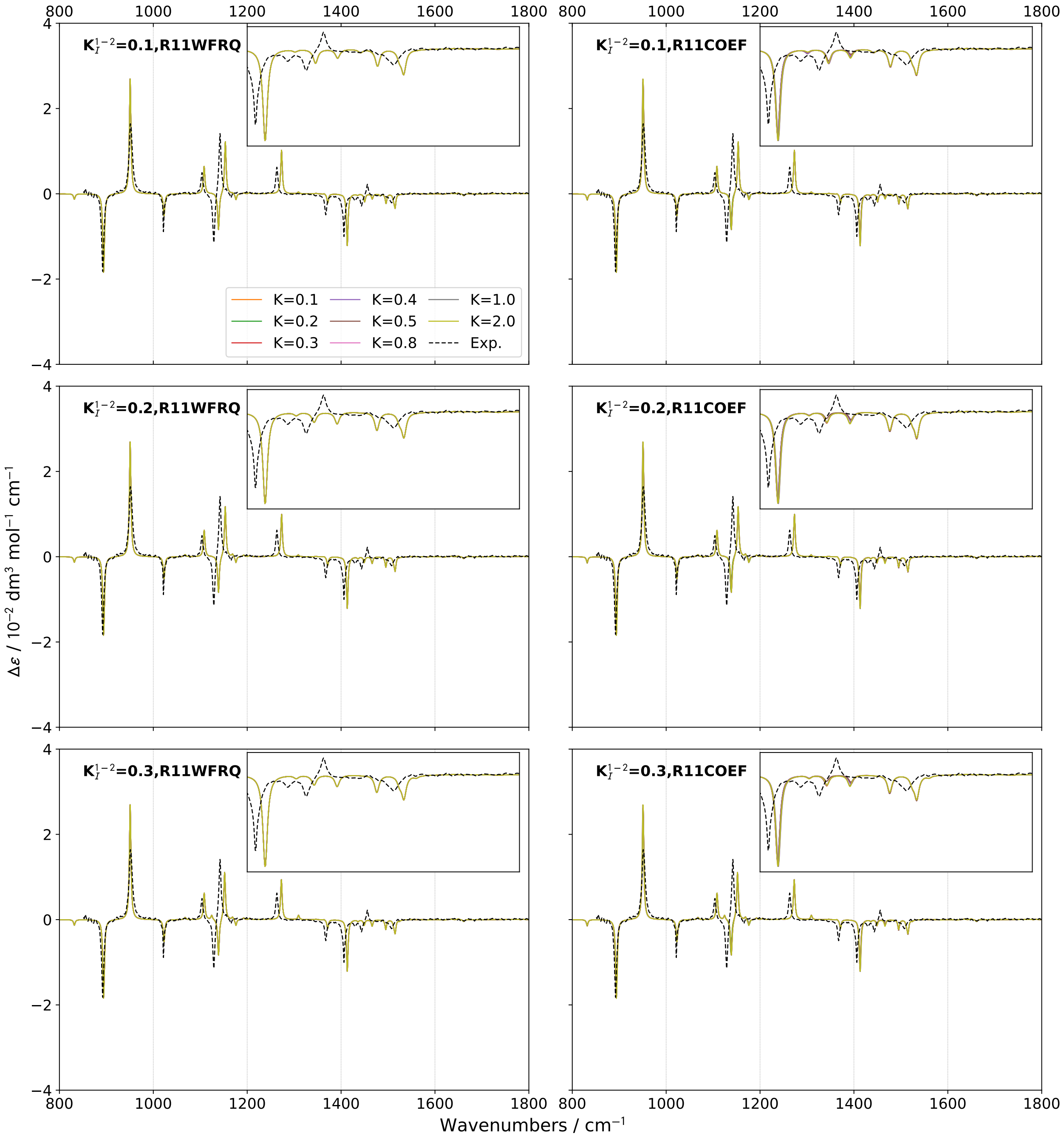

Figure S19: Comparison of theoretical GVPT2 VCD spectra of (S)-2-methyloxirane in liquid xenon within the fingerprint region using different schemes and thresholds (K=K_I^{1-1}) for the identification of 1-1 Darling–Dennison resonances. A combination of **R12MART** ($K^{1-2} = 1.0$) and **R12COEF** was used for the Fermi resonances, the second test with the thresholds K_I^{1-2}=0.1 (upper panels), 0.2 (middle panels) and 0.3 (lower panels). Experiment (black dashed lines) was taken from Ref. S14. Lorentzian broadening functions with half-width at half-maximum of 2 cm^{-1} were used to match experiment. The inset shows a zoom of the 1400–1600 cm^{-1} region.

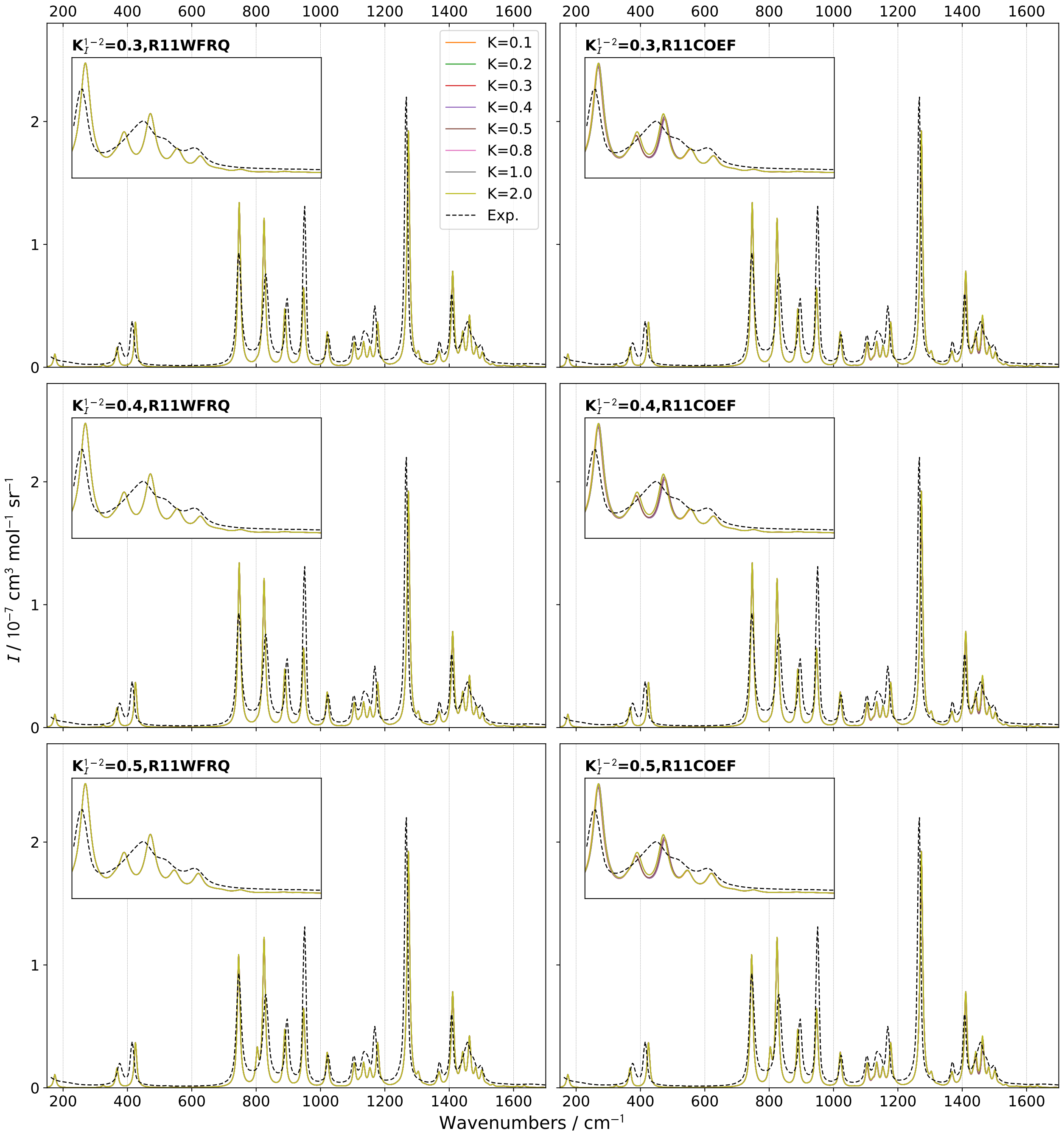

Figure S20: Comparison of theoretical GVPT2 Raman spectra of (S)-2-methyloxirane in neat liquid within the fingerprint region using different schemes and thresholds (K=K_I^{1-1}) for the identification of 1-1 Darling–Dennison resonances. A combination of **R12MART** ($K^{1-2} = 1.0$) and **R12COEF** was used for the Fermi resonances, the second test with the thresholds K_I^{1-2}=0.1 (upper panels), 0.2 (middle panels) and 0.3 (lower panels). Experiment (black dashed lines) was taken from Ref. S13. Gaussian broadening functions with half-width at half-maximum of 5 cm^{-1} were used to match experiment. The inset shows a zoom of the 1400–1600 cm^{-1} region.

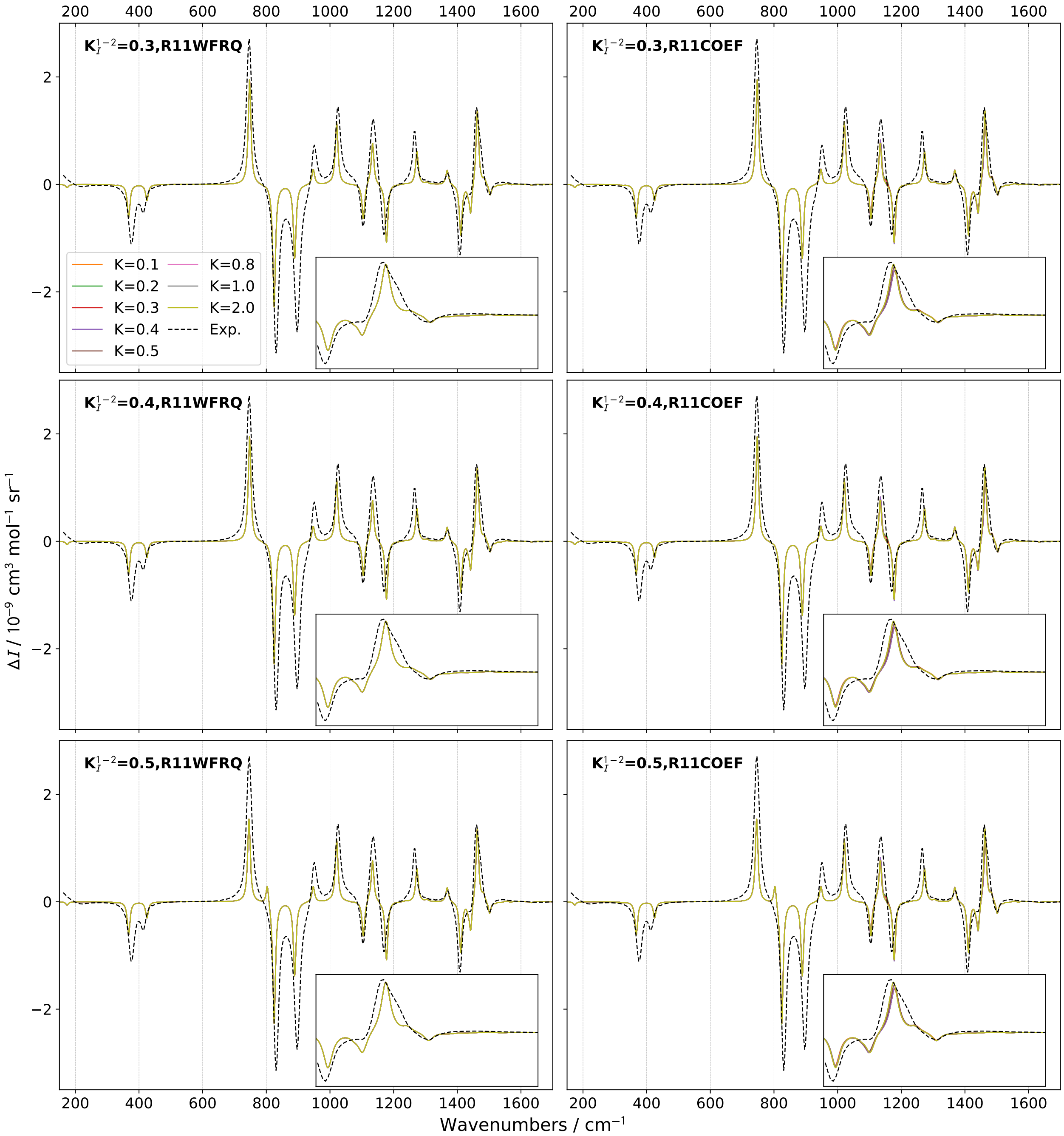

Figure S21: Comparison of theoretical GVPT2 ROA spectra of (S)-2-methyloxirane in neat liquid within the fingerprint region using different schemes and thresholds (K=K_I^{1-1}) for the identification of 1-1 Darling–Dennison resonances. A combination of **R12MART** ($K^{1-2} = 1.0$) and **R12COEF** was used for the Fermi resonances, the second test with the thresholds K_I^{1-2}=0.1 (upper panels), 0.2 (middle panels) and 0.3 (lower panels). Experiment (black dashed lines) was taken from Ref. S13. Gaussian broadening functions with half-width at half-maximum of 5 cm^{-1} were used to match experiment. The inset shows a zoom of the 1400–1600 cm^{-1} region.

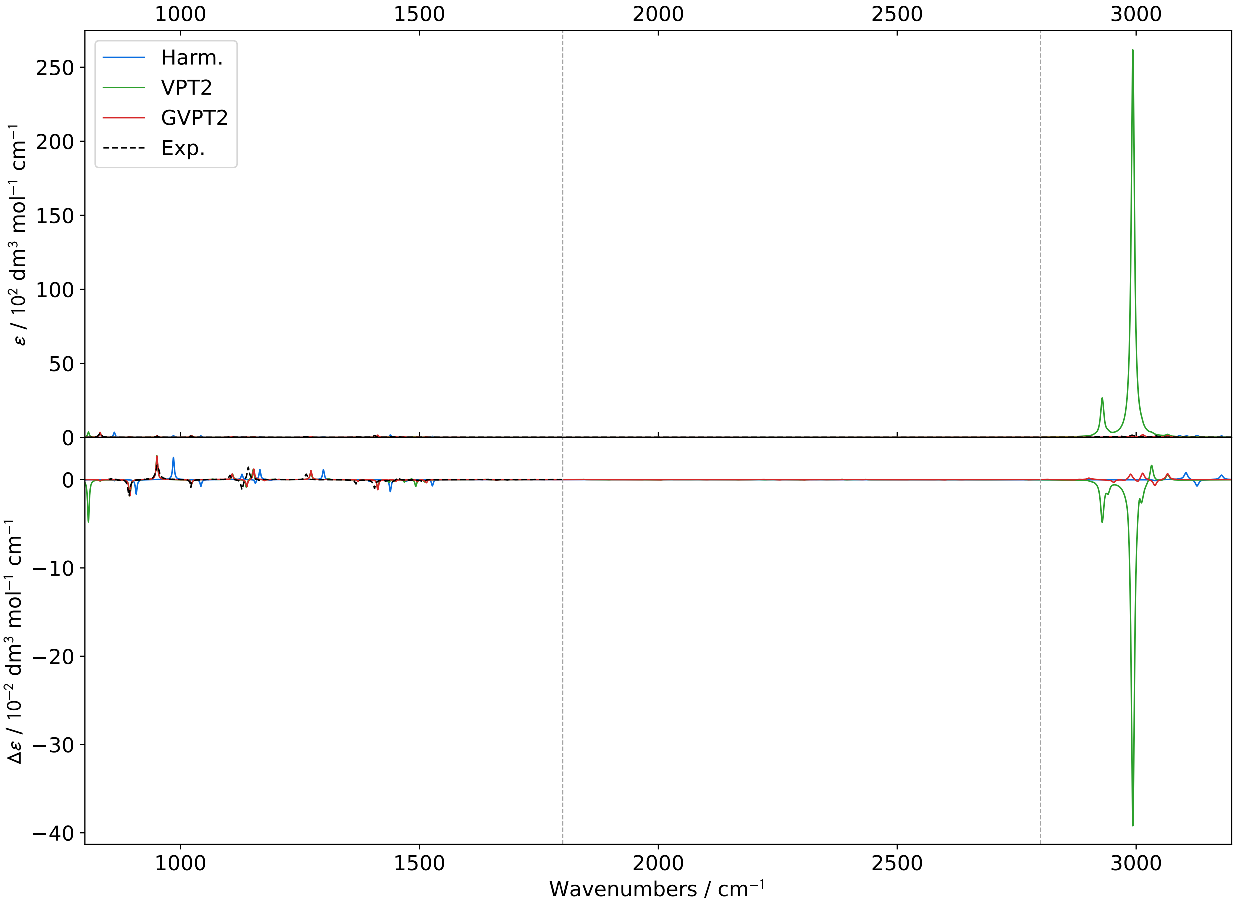

Figure S22: Experimental (black dashed lines),[S14] harmonic (blue), VPT2 (green) and GVPT2 (red) IR and VCD spectra of (S)-2-methyloxirane in liquid xenon. The automatic procedure used a combination of **R12MART**/**R12COEF** ($\Delta^{1-2} = 200$ / $K^{1-2} = 1.0$ / $K_I^{1-2} = 0.1$) for FRs, **R11HRS**/**R11COEF** ($\Delta^{1-1} = 100$ / $K^{1-1} = 10$ / $K_I^{1-1} = 0.3$) for 1-1 DDRs, **R22HRS** ($\Delta^{2-2} = 100$ / $K^{2-2} = 20$) for 2-2 DDRs. Lorentzian broadening functions with half-width at half-maximum of 2 cm^{-1} were used to match experiment, except in the CH-stretching region, where 4 cm^{-1} was used.

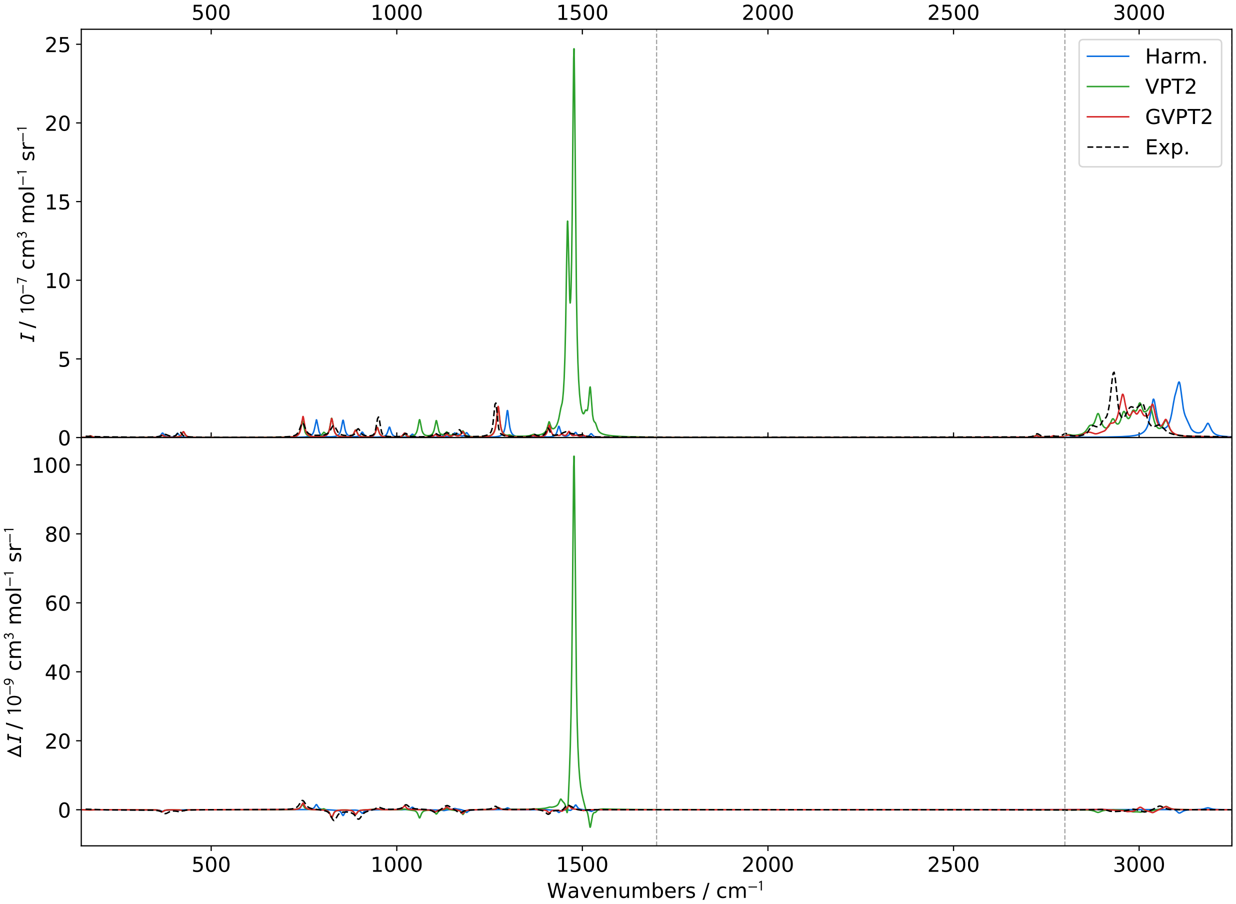

Figure S23: Experimental (black dashed lines),[S13] harmonic (blue), VPT2 (green) and GVPT2 (red) Raman and ROA spectra of (S)-2-methyloxirane in neat liquid. The automatic procedure used a combination of **R12MART**/**R12COEF** ($\Delta^{1-2} = 200$ / $K^{1-2} = 1.0$ / $K_I^{1-2} = 0.1$) for FRs, **R11HRS**/**R11COEF** ($\Delta^{1-1} = 100$ / $K^{1-1} = 10$ / $K_I^{1-1} = 0.3$) for 1-1 DDRs, **R22HRS** ($\Delta^{2-2} = 100$ / $K^{2-2} = 20$) for 2-2 DDRs. Gaussian broadening functions with half-width at half-maximum of 5 cm^{-1} were used to match experiment, except in the CH-stretching region, where 10 cm^{-1} was used.

Additional figures on pinene

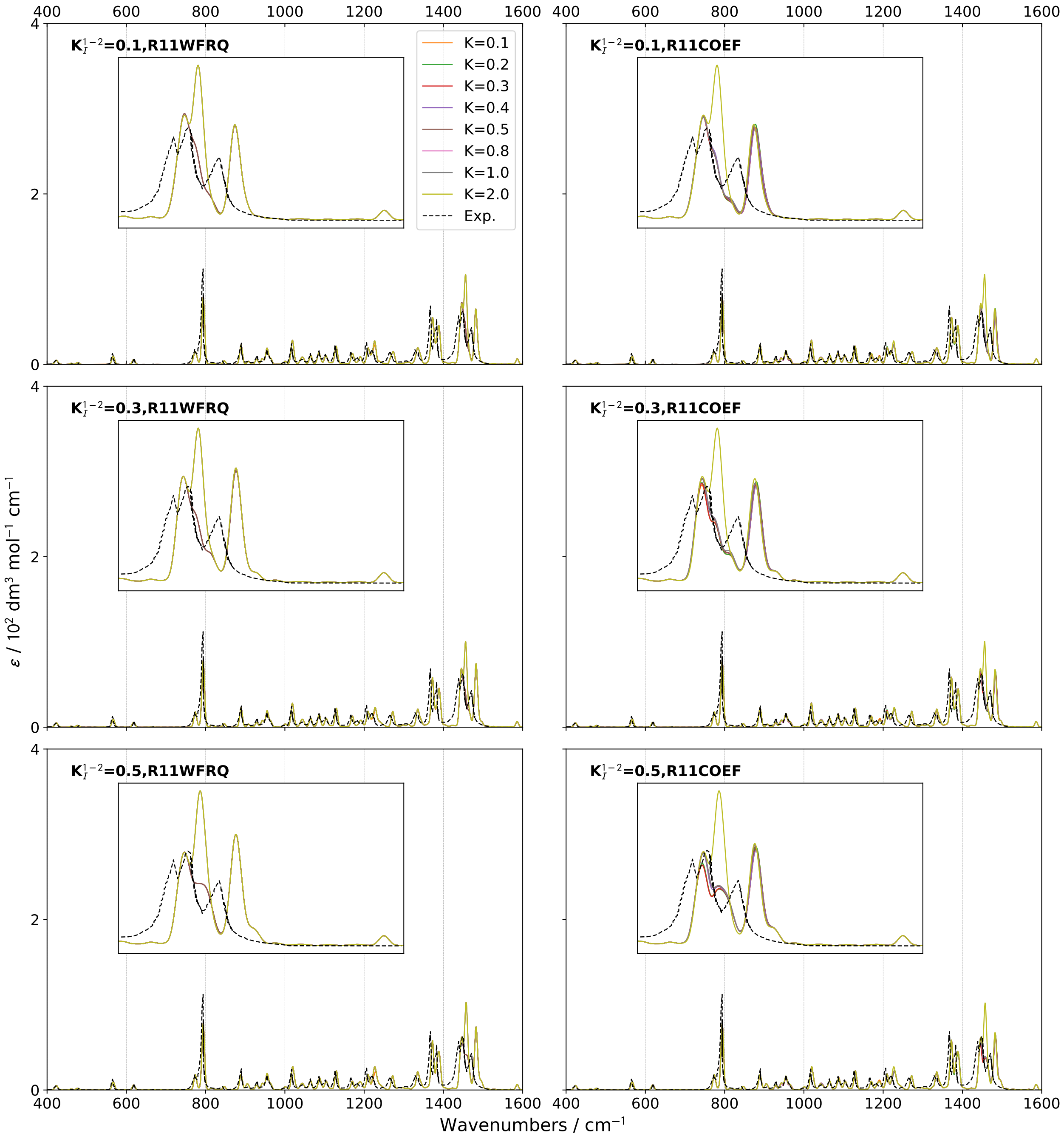

Figure S24: Comparison of theoretical GVPT2 IR spectra of $(1R,5R)$-α-pinene in CCl_4 within the fingerprint region using different schemes and thresholds (K=K_I^{1-1}) for the identification of 1-1 Darling–Dennison resonances. A combination of **R12MART** ($K^{1-2} = 1.0$) and **R12COEF** was used for the Fermi resonances, the second test with the thresholds K_I^{1-2}=0.1 (upper panels), 0.3 (middle panels) and 0.5 (lower panels). Experiment (black dashed lines) was taken from Ref. S16. Lorentzian broadening functions with half-width at half-maximum of 4 cm^{-1} were used to match experiment.

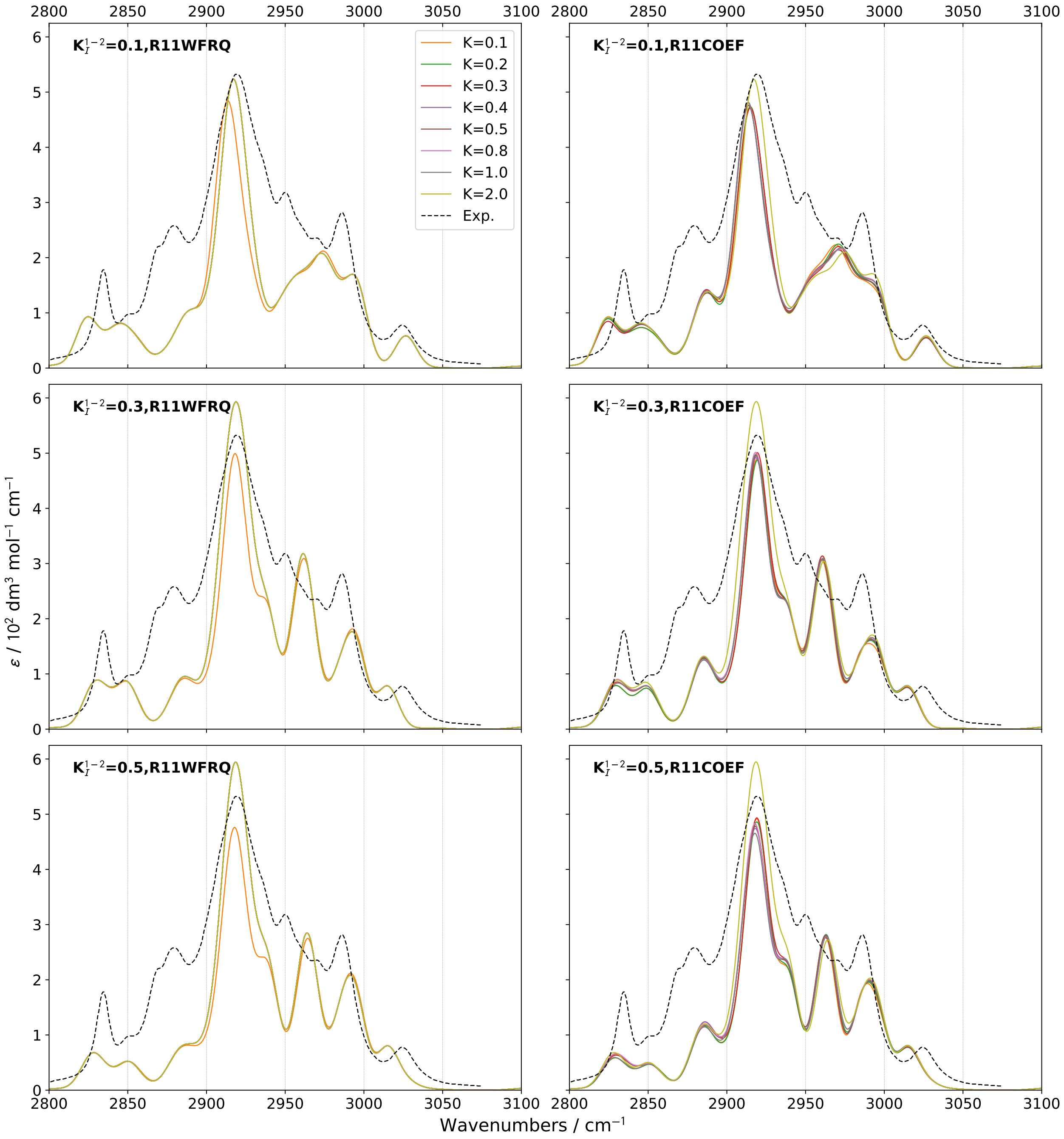

Figure S25: Comparison of theoretical GVPT2 IR spectra of (1R,5R)-α-pinene in CCl_4 within the CH-stretching region using different schemes and thresholds (K=K_I^{1-1}) for the identification of 1-1 Darling–Dennison resonances. A combination of **R12MART** (K^{1-2} = 1.0) and **R12COEF** was used for the Fermi resonances, the second test with the thresholds K_I^{1-2}=0.1 (upper panels), 0.3 (middle panels) and 0.5 (lower panels). Experiment (black dashed lines) was taken from Ref. S16. Lorentzian broadening functions with half-width at half-maximum of 8 cm^{-1} were used to match experiment. The computed spectra were shifted by -20 cm^{-1} to match experiment.

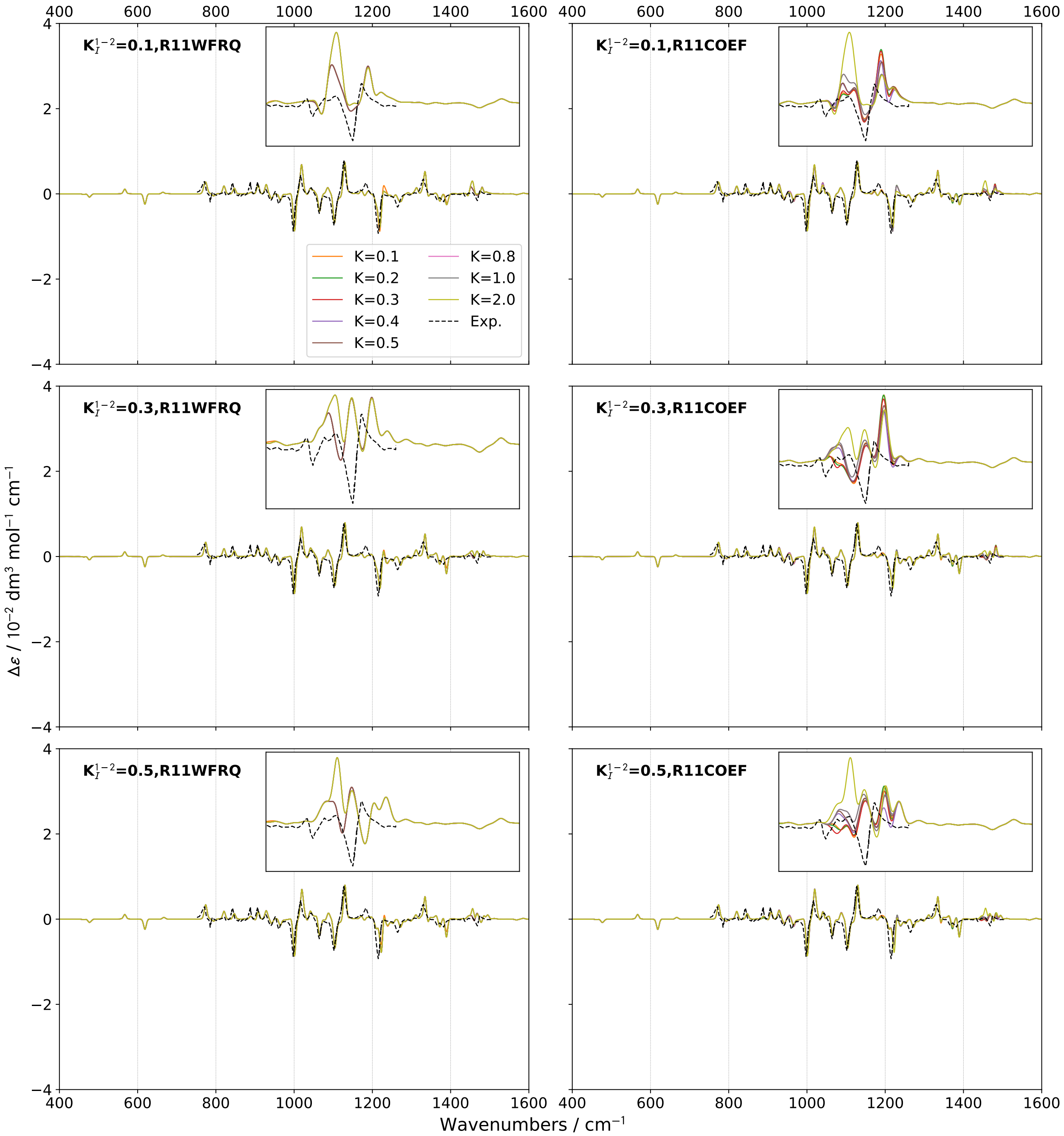

Figure S26: Comparison of theoretical GVPT2 VCD spectra of (1R,5R)-α-pinene in CCl_4 within the fingerprint region using different schemes and thresholds (K=K_I^{1-1}) for the identification of 1-1 Darling–Dennison resonances. A combination of **R12MART** ($K^{1-2} = 1.0$) and **R12COEF** was used for the Fermi resonances, the second test with the thresholds K_I^{1-2}=0.1 (upper panels), 0.3 (middle panels) and 0.5 (lower panels). Experiment (black dashed lines) was taken from Ref. S16. Lorentzian broadening functions with half-width at half-maximum of 4 cm^{-1} were used to match experiment.

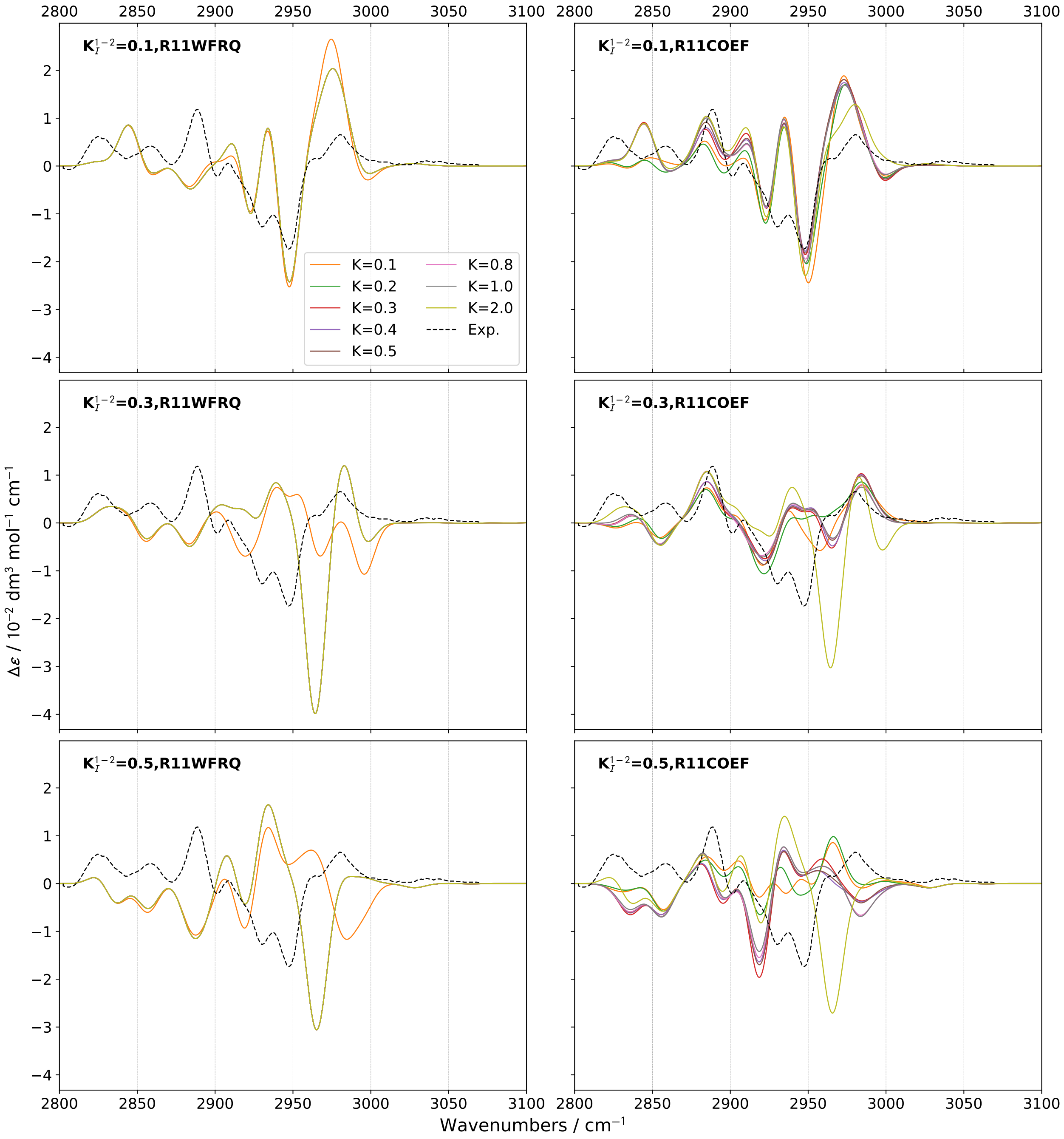

Figure S27: Comparison of theoretical GVPT2 VCD spectra of (1R,5R)-α-pinene in CCl_4 within the CH-stretching region using different schemes and thresholds (K=K_I^{1-1}) for the identification of 1-1 Darling–Dennison resonances. A combination of **R12MART** (K^{1-2} = 1.0) and **R12COEF** was used for the Fermi resonances, the second test with the thresholds K_I^{1-2}=0.1 (upper panels), 0.3 (middle panels) and 0.5 (lower panels). Experiment (black dashed lines) was taken from Ref. S16. Lorentzian broadening functions with half-width at half-maximum of 8 cm^{-1} were used to match experiment. The computed spectra were shifted by -20 cm^{-1} to match experiment.

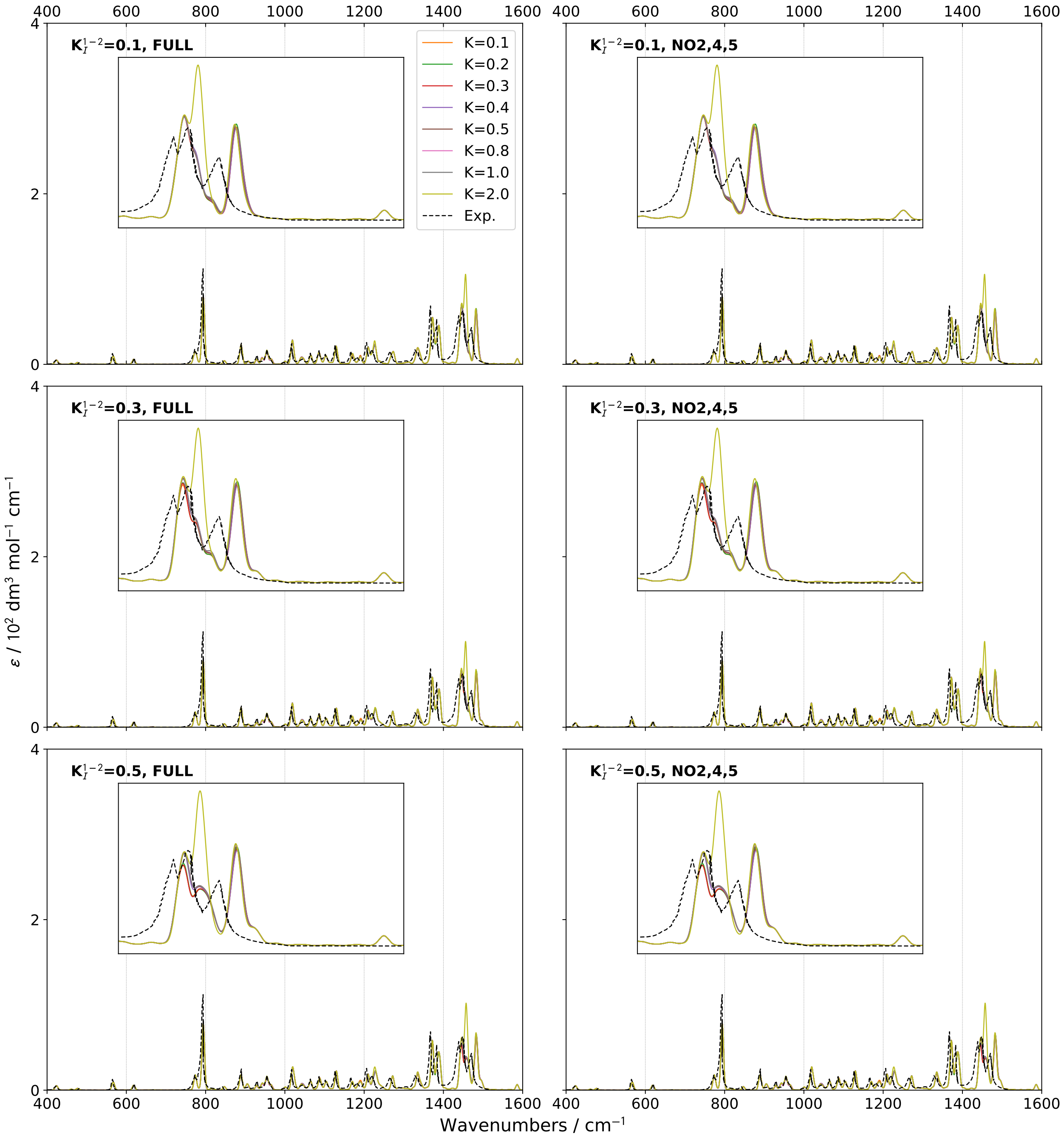

Figure S28: Comparison of theoretical GVPT2 IR spectra of (1R,5R)-α-pinene in CCl_4 within the fingerprint region considering the whole system ("FULL") or excluding the torsional modes related to the methyl groups ("NO2,4,5") with different thresholds (K=K_I^{1-1}) for the identification of 1-1 Darling–Dennison resonances. A combination of **R12MART** ($K^{1-2} = 1.0$) and **R12COEF** was used for the Fermi resonances, the second test with the thresholds K_I^{1-2}=0.1 (upper panels), 0.3 (middle panels) and 0.5 (lower panels). Experiment (black dashed lines) was taken from Ref. S16. Lorentzian broadening functions with half-width at half-maximum of 4 cm^{-1} were used to match experiment.

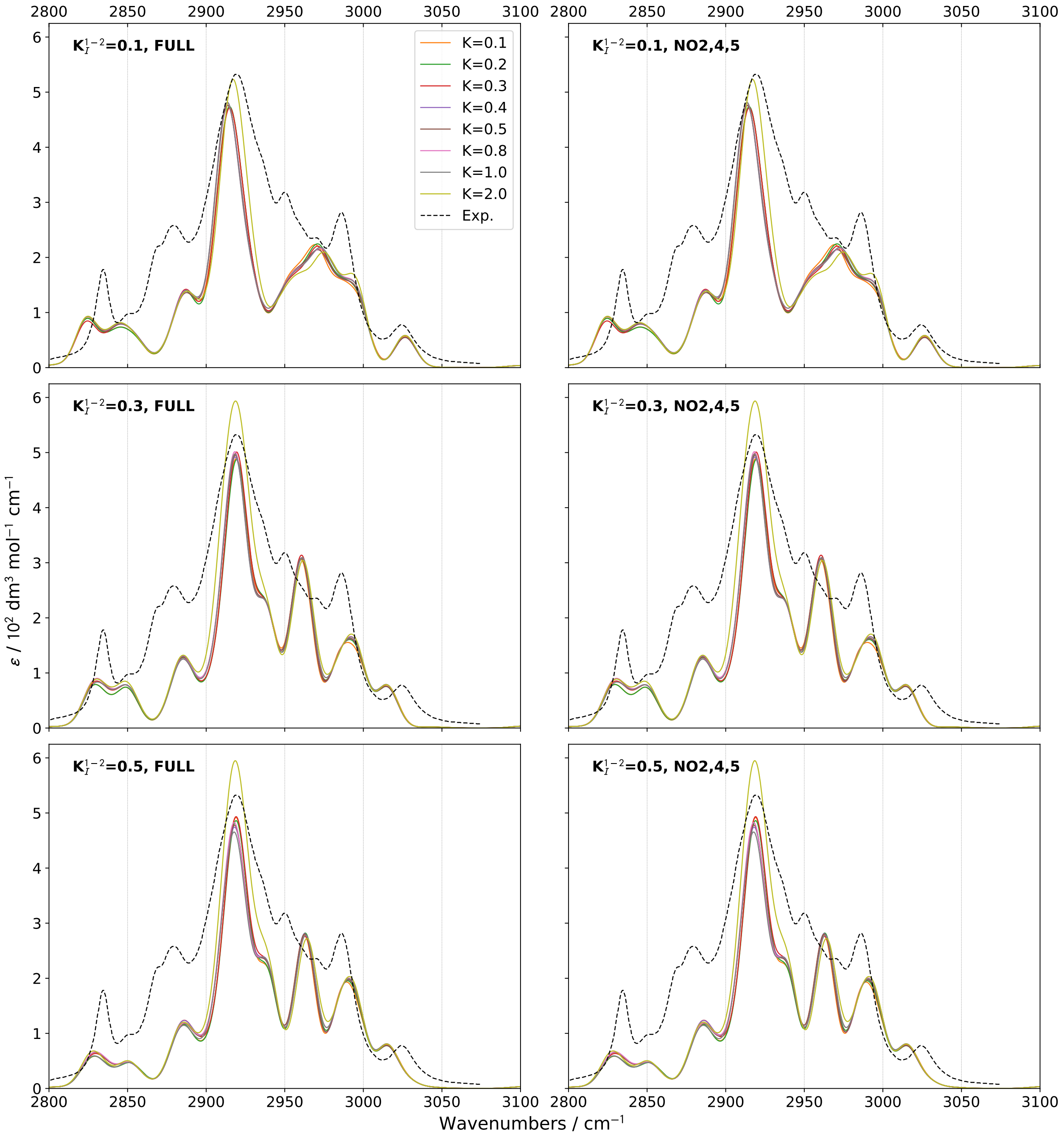

Figure S29: Comparison of theoretical GVPT2 IR spectra of (1R,5R)-α-pinene in CCl_4 within the CH-stretching region considering the whole system ("FULL") or excluding the torsional modes related to the methyl groups ("NO2,4,5") with different thresholds (K=K_I^{1-1}) for the identification of 1-1 Darling–Dennison resonances. A combination of **R12MART** ($K^{1-2} = 1.0$) and **R12COEF** was used for the Fermi resonances, the second test with the thresholds K_I^{1-2}=0.1 (upper panels), 0.3 (middle panels) and 0.5 (lower panels). Experiment (black dashed lines) was taken from Ref. S16. Lorentzian broadening functions with half-width at half-maximum of 8 cm^{-1} were used to match experiment. The computed spectra were shifted by -20 cm^{-1} to match experiment.

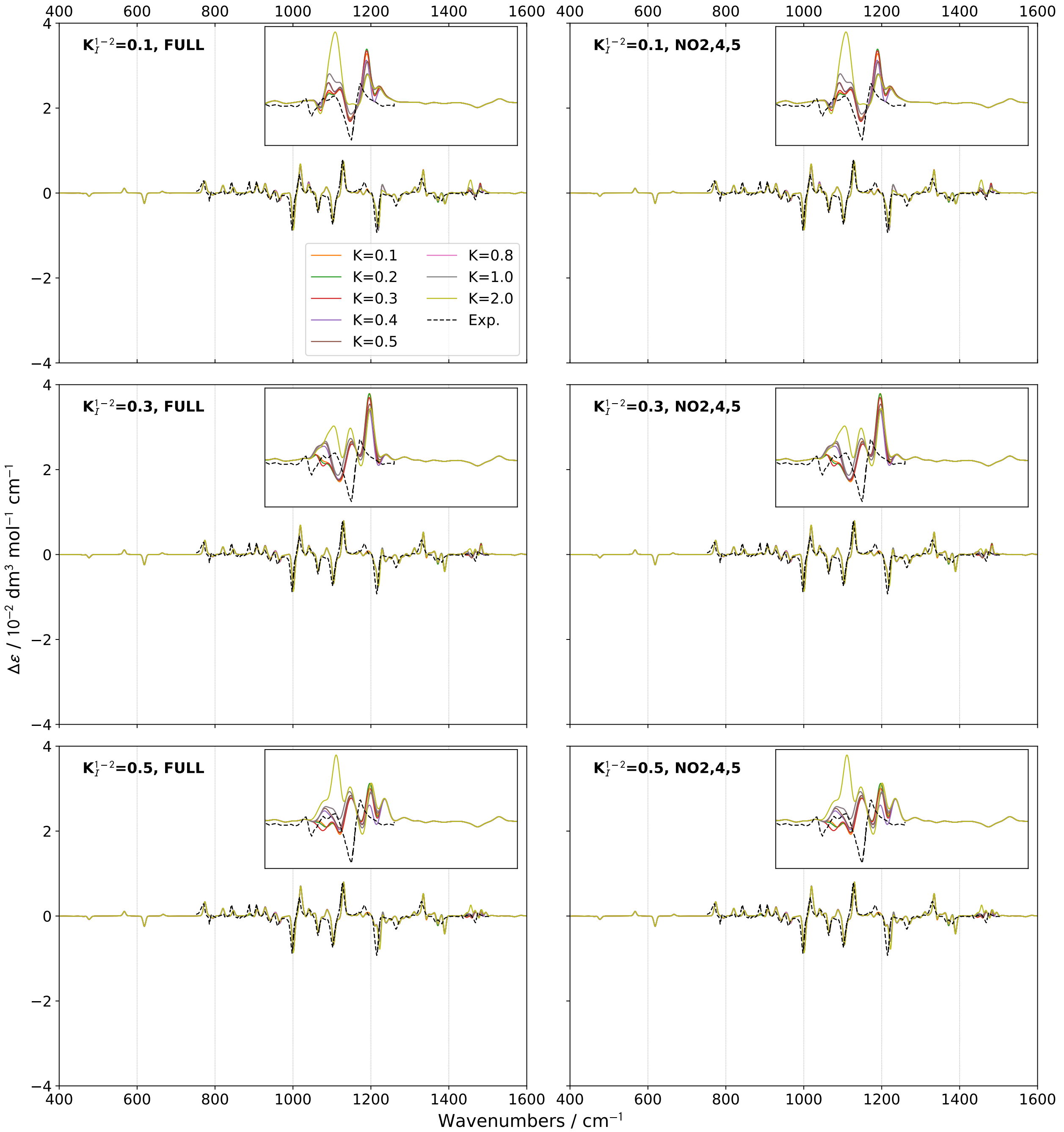

Figure S30: Comparison of theoretical GVPT2 VCD spectra of (1R,5R)-α-pinene in CCl_4 within the fingerprint region considering thee all system ("FULL") or excluding the torsional modes related to the methyl groups ("NO2,4,5") with different thresholds (K=K_I^{1-1}) for the identification of 1-1 Darling–Dennison resonances. A combination of **R12MART** ($K^{1-2} = 1.0$) and **R12COEF** was used for the Fermi resonances, the second test with the thresholds K_I^{1-2}=0.1 (upper panels), 0.3 (middle panels) and 0.5 (lower panels). Experiment (black dashed lines) was taken from Ref. S16. Lorentzian broadening functions with half-width at half-maximum of 4 cm^{-1} were used to match experiment.

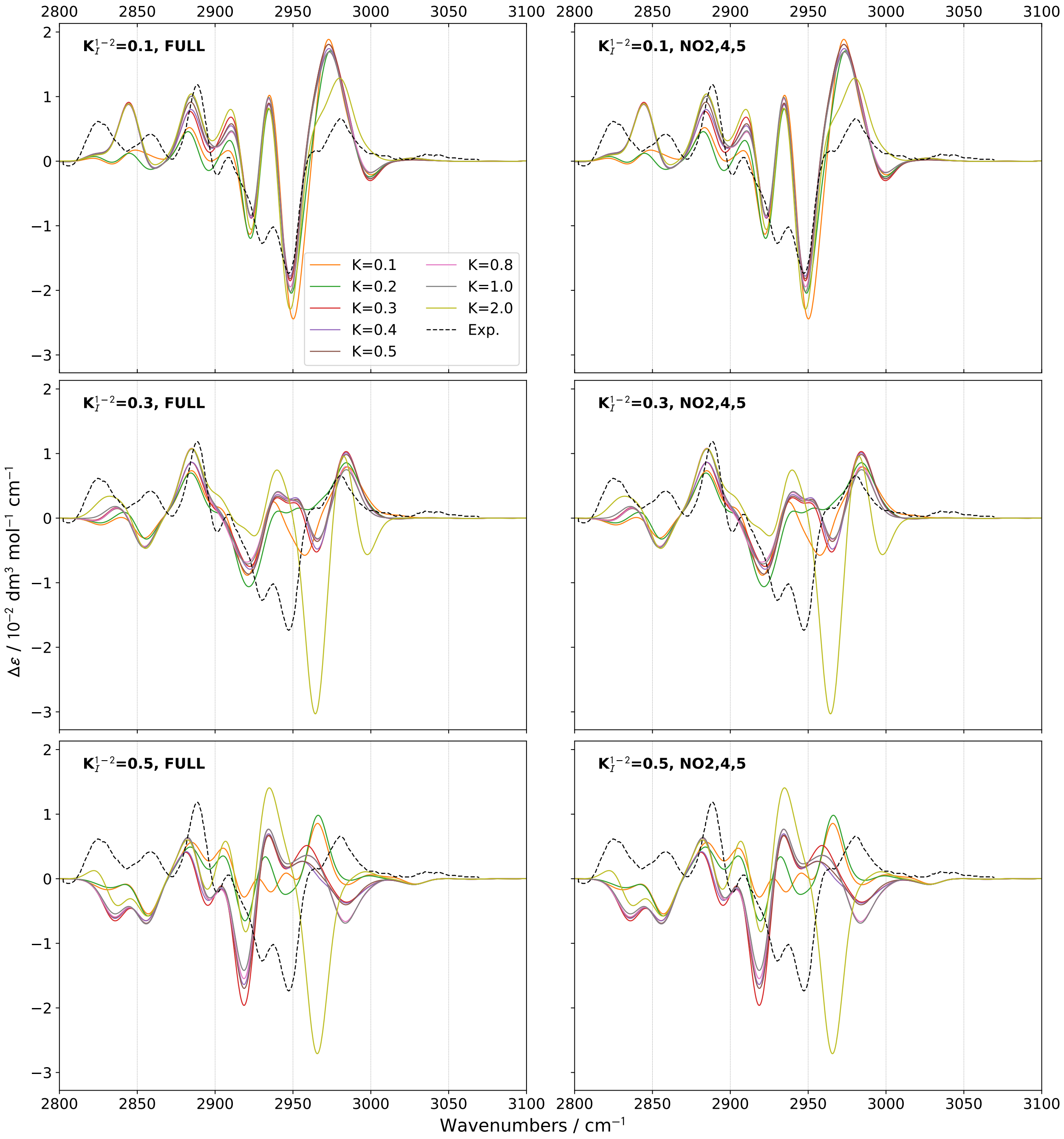

Figure S31: Comparison of theoretical GVPT2 VCD spectra of (1R,5R)-α-pinene in CCl_4 within the CH-stretching region considering thee all system ("FULL") or excluding the torsional modes related to the methyl groups ("NO2,4,5") with different thresholds (K=K_I^{1-1}) for the identification of 1-1 Darling–Dennison resonances. A combination of **R12MART** (K^{1-2} = 1.0) and **R12COEF** was used for the Fermi resonances, the second test with the thresholds K_I^{1-2}=0.1 (upper panels), 0.3 (middle panels) and 0.5 (lower panels). Experiment (black dashed lines) was taken from Ref. S16. Lorentzian broadening functions with half-width at half-maximum of 8 cm^{-1} were used to match experiment. The computed spectra were shifted by -20 cm^{-1} to match experiment.

Additional figures on artemisinin

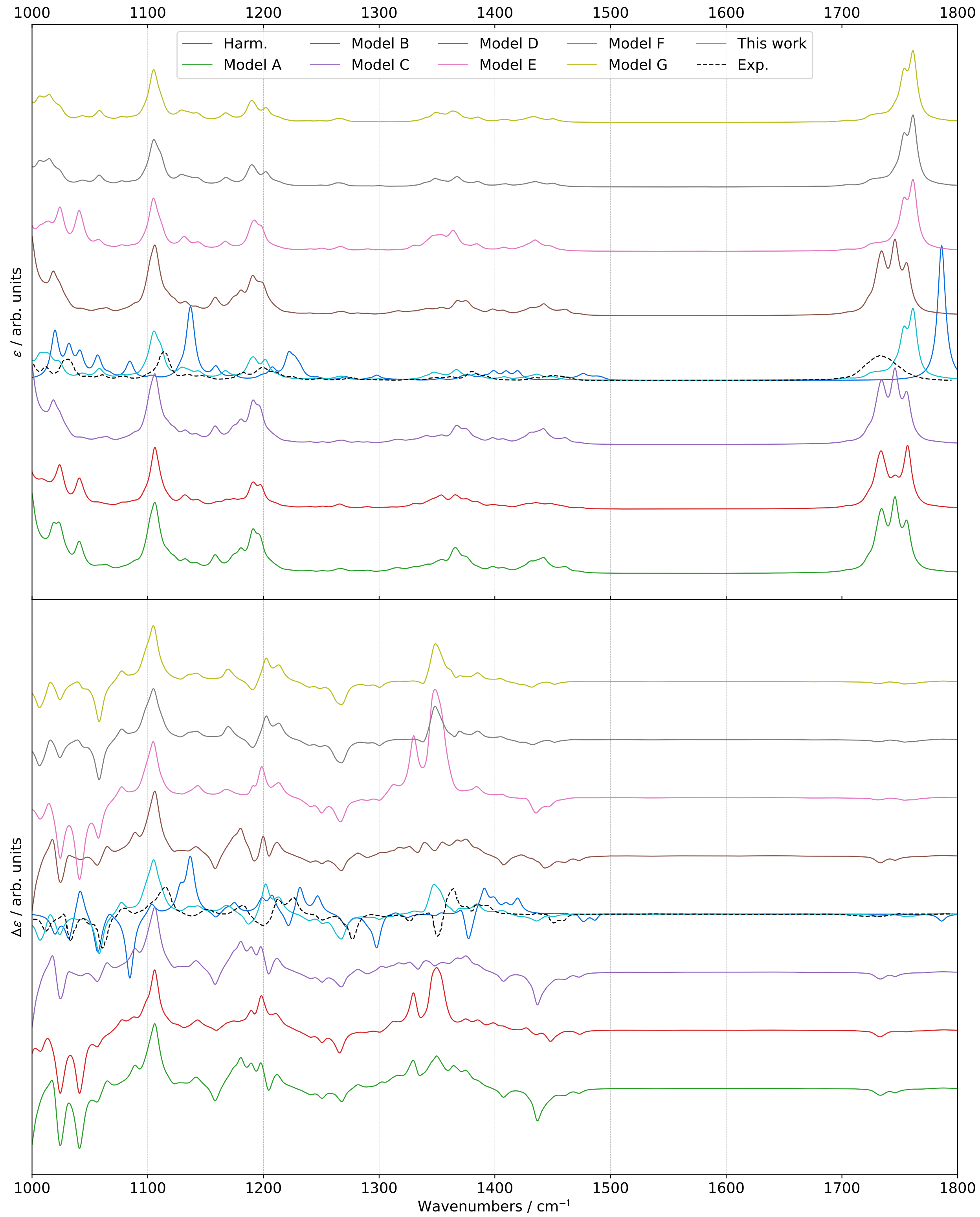

Figure S32: Experimental (black dashed lines)[S17] and GVPT2 IR (upper panels) and VCD (lower panels) spectra of artemisinin in chloroform. The models are described in the main text. Lorentzian broadening functions with half-width at half-maximum of 4 cm^{-1} were used to match experiment.

References

(S1) Borro, A. F.; Mills, I. M.; Venuti, E. Quartic anharmonic resonances in acetylenes and haloacetylenes. *J. Chem. Phys.* **1995**, *102*, 3938–3944.

(S2) Martin, J. M. L.; Taylor, P. M. Accurate ab initio quartic force field for trans-HNNH and treatment of resonance polyads. *Spectrochim. Acta A* **1997**, *53*, 1039–1050.

(S3) McKean, D. C.; Veken, B. v. d.; Herrebout, W.; Law, M. M.; Brenner, M. J.; Nemchick, D. J.; Craig, N. C. Infrared Spectra of $^{12}CF_2{=}^{12}CH_2$ and $^{12}CF_2{=}^{13}CH_2$, Quantum-Chemical Calculations of Anharmonicity, and Analyses of Resonances. *J. Phys. Chem. A* **2010**, *114*, 5728–5742.

(S4) Rosnik, A. M.; Polik, W. F. VPT2+K spectroscopic constants and matrix elements of the transformed vibrational Hamiltonian of a polyatomic molecule with resonances using Van Vleck perturbation theory. *Mol. Phys.* **2014**, *112*, 261–300.

(S5) Krasnoshchekov, S. V.; Isayeva, E. V.; Stepanov, N. F. Criteria for first- and second-order vibrational resonances and correct evaluation of the Darling-Dennison resonance coefficients using the canonical Van Vleck perturbation theory. *J. Chem. Phys.* **2014**, *141*, 234114.

(S6) Kuhler, K. M.; Truhlar, D. G.; Isaacson, A. D. General method for removing resonance singularities in quantum mechanical perturbation theory. *J. Chem. Phys.* **1996**, *104*, 4664–4670.

(S7) Bloino, J.; Biczysko, M.; Barone, V. General Perturbative Approach for Spectroscopy, Thermodynamics, and Kinetics: Methodological Background and Benchmark Studies. *J. Chem. Theory Comput.* **2012**, *8*, 1015–1036.

(S8) Bloino, J.; Baiardi, A.; Biczysko, M. Aiming at an accurate prediction of vibrational

and electronic spectra for medium-to-large molecules: An overview. *Int. J. Quantum Chem.* **2016**, *116*, 1543–1574.

(S9) Bloino, J. A VPT2 Route to Near-Infrared Spectroscopy: The Role of Mechanical and Electrical Anharmonicity. *J. Phys. Chem. A* **2015**, *119*, 5269–5287.

(S10) Franke, P. R.; Stanton, J. F.; Douberly, G. E. How to VPT2: Accurate and Intuitive Simulations of CH Stretching Infrared Spectra Using VPT2+K with Large Effective Hamiltonian Resonance Treatments. *J. Phys. Chem. A* **2021**, *125*, 1301–1324.

(S11) Mendolicchio, M.; Bloino, J.; Barone, V. General Perturb-Then-Diagonalize Model for the Vibrational Frequencies and Intensities of Molecules Belonging to Abelian and Non-Abelian Symmetry Groups. *J. Chem. Theory Comput.* **2021**, *17*, 4332–4358.

(S12) Barone, V.; Biczysko, M.; Bloino, J.; Puzzarini, C. Accurate molecular structures and infrared spectra of trans-2,3-dideuterooxirane, methyloxirane, and trans-2,3-dimethyloxirane. *J. Chem. Phys.* **2014**, *141*, 034107.

(S13) Yang, Q.; Kapitán, J.; Bouř, P.; Bloino, J. Anharmonic Vibrational Raman Optical Activity of Methyloxirane: Theory and Experiment Pushed to the Limits. *J. Phys. Chem. Lett.* **2022**, *13*, 8888–8892.

(S14) Kreienborg, N. M.; Bloino, J.; Osowski, T.; Pollok, C. H.; Merten, C. The vibrational CD spectra of propylene oxide in liquid xenon: a proof-of-principle CryoVCD study that challenges theory. *Phys. Chem. Chem. Phys.* **2019**, *21*, 6582–6587.

(S15) Fusè, M.; Longhi, G.; Mazzeo, G.; Stranges, S.; Leonelli, F.; Aquila, G.; Bodo, E.; Brunetti, B.; Bicchi, C.; Cagliero, C.; Bloino, J.; Abbate, S. Anharmonic Aspects in Vibrational Circular Dichroism Spectra from 900 to 9000 cm^{-1} for Methyloxirane and Methylthiirane. *J. Phys. Chem. A* **2022**, *126*, 6719–6733.

(S16) Devlin, F. J.; Stephens, P. J.; Cheeseman, J. R.; Frisch, M. J. Ab Initio Prediction of Vibrational Absorption and Circular Dichroism Spectra of Chiral Natural Products Using Density Functional Theory: α-Pinene. *J. Phys. Chem. A* **1997**, *101*, 9912–9924.

(S17) Bogaerts, J.; Desmet, F.; Aerts, R.; Bultinck, P.; Herrebout, W.; Johannessen, C. A combined Raman optical activity and vibrational circular dichroism study on artemisinin-type products. *Phys. Chem. Chem. Phys.* **2020**, *22*, 18014–18024.
